# Tuning of Aromatic Amine Polymer Side chains for Application as Hole Transport Materials in Wide-Bandgap Perovskite Solar Cells

**DOI:** 10.1021/acsapm.6c02188

**Published:** 2026-09-21

**Authors:** Kshitiz Dolia, Ashford Hollis, Andrew Mottur, Tingting Zhu, Tyler Brau, Muhammad Mohsin Saeed, Yifan Yin, Abasi Abudulimu, Randy J. Ellingson, Yanfa Yan, Alan Sellinger, Zhaoning Song

**Affiliations:** † Department of Physics and Astronomy and Wright Center for Photovoltaic Innovation and Commercialization, 7923 University of Toledo, Toledo, Ohio 43606, United States; ‡ Department of Chemistry, 3557 Colorado School of Mines, Golden, Colorado 80401, United States

**Keywords:** functionalized polymers, hole transport materials, aromatic amine, interface properties, perovskite solar cells

## Abstract

Perovskite solar cells (PSCs) hold strong potential for efficient and cost-effective photovoltaic technology, yet their device performance and stability remain hindered by interfacial limitations. In particular, the hole transport material (HTM) that contacts both the perovskite and the front contact can often be a bottleneck for device efficiency and durability. To overcome these challenges, we design and synthesize a series of carbazole- and fluorene-based polymers via palladium-catalyzed Buchwald–Hartwig amination, incorporating tunable *N*,*N*-dimethyl aminopropyl (N) and butanoate (COOCH_3_) side chains. We systematically investigate how these polar side chains affect the electronic properties of the buried perovskite interface and the overall device performance. Our results show that both N and COOCH_3_-functionalized HTMs significantly enhance interfacial compatibility with wide-bandgap perovskites, facilitating superior perovskite film formation and more efficient charge extraction. This enhancement yields wide-bandgap PSCs with a champion power conversion efficiency (PCE) of ∼17%, comparable to devices using commercial poly-triarylamine (PTAA) HTMs. These findings highlight that targeted side-chain engineering offers a simple and effective strategy to optimize polymer HTMs for wide-bandgap PSCs, providing valuable insights for the future design of perovskite optoelectronic devices.

## Graphical abstract

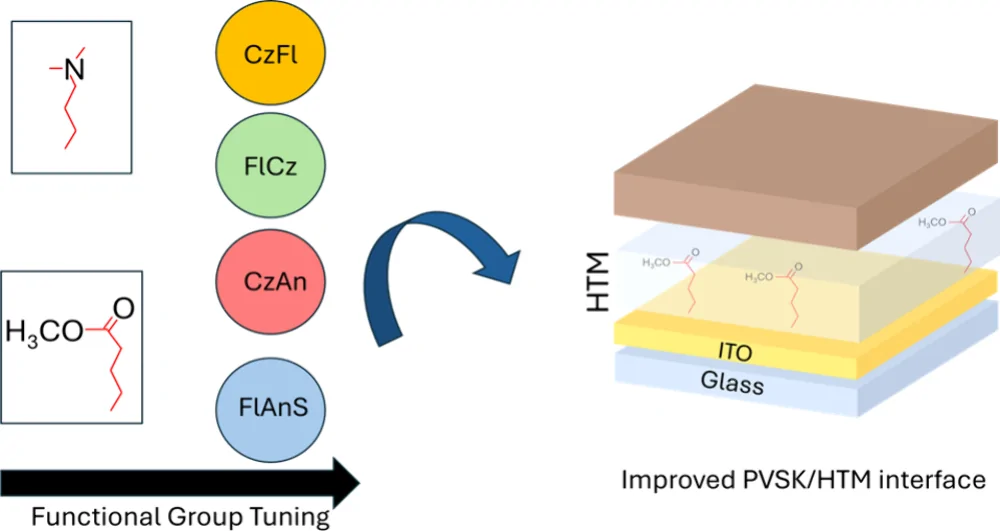

## Introduction

Climate change is here, and it continues to intensify. Rising global temperatures, extreme weather events, and persistent droughts each year underscore the urgent need to transition from fossil fuels to sustainable energy sources. Achieving this transition requires renewable technologies to be low-cost, efficient, scalable, reliable, and capable of sustaining society’s long-term power needs. Over the past decade, perovskite solar cells (PSCs) have emerged as a leading candidate in this regard due to their rapidly increasing power conversion efficiencies, solution processability, and inexpensive manufacturing.^1–4^ Single-junction perovskite devices are now progressing from academic research toward commercialization, rivaling established photovoltaic (PV) technologies.^5,6^ Meanwhile, more efforts have been made to combine perovskite with other photovoltaic technologies, such as c-Si, CIGS, organic or other perovskites to make high-efficiency tandem solar cells.^7–9^ Among them, perovskite/silicon tandem solar cells have already achieved efficiencies exceeding the Shockley-Queisser limit of single-junction devices and are at an all-time high efficiency of 35%.^10^

Despite great promise, the practical deployment of perovskite photovoltaic technologies remains hindered by limited device stability, much of which stems from interfacial incompatibility between constituent layers.^11,12^ Widely used organic hole transport materials (HTMs)—positioned between the perovskite and the front contact—are often hydrophobic. This creates poor compatibility with the perovskite absorber layer, inducing defects and accelerating its degradation.^13^ To make PSCs a truly viable option for generating clean energy, these HTM interface challenges must be addressed.

Commonly used HTMs include functionalized polymers and self-assembled monolayers (SAM) of small molecules. Polymer HTMs (PHTMs), such as poly­(triaryl amine) (PTAA), poly­(3,4-ethylenedioxythiophene):poly­(4-styrenesulfonate) (PEDOT:PSS), poly­(N,N′-bis­(4-butylphenyl)-N,N′-bis­(phenyl)­benzidine) (Poly-TPD), and poly­[3-(4-carboxybutyl)­thiophene-2,5-diyl] (P3CT), often provide good film coverage and mechanical robustness but suffer from molecular weight variability, poor wettability, limited energy tunability, and parasitic absorption.^14–17^ Particularly, PTAA and poly-TPD suffer from high costs, nonwetting issues, and instability, whereas PEDOT:PSS suffers from high acidity, hygroscopicity, and energy band edge mismatch with Pb perovskites.^18^ SAMs provide an ultrathin coating of HTM with tunable energy levels, minimizing optical and electrical losses at the interface, yet their interfacial chemistry and long-term stability are highly sensitive to surface preparation and molecular structure.^19^ Considerable effort has been devoted to molecular doping, interfacial modification, and compositional optimization of these HTMs. For instance, nearly all polymer HTMs have been improved over time either externally by adding dopants during fabrication or internally by modifying the side chains and functional groups. Xie et al. replaced the *n*-butyl functional group with the isobutyl group to improve the hole transport ability of poly-TPD.^20^ P3CT is another example where the terminal methyl group of the hexyl side chain in poly­(3-hexylthiophene-2,5-diyl) (P3HT) is replaced with a carboxylic acid group to enhance surface wettability and anchoring to the transparent conducting oxide (TCO) front electrode. P3CT has been further modified by replacing the acidic proton with sodium (Na^+^), potassium (K^+^), cesium (Cs^+^), lead (Pb^2+^), and 1,3,5-tris­(bromomethyl)­benzene (TBB), resulting in improved device performance.^14,21,22^ Site-specific functionalization has also been demonstrated to improve energy alignment with mixed-halide perovskites and reduce voltage losses.^23,24^ However, challenges persist in achieving simultaneously high hole conductivity, favorable energy-level alignment, strong interfacial adhesion, minimized parasitic photon absorption, and reproducible perovskite film growth.^25,26^

Rational molecular design of HTMs therefore represents a powerful strategy to overcome these limitations. In this work, we synthesize a series of PHTMs functionalized with distinct chemical moieties to systematically tailor their interfacial and electronic properties. Particularly, we show that the addition of polar side-chain moieties, *N*,*N*-dimethyl aminopropyl (N) and butanoate (COOCH_3_), to the backbone of an existing family of PHTMs^27^ substantially enhances PHTM wettability and interfacial interactions, promoting uniform, high-quality wide-bandgap perovskite film formation and energetically favorable hole extraction. These improvements translate directly into enhanced device efficiency, reproducibility, and stability. Our results highlight the importance of precise molecular engineering in PHTM design and demonstrate how targeted functionalization can simultaneously address interfacial chemistry and charge-transport requirements in high-efficiency wide-bandgap PSCs for tandem application.

## Results and Discussion

### Functionalized PHTMs with Wettable Monomer Addition

The interface between the perovskite layer and HTM is a crucial aspect of device operation. Previous work performed by Astridge et al.^28^ has developed a family of PHTMs that rival industry-standard PTAA in terms of device performance and could be manufactured at lower costs. This study outlines the synthetic pathways taken to produce the wettable monomers and derivatives of previously reported carbazole (Cz) and fluorene (Fl) based polymers.^29^ The synthesis used the Buchwald–Hartwig amination^30,31^ to integrate a triaryl amine moiety into the backbone of the polymer, leading to similar electron-rich and conductive properties to that of PTAA and in many cases outperforming PTAA.^28^ To further improve the stability of the interface between the HTM and the perovskite layer, wettable moieties were incorporated into these polymers (Scheme 1). Each wettable monomer will have differing affinities for the ITO and perovskite layers based on the polarity and steric properties of the moiety. The N monomer provides the electron lone pairs on the nitrogen terminal side-chain moiety that interacts with the Pb in the adjacent perovskite layer. The polar nature of the terminal COOCH_3_ methyl ester group is hypothesized to interact with both the perovskite and ITO layers, promoting enhanced interfacial interactions.

**Scheme 1:**
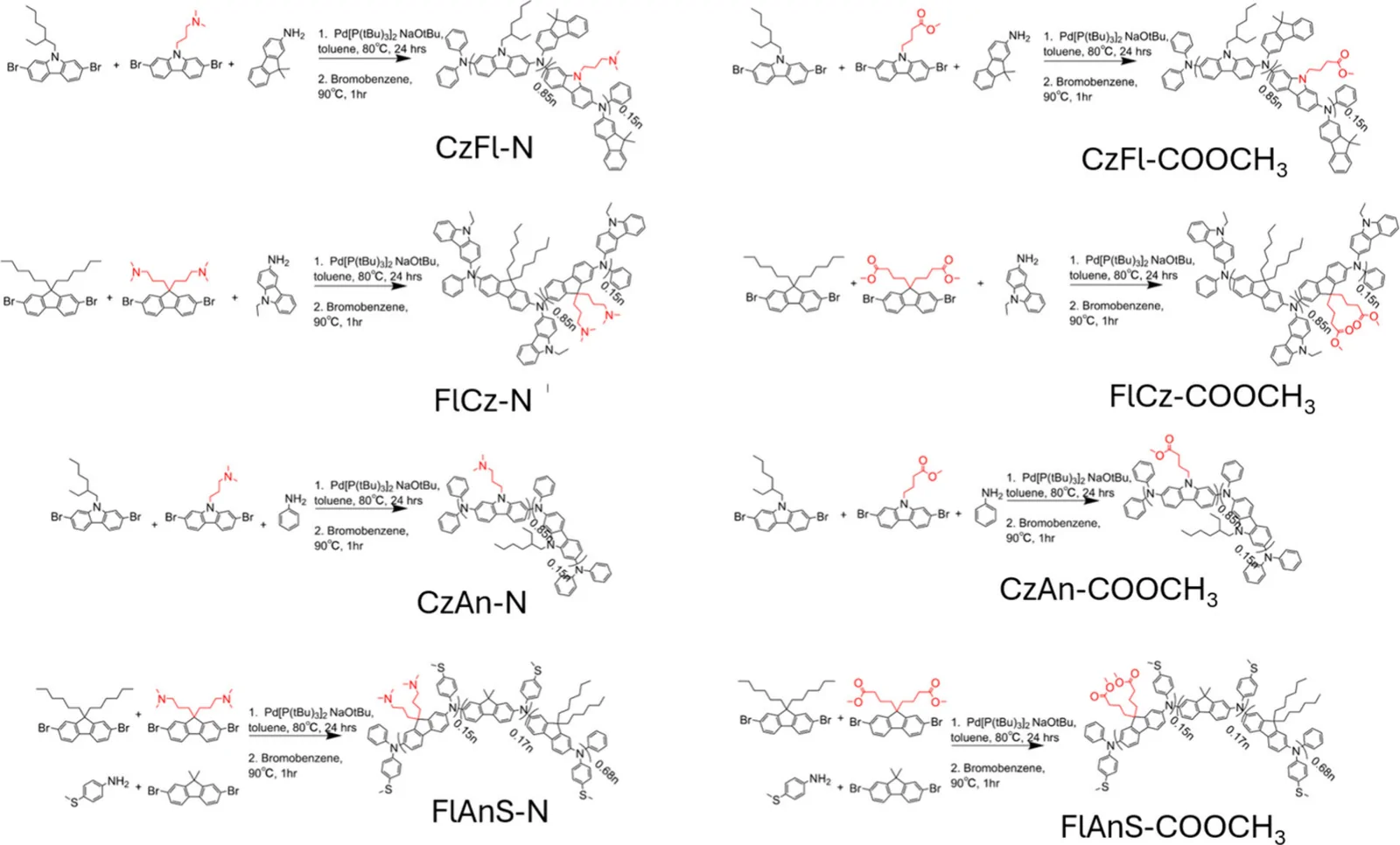
Reaction Schemes for the Synthesis of the Side-Chain Modified (−N and −COOCH_3_) Polymer Hole Transport Materials CzFl, FlCz, CzAn, and FlAnS Used in This Study

We screened a family of polymers for this study and down-selected to those having backbones of CzFl, FlCz, CzAn, and FlAnS with N and COOCH_3_ monomers added to the polymer backbone in 15 mol % feed ratios unless otherwise stated. The effect of this differing feedstock was measured in terms of ^1^H NMR analysis (Figures S1–S11), thermal analysis (Figures S12–S27), optical density (Figures S28–S35), work function (Figure S36), molecular weight (Figure S37), and contact angle (Figures S38–S41) measurements. Some relevant properties for the synthesized PHTMs are summarized in Table 1, and others can be found in the Supporting Information.

**Table 1:** Electronic, Physical and Thermal Properties of Various PHTMs

HTM	optical bandgap energy (eV)	HOMO (eV)	work function (eV)	*M*_n_ (kDa)	polydispersity index	*T*_d_ (°C)	*T*_g_ (°C)
CzFl	2.86	–4.95	4.76	4.0	2.41	420	162
CzFl-N	2.83		4.83	1.5	1.86	438	182
CzFl-COOCH_3_	2.85		4.84	4.5	2.55	428	185
FlCz	2.81	–4.97	4.73	23.6	2.42	421	162
FlCz-N	2.8		4.79	1.6	2.2	406	167
FlCz-COOCH_3_	2.77		4.73	5.4	2.06	412	130
CzAn	2.94	–4.95	4.73	10.1	5.99	362	146
CzAn-N	2.93		4.72	1.7	1.49	418	137
CzAn-COOCH_3_	2.9		4.75	5.1	2.96	443	145
FlAnS	2.9	–5.18	4.87	14.6	2.63	408	120
FlAnS-N	2.9		4.97	1.5	1.73	401	153
FlAnS-COOCH_3_	2.87		4.97	6.9	2.64	454	135
PTAA^32^	2.95	–5.25	4.87	10–115	1.4–3.1	500	102

In this study, ^1^H NMR spectroscopy served as the primary method for polymer structural analysis. Due to the complexity of having more than 35 protons per repeat unit and varying ratios of polymers to wettable moieties, identifying the detailed polymer structure proved particularly challenging. Given the assumption that the wettable side chains would not interfere with the reactivity of the Buchwald–Hartwig amination polymerization, it was anticipated that full incorporation of the wettable moieties would occur, in this case, up to 15 mol %.

The degree of wettable monomer incorporation was estimated by comparing integrations representative of the tertiary amine-containing side chain and that of the ethylhexyl chain in the parent polymer, specifically in the 1.7 ppm region. For the parent CzAn polymer, this region corresponds to the single proton located on the tertiary carbon on the ethylhexyl side chain, which has 1 as the peak integration value. Following incorporation of the wettable monomer, the corresponding peak increased to an integration value of approximately 1.85, consistent with the addition of tertiary amine methyl protons from the incorporated wettable moiety.

Based on the expected contribution of six additional protons per incorporated wettable monomer, the observed integration corresponds to approximately 15 mol % incorporation. This same logic was used for the other polymers as well. Typical impurities found with the NMR structure would always represent solvent peaks, which would be removed via excess drying. This indicates that the repetitive precipitation purification method, including a palladium scavenger step, was successful in removing impurities.^33^ The 8 full proton NMR spectra of the complete wettable PHTM library can be found in Figures S1–S8. Figures S9 and S10 show the NMR spectra of parent polymers. A comparison of the wettable polymers to the parent polymers can be found in Figure S11.

It was hypothesized that introducing polar side chains to the polymer backbone is a simple and effective means of increasing interface interactions between the HTM and the perovskite layer without significantly altering the optoelectronic and thermal properties of the polymer. This was observed when the characterization of the functionalized PHTMs showed a minimal deviation from the parent polymer’s thermal and optical properties. The thermal stability of the synthesized HTMs was evaluated by thermogravimetric analysis (TGA) for decomposition temperatures (*T*_d_), as shown in Figures S12–S19, and differential scanning calorimetry (DSC) for glass-transition temperatures (*T*_g_), as shown in Figures S20–S27. The observed *T*_d_ values (362–454 °C) significantly exceed standard photovoltaic operating conditions, suggesting that thermal decomposition is unlikely to compromise device integrity. Furthermore, *T*_g_ values ranged from 120 to 185 °C, influenced by variations in molecular weight and polymer backbone rigidity. Because these values remain well above the *T*_g_ of PTAA (∼100 °C), they provide the necessary resistance to morphological changes, thereby enhancing the long-term stability of the PSCs.

UV-vis spectroscopy (Figures S28–S35) was used to obtain the absorbance wavelength of polymers, with the absorbance data being converted into the optical bandgap energy, i.e., the energy difference between the highest occupied molecular orbital (HOMO) and the lowest unoccupied molecular orbital (LUMO) levels. The variability of the polymer backbones in this family of PHTMs allows compatibility with different perovskite active layer formulations, i.e., low-, medium-, and wide-bandgap. The HOMO and LUMO levels of the HTM need to align with that of the adjacent perovskite and ITO layers to ensure the flow of holes while maintaining an energy barrier to block electrons from the anode. The wide band gap range of these polymers (2.77–2.95 eV) favors this quality.^13^ As seen in Figure 1 and Table 1, the addition of the wettable moieties changed the optical bandgap of the PHTMs by at most 0.04 eV. This shift is not significant enough to affect the energetic alignment of these PHTMs with their adjacent layers. These demonstrate that conjugation of the polymer backbone is the driving force behind changes in the optical bandgap, and, therefore, the addition of wettable side chains should have minimal effect. For this reason, only the HOMO levels of the parent polymers, measured via photoelectron spectroscopy, are reported.^28^

**Figure 1:**
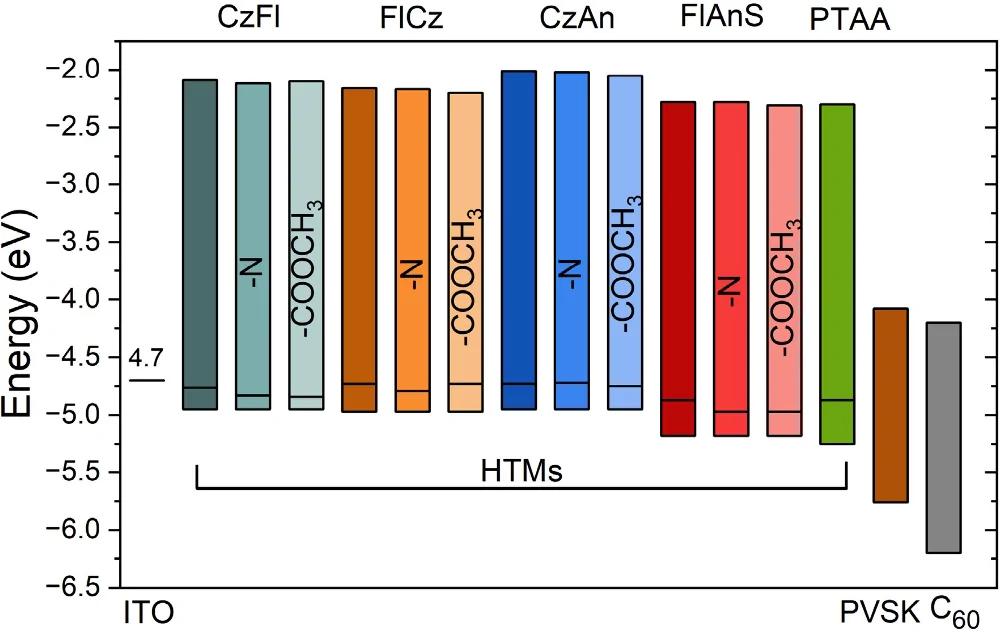
Energy level alignment of the inverted perovskite solar cell architecture. The diagram illustrates the HOMO and LUMO levels of the various PHTM families relative to the ITO anode, the perovskite (PVSK) absorber, and the C_60_ electron transport layer.

The Fermi level of various PHTMs is calculated by converting the contact potential difference (CPD) measured by KPFM on the glass/ITO/PHTMs sample to work function using the glass/ITO/Au sample as a reference (Figure S36). While the HOMO levels are inferred to remain relatively invariant upon the introduction of wettable side chains, the Fermi level of some PHTMs shifts slightly toward deeper energy levels (increased work function). Notably, FlAnS PHTM exhibits a deeper HOMO level than its counterparts, closely aligning with that of PTAA. The incorporation of wettable moieties induces a more pronounced downward shift in the Fermi level for this specific polymer. Interestingly, an increase in work function was observed for both the nominally electron-donating −N­(CH_3_)_2_ group and the electron-withdrawing C=O group for most polymers. This phenomenon is likely a consequence of the propyl spacer, which decouples the functional groups from the conjugated backbone and suppresses the resonance-based electron-donating nature of the amine. This structural isolation allows the inductive effect and the resulting surface dipoles to dominate the interfacial energetics. Such an increase in work function is expected to mitigate the interfacial energy barrier and facilitate a more ohmic contact at the HTM/perovskite interface. This optimized alignment promotes efficient hole extraction, ultimately manifesting as a reduction in transport resistance within the resulting perovskite devices.^34,35^

Molecular weight analysis was performed on each PHTM to determine if the presence of the wettable side chains interfered with the Buchwald–Hartwig amination polymerization and inhibited chain growth. As seen in Figure S37, the average *M*_n_ of the PHTM materials containing wettable moieties is noticeably lower than that of their parent polymers, and in the case of FlCz and FlAnS it is decreased by an order of magnitude. This indicates that the addition of the wettable moieties affects polymerization. The N polymers experience the largest decrease in chain length. This is likely due to interference from the tertiary amine on the wettable side chain disrupting chain growth by interacting with the catalyst, though the exact mechanism is still undetermined. The high selectivity of the Pd catalyst indicates that there should be very few side interactions occurring with the remaining wettable moieties.^36,37^ Therefore, the presence of the COOCH_3_ moieties should not have significantly affected the chain growth of the polymers. Further exploration is required to determine the cause of the decreased *M*_n_ observed in the wettable polymers. For all PHTMs with wettable moieties, the wettability measurements (Figures S38–S41) show enhanced hydrophilicity, with water contact angle ranging from 11° for CzFl-N to 36° for FlAnS-COOCH_3_. This increased wettability is expected to facilitate the spreading, nucleation, and crystallization of the perovskite precursor solution during film deposition.

### Characterization of Perovskite Materials and Devices

The ideal bandgap for the perovskite top cell in the perovskite/silicon tandem solar cell is within 1.68–1.70 eV for optimal light coupling.^38,39^ Here, we selected a 1.68 eV perovskite with an MA-free composition of FA_0.78_Cs_0.22_Pb­(I_0.8_Br_0.2_)_3_ to study the effects of various PHTMs.^40^ To evaluate the impact of PHTM functionalization on solar cell performance, we fabricated PSCs using four distinct polymer families (CzFl, FlCz, CzAn, and FlAnS) with −N or −COOCH_3_ modifications. To ensure a consistent screening process, all polymers were dissolved in chlorobenzene (2 mg/mL) and spin-coated under identical conditions. No additional interface modification layers were used. Atomic force microscopy (AFM) measurements of the PHTMs deposited on ITO substrates revealed uniform, smooth morphologies with surface roughness ranging from 1.10 to 1.55 nm (Figure S42). Such smoothness is critical for achieving a high-quality, uniform perovskite film.

We evaluated wettability of the perovskite precursor solution and then spin-coated perovskite films onto various PHTMs. Figure S43 shows the contact angle measurements using the perovskite precursor solution. Figure S44 presents photographs of perovskite films deposited on 1-inch ITO/PHTM substrates. Figure 2 compares the perovskite precursor coverage and contact angles of the precursor solution on PHTMs. The results indicate that the functional groups significantly enhance the wettability of PHTMs, facilitating surface coverage of the perovskite precursor solution. This improved wettability is particularly evident in the FlCz, CzAn, and FlAnS families, outperforming the PTAA standard. Such good wettability is a vital prerequisite for the scalable coating processes used for scaling up module production.^41^

**Figure 2:**
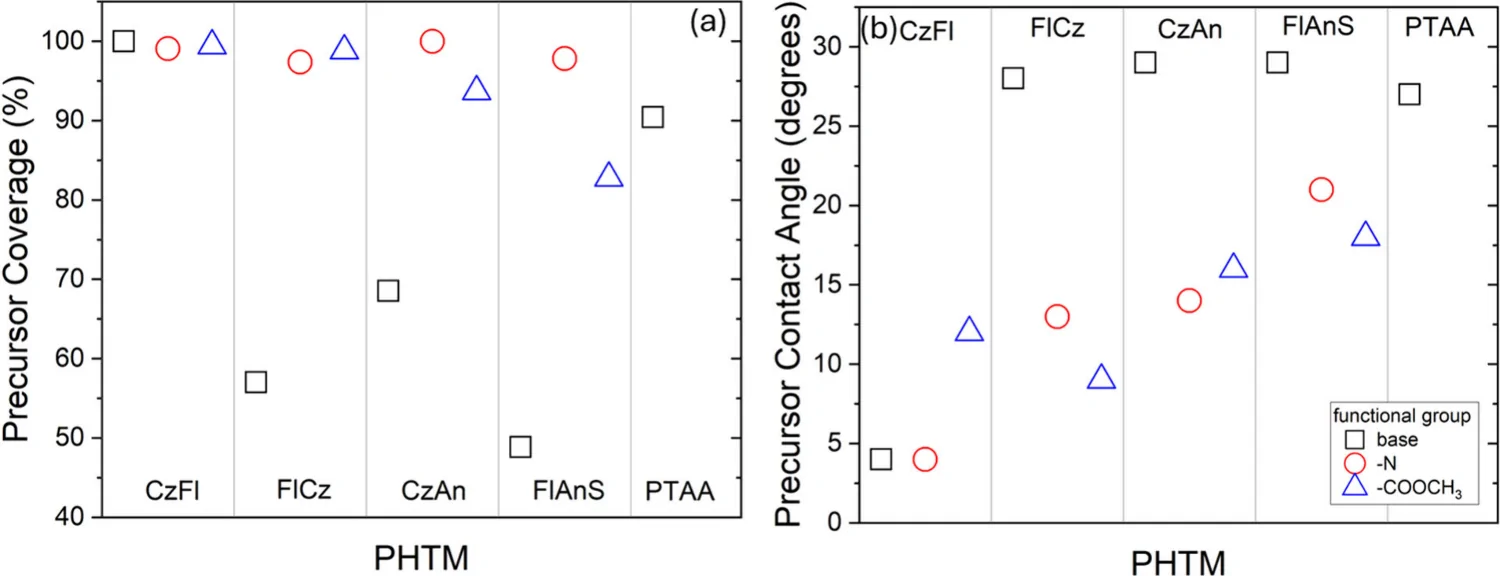
Wetting characteristics and film formation of the perovskite precursor on various polymer surfaces. (a) Surface coverage percentage of perovskite films and (b) corresponding contact angle measurements for the four PHTM families compared to the PTAA benchmark. The legend indicates the functionalization status: unfunctionalized base polymers (black squares), −N modified (red circles), and −COOCH_3_ modified (blue triangles).

To better assess the impact of the PHTMs on the growth and optoelectronic properties of perovskite films, we further characterize the perovskite materials deposited on various PHTM substrates. Figure S45 compares the XRD patterns of these perovskite films, all of which exhibit the cubic perovskite phase. The most intense diffraction peaks appear at ∼14° and ∼21°, corresponding to the (100) and (110) planes, respectively. The absence of common impurity phases, such as PbI_2_ and the hexagonal δ-phase, confirms the formation of phase-pure perovskite on these PHTMs. Notably, the crystallinity of perovskite films varied across the different PHTMs (Table S1), but, in general, polymers with more wettable moieties yielded higher XRD peak intensities compared to their parent polymers. This suggests that enhanced surface wettability facilitates improved perovskite crystallinity.^42^ It is worth noting that the surface wettability of the functionalized polymers is much better than that of PTAA. Therefore, no interfacial wetting layers are needed, which ensures that the observed crystal growth is intrinsic to the polymer surfaces, independent of auxiliary materials or interfacial modifiers.^43^

Photoluminescence (PL) measurements assessed the effect of PHTMs on charge carrier recombination and extraction in the perovskite layer. Figure S46 compares the PL spectra of perovskite films deposited on various PHTMs. In general, the perovskite film grown on PTAA exhibits the highest PL intensity, indicating the largest quasi-Fermi level splitting among the group. For the CzFl family, incorporating wettable moieties resulted in an increased work function approaching that of PTAA and an enhanced PL intensity. These functional groups are proposed to modify the band energy alignment at the perovskite/HTM interface, thereby suppressing interfacial recombination. In contrast, wettable modifications to the FlAnS series led to an increased work function compared to PTAA but a reduction in PL intensity. The observed PL quenching is most likely due to enhanced hole extraction governed by the deeper work function of FlAnS. The FlCz and CzAn polymer families exhibited increased variability, which was probably caused by a complicated interaction between defect suppression^44^ and charge extraction^45,46^ that is still challenging to separate.

To evaluate the impact of PHTM functionalization on device performance, we fabricated perovskite solar cells with an inverted architecture of glass/ITO/HTL/perovskite/C_60_/SnO_*x*_/Ag. Figure 3 displays the J-V parameters of the devices prepared with different PHTMs. A PHTM concentration of 2 mg/mL was used after optimization for uniform comparison of devices. The performance variation with PHTM concentration for CzFl-N is shown in Figure S47. All wide-bandgap PSCs based on PHTMs exhibited decent device performance, except for CzAn-N (*vide infra*). The power conversion efficiencies (PCEs) are in the range of 14 to 17%, comparable to the 17% benchmark efficiency achieved using commercial PTAA (Figure 3 and Table S2), highlighting the potential of these low-cost, modifiable polymers. *J*–*V* data for all the devices made with different polymer HTMs could be found in Tables S4–S7 with *J*–*V* plots for champion devices in Figures S48–S52.

**Figure 3:**
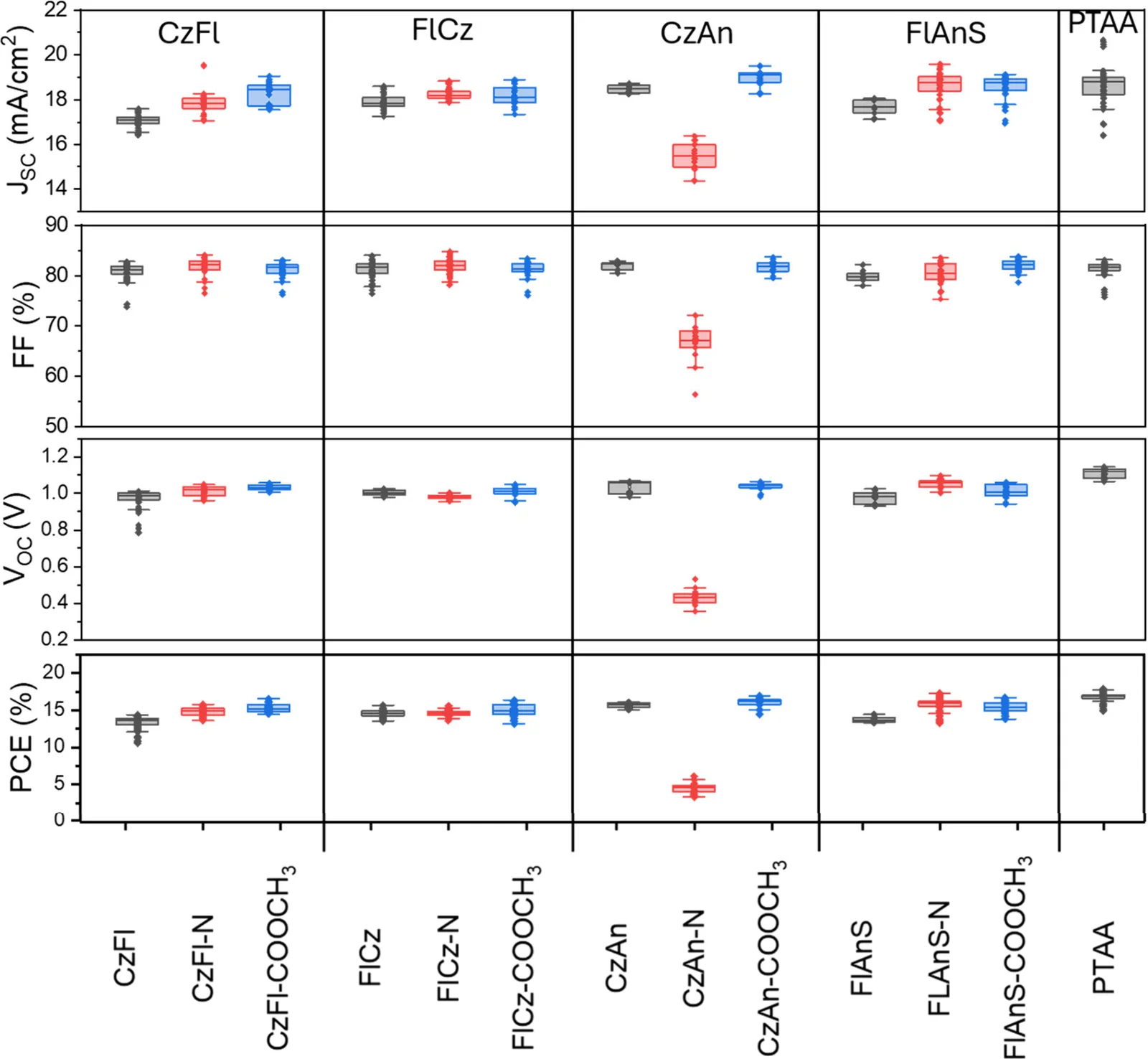
*J*–*V* parameters of PSCs made with different polymer HTMs. Error bars represent the standard deviation. Data presented were measured in reverse bias.

External quantum efficiency (EQE) measurements were performed to evaluate the influence of different PHTMs on charge carrier generation and collection in PSCs (Figure S53). Overall, the integrated photocurrent densities are in good agreement with the *J*_SC_ values determined from *J*–*V* scans. Adding wettable moieties to CzFl and FlAnS families notably improved EQE, suggesting better buried interface quality. In contrast, FlCz and CzAn families had minor EQE gains, except for CzAn-N, which showed a marked decrease. Among all tested samples, FlAnS-N yielded the highest integrated *J*_SC_ of 19.68 mA/cm^2^, demonstrating superior charge carrier collection. Although the bandgaps of the various PHTMs vary (2.8–2.9 eV), they appear to have a negligible impact on the EQE profile at short wavelengths. This is likely due to the nm scale thickness of the PHTMs (estimated at <10 nm), which minimizes parasitic absorption. Furthermore, the EQE changes occurred uniformly across all wavelengths, suggesting that the underlying HTL primarily influences the charge collection efficiency of the front region of the perovskite absorber.^47^

The inferior PV performance and reduction in EQE in CzAn-N devices are likely attributable to the polymer’s relatively narrow PDI and low *M*_n_, which render the layer susceptible to interfacial mixing and solvent-induced erosion during perovskite deposition.^48^ We confirmed this hypothesis using KPFM to compare the work functions of the CzAn-N substrate, the perovskite-coated substrate, and a substrate where the perovskite layer was washed off with a solvent (Figure S54). The work function of the washed CzAn-N substrate (5.24 eV) was closer to that of UV-ozone treated bare ITO (5.3 eV) than to the pristine CzAn-N work function (4.86 eV), indicating that the PHTM layer was largely removed or compromised by the perovskite precursor solvent. Due to the absence of the PHTM, all PV parameters are substantially reduced.

Returning to the discussion of PHTM modification effects on PSC performance, Figure 4 presents a comparative analysis of changes in photovoltaic parameters for each PHTM family functionalized with distinct groups (see Table S3 for details). For the CzFl family, *V*_OC_ and *J*_SC_ increased following modification with −N and −COOCH_3_ moieties, resulting in PCE improvements of 11% and 15%, respectively. For the FlCz family, -N functionalization reduced *V*_OC_ by ∼2.5%, while −COOCH_3_ increased it by ∼1%. Both modifications raised *J*_SC_ and FF by 1–2%, yielding an overall PCE increase of 1–2%. In the CzAn family, −N functionalization significantly reduced device performance, probably because the HTM dissolved in the perovskite precursor solution, as previously mentioned. In contrast, −COOCH_3_ modification led to a 2–3% improvement in PCE compared to the original polymer. For the FlAnS family, each PV parameter showed a 4–9% rise, contributing to a 15–17% boost in PCE for both functionalized polymers. Among all tested PHTMs, devices based on FlAnS-N deliver the highest PCE (∼17%), comparable to PSCs based on PTAA (Figure 3). Its outstanding performance results from a deeper HOMO level that matches well with the wide-bandgap perovskite’s valence band edge, as well as improved wettability that encourages the formation of high-quality grains. It is important to note that, although the optoelectronic properties of the various PHTMs differ, adding these wettable moieties, particularly −COOCH_3_, consistently enhances device performance compared to the original polymers.

**Figure 4:**
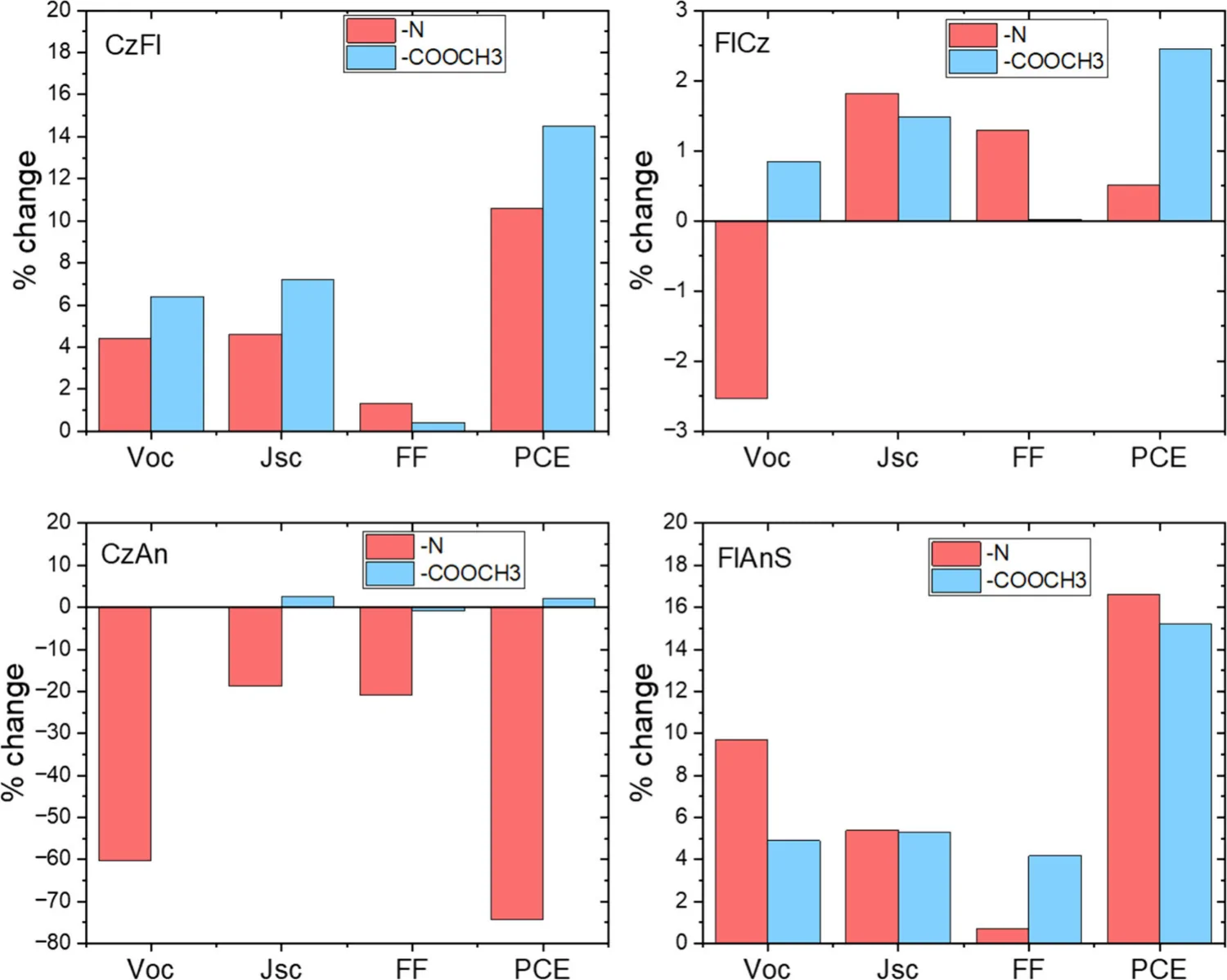
Relative percentage change in average photovoltaic (PV) parameters (*V*_OC_, *J*_SC_, FF, and PCE) for devices using functionalized PHTMs compared to their respective parent polymers. The red and blue bars represent the effects of −N and −COOCH_3_ functionalization, respectively, across the four polymer families.

Finally, we examined how PHTMs influence the operational stability of wide-bandgap PSCs. Stability was measured using maximum power point tracking (MPPT), as shown in Figure S55. The stability profiles for all PHTM variants closely resemble those of devices using PTAA. These findings demonstrate that choosing appropriate PHTMs for PSCs cannot rely on a single factor or limited criteria, highlighting the importance of a comprehensive investigation considering all relevant trade-offs.

## Conclusions

In summary, four families of PHTMs were synthesized using Buchwald–Hartwig amination and subsequently evaluated in wide-bandgap (1.68 eV) PSCs appropriate for perovskite/silicon tandem applications. Our findings indicate that side-chain functionalization markedly improves the surface wettability of these polymers, enabling consistent and uniform coating of perovskite films. The synthesized PHTMs demonstrated performance levels comparable to those of control devices utilizing commercial PTAA. Notably, the butanoate (−COOCH_3_) functional group reliably improved perovskite/HTM interface characteristics and facilitated enhanced charge extraction at the interface. This study establishes side-chain functionalization as a viable approach for improving both the performance and processing compatibility of polymer hole transport materials in PSCs. Looking forward, this strategy may be integrated with other materials to further advance device efficiency.

## Supplementary Material



## Notes

Experimental details; Wettable polymer synthesis; NMR, TGA, DSC, UV-vis, and work function measurements; Comparison of PHTM Mn; Water contact angle, AFM, precursor contact angle, precursor film coverage, perovskite film and device characterization; Tables of device characteristics (PDF)
