# Supplementary material for: Tuning of Aromatic Amine Polymer Side chains for Application as Hole Transport Materials in Wide-Bandgap Perovskite Solar Cells
Source: ACS Appl Polym Mater. 2026 Sep 21;8(19):17043–51. doi: 10.1021/acsapm.6c02188 (PMC13660351; doi:10.1021/acsapm.6c02188)
Supplement: Supplementary file 1 [file ap6c02188_si_001.pdf]

Supporting Information

Tuning of Aromatic Amine Polymer Sidechains for Application as Hole Transport Materials in Wide-Bandgap Perovskite Solar Cells

Kshitiz Dolia[1,√], Ashford Hollis[2,√], Andrew Mottur[2], Tingting Zhu[1], Tyler Brau[1], Muhammad Mohsin Saeed[1], Yifan Yin[1], Abasi Abudulimu[1], Randy J Ellingson[1], Yanfa Yan[1], Alan Sellinger[2,*] and Zhaoning Song[1,*]

1. Department of Physics and Astronomy & Wright Center for Photovoltaic Innovation and Commercialization, University of Toledo, Toledo, OH 43606, United States
2. Department of Chemistry, Colorado School of Mines, Golden, CO 80401, United States

√ Each author contributed equally.

* Corresponding authors: aselli@mines.edu, zhaoning.song@utoledo.edu

Contents

S1.	Experimental Details	S-2
S2.	Wettable Polymer Synthesis	S-4
S3.	^{1}H NMR	S-12
S4.	TGA	S-18
S5.	DSC	S-22
S6.	UV-Vis and Work function Measurements	S-26
S7.	Comparison of PHTM M_n	S-32
S8.	Water Contact Angle	S-30
S9.	AFM	S-33
S10.	Precursor Contact Angle	S-34
S11.	Precursor Film Coverage	S-35
S12.	Perovskite Film and Device Characterization	S-36
S13.	Tables of Device Characteristics	S-42

S1. Experimental details

Materials: All materials for polymer synthesis were used as received from Millipore-Sigma unless otherwise stated. 2,7-Dibromocarbazole was provided by Seqens-PCAS. 2,7-dibromo-9,9-dihexyl-9*H*-fluorene was purchased from Combi Blocks. Palladium catalysts were purchased from Strem Chemicals. Aniline was purchased from Acros Organics and vacuum distilled over KOH according to literature procedures.[1] 3-Amino-9-ethyl carbazole was purchased from Alfa Aesar and used as received. 9,9-dimethyl-9*H*-fluoren-2-amine and 4-(methylthio)aniline were purchased from Ambeed and used as received. All raw materials for device fabrication were directly used without further treatment unless otherwise stated. The solvent of N,N-dimethylformamide (DMF, 99.8%), dimethyl sulfoxide (DMSO, 99.9%), deionized (DI) water (H_2O), acetone (99.5%), isopropanol (IPA, 99.5%), methanol (Me-OH, 99.5%), anhydrous ethanol (Et-OH, 99%), anisole (99%), and chlorobenzene (CB, 99.9%) were purchased from Sigma–Aldrich. The organic halide salt of formamidinium iodide (FAI) was obtained from Greatcell Solar Company. Bathocuproine (BCP), lead iodide (PbI_2), lead bromide ($PbBr_2$), and 1,3-propane-diammonium iodide ($PDAI_2$) were bought from TCI company. Cesium iodide (CsI), lead chloride ($PbCl_2$), methylammonium chloride (MACl), and poly[bis(4-phenyl)(2,4,6-trimethylphenyl)amine (PTAA) were bought from Sigma–Aldrich. Fullerene (C_{60}) powder was purchased from Nano-C. Atomic layer deposition (ALD) precursor of tetrakis(dimethylamino) tin (IV) (99.99%-Sn, 50-1815 Tin) was bought from Strem Chemicals. Silver (Ag) beads were purchased from Kurt J. Lesker Company.

Polymer Synthesis: The synthesis of polymers CzFl, FlCz, CzAn and FlAnS has been reported in our previous work.[2] The polymers introduced here were synthesized using a 1.00:1.02 equivalent of dibromoaryls to aromatic amine, with the exception of the polymers using CzFl, which utilized a 1:1.05 stoichiometric ratio of dibromoaryl to aryl amine. Using a 5 mol % excess of 9,9-dimethyl-9*H*-fluoren-2-amine was due to the high molecular weight of the polymers when synthesized using a 2 mol % excess, making solubility of the polymer an issue. In all procedures, end capping was done with bromobenzene. The detailed polymer synthesis procedure can be found in the S2. Wettable polymer synthesis.

Perovskite precursor preparation: A perovskite precursor with the composition of $FA_{0.78}Cs_{0.22}Pb(I_{0.82}Br_{0.18})_3$ (1.4 M) was prepared by mixing CsI (62.4 mg), FAI (165.12 mg), $PbCl_2$(16.68 mg), MACl (4.05 mg), and $PbBr_2$ (264.24 mg), PbI_2 (221.3 mg) in DMF:DMSO (800 µL:200 µL) mixed solvent.

Fabrication of perovskite solar cells (PSCs): PSCs were fabricated using the structure of glass/ITO/HTL/perovskite/C_{60}/SnO_x/Ag. The ITO patterned glass substrates were sequentially cleaned with acetone and IPA in an ultrasonic bath for 30 min each. Before PSCs fabrication, the cleaned ITO glass was first treated in a UV–ozone chamber for 30 min. The samples were then

moved into a nitrogen glovebox where an 85 μL solution of PHTM (2 mg mL^{-1} in chlorobenzene) was spin-coated at 4000 rpm for 30 s, followed by annealing at 100 °C for 10 min. Then, the perovskite precursor solution was spin-coated in two steps. The first step is spinning at 300 rpm for 5 s, and the second step is 30 s at 5000 rpm. During the second step, nitrogen gas is applied through a nozzle after 18 s. The samples were then annealed at 100 °C for 10 min. Then, 65 μL of $PDAI_2$ (1 mg mL^{-1} in IPA) was dynamically coated on the cooled perovskite as surface passivation. After that, the substrates were moved to a thermal evaporation chamber where 25 nm of fullerene (C_{60}) was deposited. The samples were then deposited with 15 nm of SnO_x using ALD at a substrate temperature of 85 °C. The samples were then deposited with 80 nm of Ag using thermal evaporation at room temperature.

Characterization: UV−vis absorption spectra were obtained on a Beckman Coulter DU 800 spectrophotometer using chloroform as a solvent. Emission spectra were obtained using a Horiba Jovin Yvon Nanolog spectrofluorometer with chloroform as a solvent. ^{1}H NMR spectra were acquired using a JEOL ECA-500, with benzene-d_6 as a solvent for all polymers and chloroform-*d* for all monomers. Gel permeation chromatography (GPC) was performed on a Polymer Laboratories GPC 200, using THF as a solvent and polystyrene standards to obtain the number-average molecular weights (M_n) of each polymer. MSE PRO standard contact angle meter/goniometer was used to collect water contact angle measurements on polymer thin films using a 10-picture average to determine the contact angle. Precursor contact angle on polymer films was measured after 5 minutes of settling time using Keyence VR-6000 optical microscope. Thermal properties were determined via thermal gravimetric analysis (TGA) using a Q500 TA Instrument. The decomposition temperature (T_d) of the polymers was determined by ramping 10 °C/min to 600 °C under an inert nitrogen atmosphere. Differential scanning calorimetry (DSC) was performed to determine the glass transition temperature (T_g) using a TA Instruments Q2000 at a scan rate of 10 °C/min under nitrogen flow from -20°C to 200°C, with T_g transition obtained from the second cycle. The XRD measurements were conducted on the Rigaku Ultima III with Cu K∝ radiation. Photoluminescence (PL) measurements were done in a custom-built system with a Horiba Symphony-II CCD detector and iHR320 monochromator photoexciting the samples with a 532 nm continuous wave laser (6 mW cm^{-2}). The J–V characteristics of perovskite solar cells were measured with a Keithley 2400 series source meter under a solar simulator (Sunbrick, G2V) calibrated to AM1.5G radiation. The EQE measurements were done using a PV Instruments system (model IVQE8-C). The atomic force microscopy (AFM) and kelvin probe force microscopy (KPFM) data of various films were collected using a Veeco D500 atomic force microscope.

S2. Wettable Polymer Synthesis

Note: All thermal gravimetric analyses were determined via program calculations of the tangent onset point. Differential scanning calorimetry was determined via program calculations of the tangent at the glass transition temperature. Water contact angle measurements were determined via program calculations of the average between the left and right sides of the droplet.

<u>CzFl-N</u>

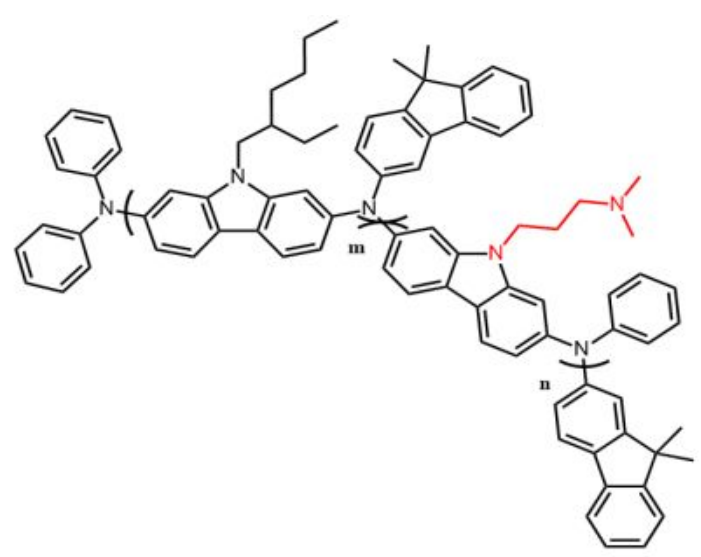

A 50 mL Schlenk flask and stir bar were oven dried. The flask was charged with 2,7-dibromo-9-(2-ethylhexyl)-carbazole (0.767 g, 1.75 mmol), 2-amino-9,9-dimethylfluorene (0.453 g, 2.17 mmol), NaOtBu (0.700 g, 7.28 mmol), 2,7-dibromo-9-(3-(dimethylamino)propyl)-carbazole (0.127 g, 0.309 mmol) and bis(tri-tert-butylphosphine)palladium(0) (0.05 g, 0.097 mmol) followed by vacuum/N_2 refill cycles (3x). Anhydrous toluene (10 mL) was added with stirring via syringe and the reaction was heated to 80°C for 18 hours. The polymer was end-capped with bromobenzene (0.013 mL, 0.122 mmol) and heated to 90°C for 1 hour. The reaction was cooled to room temperature and was precipitated dropwise into 200 mL cold stirring methanol. The reaction flask was rinsed with 2 mL THF and added dropwise to the methanol. A bright yellow/green precipitate was collected by vacuum filtration and dried overnight in a vacuum oven at 50°C and then dissolved in minimal THF to which diethylammonium diethyldithiocarbamate (0.017 g, 0.075 mmol) was added as a Pd scavenger. This was stirred at reflux for 2 hours. The solution was cooled to room temperature and precipitated into 200 mL stirring methanol and the solids were recovered by vacuum filtration and dried overnight in a vacuum oven at 50°C to achieve a pure light green product (81.5%). ^{1}H NMR (500 MHz, Benzene-d_6) δ 8.01 – 7.79 (m, 2H), 7.71 – 7.33 (m, 9H), 7.21 (d, J = 7.3 Hz, 3H), 3.70 – 3.38 (m, 2H), 1.76 (s, 2H), 1.44 – 1.19 (m, 8H), 1.13 – 0.75 (m, 10H), 0.63 (q, J = 7.0 Hz, 3H), 0.50 (t, J = 7.5 Hz, 3H). Thermal analysis (T_g=182℃, T_d=438℃). UV-Vis Absorbance wavelength = 402 nm. Optical band gap energy = 2.83 eV.

CzFl-$COOCH_3$

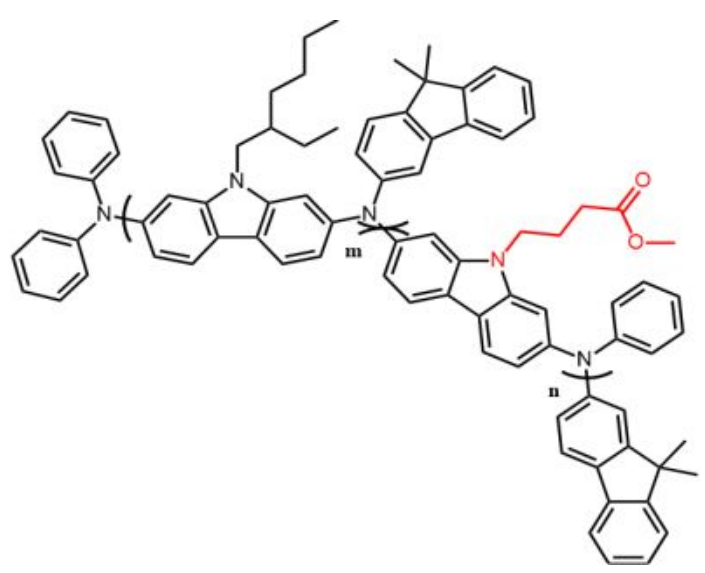

A 50 mL Schlenk flask and stir bar were oven dried. The flask was charged with 2,7-dibromo-9-(2-ethylhexyl)- carbazole (0.679 g, 1.55 mmol), 2-amino-9,9-dimethylfluorene (0.454 g, 2.18 mmol), NaOtBu (0.703 g, 7.31 mmol), 4-(2,7-dibromo-9H-carbazol-9-yl)butanoate (0.221 g, 0.518 mmol), and bis(tri-tert-butylphosphine)palladium(0) (0.05 g, 0.097 mmol) followed by vacuum/N_2 refill cycles (3x). Anhydrous toluene (30 mL) was added with stirring and the reaction was heated to 80 °C for 18 hours. The polymer was end-capped with bromobenzene (0.013 mL, 0.122 mmol) and heated to 90°C for 1 hour. The reaction was cooled to room temperature and was precipitated dropwise into 200 mL cold stirring methanol. The reaction flask was rinsed with 2 mL THF and added dropwise to the methanol. A bright yellow/green precipitate was collected by vacuum filtration and dried overnight in a vacuum oven at 50°C and then dissolved in minimal THF to which diethylammonium diethyldithiocarbamate (0.017 g, 0.075 mmol) was added as a Pd scavenger. This was stirred at reflux for 2 hours. The solution was cooled to room temperature and precipitated into 200 mL stirring methanol and the solids were recovered by vacuum filtration and dried overnight in a vacuum oven at 50°C to achieve a pure light green product (67.9%). Other CzFl-$COOCH_3$ polymers were synthesized via the same method with appropriate molar ratios. ^{1}H NMR (500 MHz, Benzene-d_6) δ 7.91 (d, J = 8.4 Hz, 2H), 7.72 – 7.28 (m, 11H), 7.20 (t, J = 7.1 Hz, 5H), 3.71 – 3.40 (m, 2H), 1.76 (s, 1H), 1.44 – 1.26 (m, 9H), 1.13 – 0.77 (m, 9H), 0.62 (q, J = 11.7, 9.1 Hz, 4H), 0.54 – 0.42 (m, 3H). Thermal analysis (T_g=185℃, T_d=428℃). UV-Vis Absorbance wavelength = 401 nm. Optical band gap energy = 2.84 eV.

FlCz-N

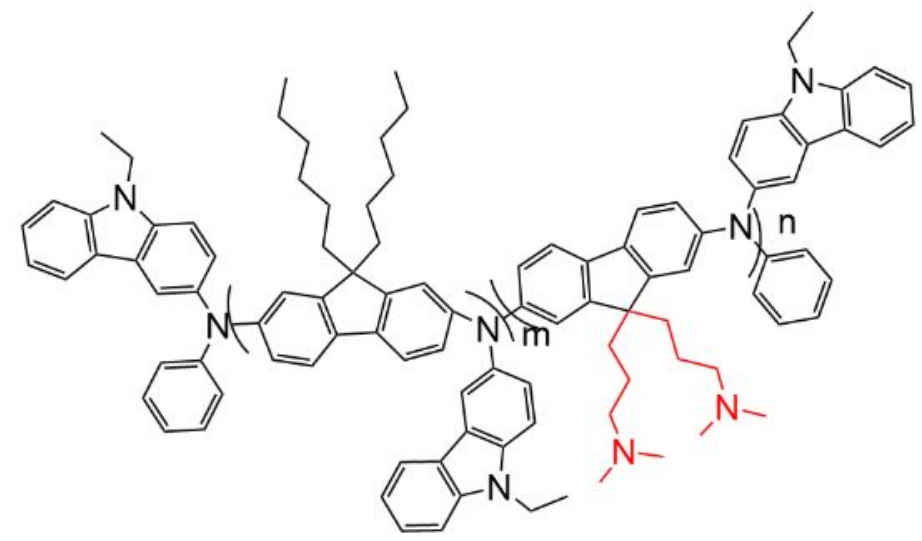

A 100 mL Schlenk flask and a stir bar were oven dried at 105 °C and then charged with 2,7-dibromo-9,9-dihexyl-9H-fluorene (1.62 g, 3.298 mmol), 3-amino-9-ethylcarbazole (0.832 g, 3.96 mmol), 2,7-dibromo dimethylaminopropyl fluorene (0.287 g, 0.58 mmol), and sodium tert-butoxide (1.31 g, 13.69 mmol). The flask was purged and N_2 refilled three times. Bis(tri-tert-butylphosphine)palladium(0) (0.0932 g, 0.182 mmol) was collected from the glovebox and added to the flask with positive nitrogen flow. Anhydrous toluene (50 mL) was added to the flask while stirring via a syringe. The reaction mixture was heated to 80°C for 24 hours at which point bromobenzene (0.0339 mL, 0.229 mmol) was added under positive N_2 flow and allowed to react for 1 hour at 90°C. The solution was then allowed to cool to room temperature while stirring. Once the reaction was cool, 5 mL of THF was added, and the mixture was added dropwise into 300 mL of stirred acidic MeOH for precipitation, followed by 15 min of stirring. The precipitate was collected via vacuum filtration and placed in an oven at 55°C for 1 hour. The product was then dissolved in a minimal amount of chloroform in a vial and charged with diethylammonium diethyldithiocarbamate (0.388 g, 1.74 mmol) to remove any remaining Pd. This solution was heated at 60°C while stirring for 2 hours. The reaction was again cooled down to room temperature and precipitated into MeOH. The product was collected via vacuum filtration and dried in a vacuum oven at 50°C for 24 hours, resulting in a light green powder (84.6%). ^{1}H NMR (500 MHz, Benzene-d_6) δ 8.19 (s, 1H), 7.75 (s, 1H), 7.62 – 7.26 (m, 9H), 6.99 (d, J = 32.6 Hz, 2H), 3.61 (s, 2H), 1.92 (s, 3H), 1.72 (s, 3H), 1.07 (d, J = 58.7 Hz, 17H), 0.85 (s, 4H), 0.77 (d, J = 7.3 Hz, 6H). Thermal analysis (T_g=167°C, T_d=407°C). UV-Vis Absorbance wavelength= 427 nm. Optical band gap energy = 2.80 eV.

FlCz-$COOCH_3$

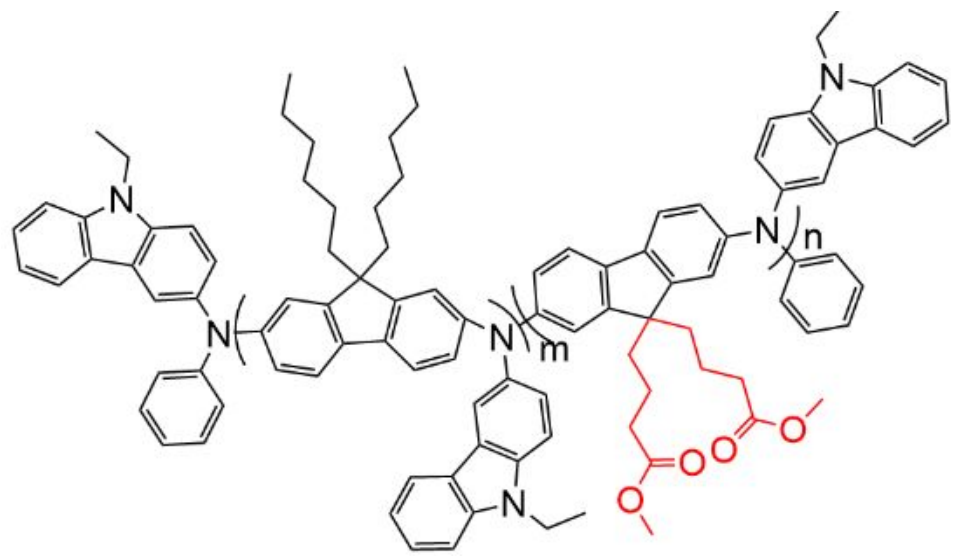

A 100 mL Schlenk flask and a stir bar were oven dried at 105 °C and then charged with 2,7-dibromo-9,9-dihexyl-9H-fluorene (1.62 g, 3.298 mmol), 3-amino-9-ethylcarbazole (0.832 g, 3.96 mmol), dimethyl 4,4'-(2,7-dibromo-9H-fluorene-9,9-diyl)dibutyrate (0.287 g, 0.58 mmol), and sodium tert-butoxide (1.31 g, 13.69 mmol). The flask was purged and N_2 refilled three times. Bis(tri-tert-butylphosphine)palladium(0) (0.0932 g, 0.182 mmol) was collected from the glovebox and added to the flask with positive nitrogen flow. Anhydrous toluene (50 mL) was added to the flask while stirring via a syringe. The reaction mixture was heated to 80°C for 24 hours at which point bromobenzene (0.0339 mL, 0.229 mmol) was added under positive N_2 flow and allowed to react for 1 hour at 90°C. The solution was then allowed to cool to room temperature while stirring. Once the reaction was cool, 5 mL of THF was added, and the mixture was added dropwise into 300 mL of stirred acidic MeOH for precipitation, followed by 15 min of stirring. The precipitate was collected via vacuum filtration and placed in an oven at 55°C for 1 hour. The product was then dissolved in a minimal amount of chloroform in a vial and charged with diethylammonium diethyldithiocarbamate (0.388 g, 1.74 mmol to remove any remaining Pd. This solution was heated at 60°C while stirring for 2 hours. The reaction was again cooled down to room temperature and precipitated into MeOH. The product was collected via vacuum filtration and dried in a vacuum oven at 50°C for 24 hours, resulting in a light green powder (92.5%). ^{1}H NMR (500 MHz, Benzene-d6) δ 8.30 – 8.18 (m, 1H), 7.82 (d, J = 7.7 Hz, 1H), 7.75 – 7.51 (m, 5H), 7.51 – 7.28 (m, 4H), 7.12 – 6.98 (m, 2H), 3.77 – 3.62 (m, 2H), 1.99 – 1.62 (m, 5H), 1.20 (p, J = 7.0 Hz, 4H), 1.14 – 0.98 (m, 13H), 0.92 (dd, J = 8.6, 5.5 Hz, 4H), 0.89 – 0.75 (m, J = 7.9, 7.4 Hz, 8H). Thermal analysis (T_g=130°C, T_d=412°C). UV-Vis Absorbance wavelength = 422 nm. Optical band gap energy = 2.77 eV.

CzAn-N

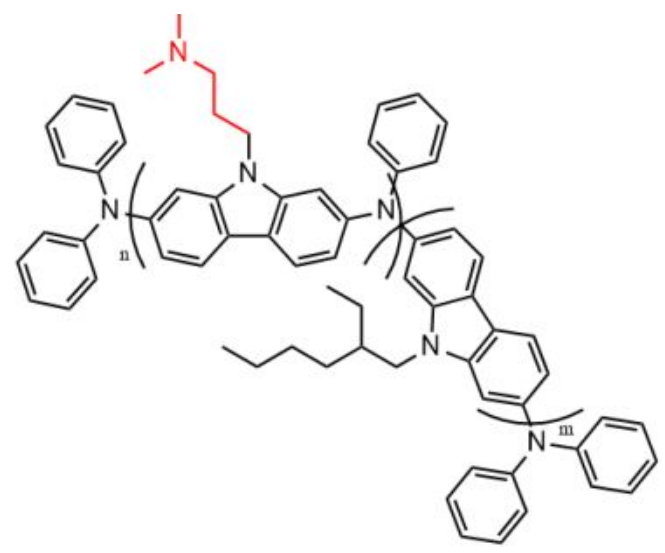

A 100 mL Schlenk flask and a stir bar were oven dried at 105 °C and then charged with 2,7-dibromo-9-(2-(ethylhexyl)-9H-carbazole (1.2 g, 2.74 mmol), 2,7-dibromo-9-(3-(dimethylamino)propyl)-carbazole (0.199 g, 0.484 mmol), and sodium tert-butoxide (0.9309 g, 9.68 mmol). The flask was then purged and N_2 refilled three times. Bis(tri-tert-butylphosphine)palladium(0) (0.066 g, 0.129 mmol) was collected from the glovebox and added to the flask with positive nitrogen flow. Anhydrous toluene (25 mL) and aniline (0.3007 mL, 3.29 mmol) were added to the flask while stirring via a syringe. The reaction mixture was heated to 80°C for 24 hours at which point bromobenzene (0.0168 mL, 0.161 mmol) was added under positive N_2 flow and allowed to react for 1 hour at 90°C. The solution was then allowed to cool to room temperature while stirring. Once the reaction was cool, 5 mL of THF was added and the mixture was added dropwise into 300 mL of stirred acidic MeOH for precipitation, followed by 15 min of stirring. The precipitate was collected via vacuum filtration and placed in an oven at 55°C for 1 hour. The product was then dissolved in a minimal amount of chloroform in a vial and charged with diethylammonium diethyldithiocarbamate (0.323 g, 1.45 mmol) to remove any remaining Pd. This solution was heated at 60°C while stirring for 2 hours. The reaction was again cooled down to room temperature and precipitated into MeOH. The product was collected via vacuum filtration and dried in a vacuum oven at 50°C for 24 hours, resulting in a light green powder (86.9%). ^{1}H NMR (500 MHz, Benzene-d_6) δ 7.79 (s, 2H), 7.35 (s, 7H), 6.83 (s, 1H), 3.46 (s, 2H), 1.72 (s, 2H), 0.93 (s, 7H), 0.64 (s, 3H), 0.51 – 0.43 (m, 2H). Thermal analysis (T_g=137°C, T_d=417°C). UV-Vis Absorbance wavelength = 406 nm. Optical band gap energy = 2.93 eV.

CzAn-$COOCH_3$

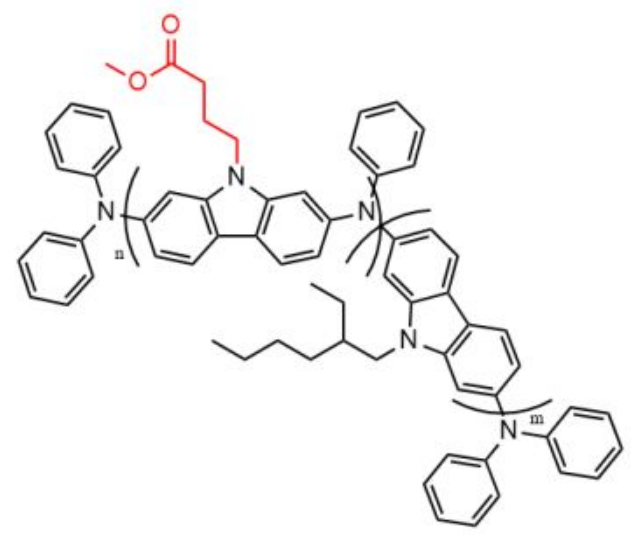

A 100 mL Schlenk flask and a stir bar were oven dried at 105 °C and then charged with 2,7-dibromo-9-(2-(ethylhexyl)-9H-carbazole (2.52 g, 5.75 mmol), methyl 4-(2,7-dibromo-9H-carbazol-9-yl)butanoate (0.416 g, 1.02 mmol), sodium tert-butoxide (2.296 g, 23.9 mmol), and bis(tri-tert-butylphosphine)palladium(0) (0.163 g, 0.318 mmol). The flask was then purged and refilled with N_2 three times. Anhydrous toluene (50 mL) and aniline (0.630 mL, 6.90 mmol) were added to the flask while stirring via a syringe. The reaction mixture was heated to 80°C for 24 hours at which point bromobenzene (0.042 mL, 0.063 mmol) was added under positive N_2 flow and allowed to react for 1 hour at 90°C. The solution was then allowed to cool to room temperature while stirring. Once the reaction was cool, 5 mL of THF was added, and the mixture was added dropwise into 300 mL of stirred acidic MeOH for precipitation, followed by 15 min of stirring. The precipitate was collected via vacuum filtration and placed in an oven at 55°C for 1 hour. The product was then dissolved in a minimal amount of chloroform in a vial and charged with diethylammonium diethyldithiocarbamate (0.212 g, 0.954 mmol) to remove any remaining Pd. This solution was heated at 60°C while stirring for 2 hours. The reaction was again cooled down to room temperature and precipitated into MeOH. The product was collected via vacuum filtration and dried in a vacuum oven at 50°C for 24 hours, resulting in a light green powder (63.8%). ^{1}H NMR (500 MHz, Benzene-d_6) δ 7.85 (s, 2H), 7.46 – 7.23 (m, 7H), 6.89 (s, 1H), 3.51 (s, 2H), 1.74 (s, 1H), 0.95 (d, J = 43.2 Hz, 8H), 0.70 (s, 3H), 0.54 (s, 3H). Thermal analysis (T_g=145°C, T_d=443°C). UV-Vis Absorbance wavelength = 409 nm. Optical band gap energy = 2.90 eV.

FlAnS-N

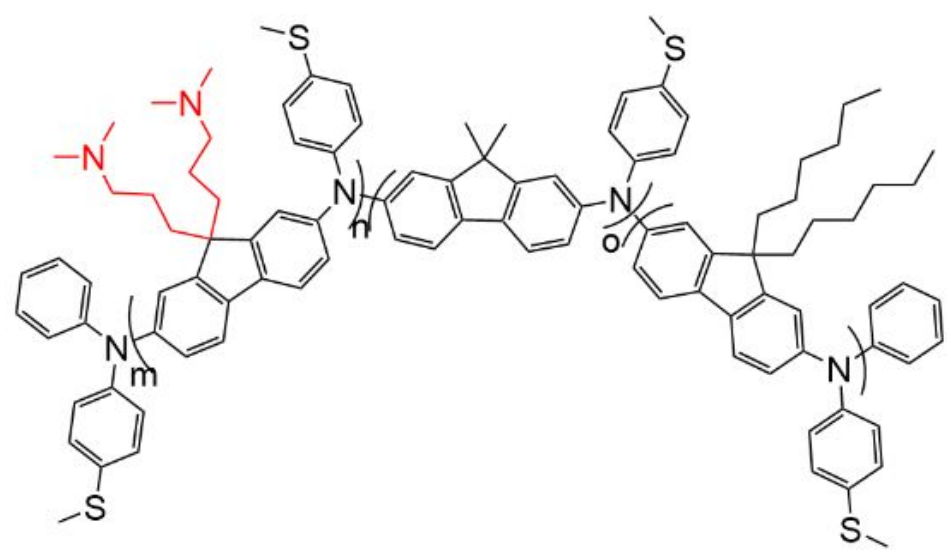

A 100 mL Schlenk flask and a stir bar were oven dried at 105 °C and then charged with 2,7-dibromo-9,9-dihexyl-9H-fluorene (1.88 g, 3.811 mmol), dimethyldibromofluorene (0.336 g, 0.953 mmol), 2,7-dibromo dimethylaminopropyl fluorene (0.416 g, 0.841 mmol), and sodium tert-butoxide (1.902 g, 19.79 mmol). The flask was purged and N_2 was refilled three times. Bis(tri-tert-butylphosphine)palladium(0) (0.135 g, 0.263 mmol) was collected from the glovebox and added to the flask with positive nitrogen flow. Anhydrous toluene (50 mL) and 4-methylmercaptoaniline (0.711 mL, 5.718 mmol) were added to the flask while stirring via a syringe. The reaction mixture was heated to 80°C for 24 hours at which point bromobenzene (0.0346 mL, 0.331 mmol) was added under positive N_2 flow and allowed to react for 1 hour at 90°C. The solution was then allowed to cool to room temperature while stirring. Once the reaction was cool, 5 mL of THF was added, and the mixture was added dropwise into 300 mL of stirred acidic MeOH for precipitation, followed by 15 min of stirring. The precipitate was collected via vacuum filtration and placed in an oven at 55°C for 1 hour. The product was then dissolved in a minimal amount of chloroform in a vial and charged with diethylammonium diethyldithiocarbamate (0.561 g, 2.52 mmol) to remove any remaining Pd. This solution was heated at 60°C while stirring for 2 hours. The reaction was again cooled down to room temperature and precipitated into MeOH. The product was collected via vacuum filtration and dried in a vacuum oven at 50°C for 24 hours, resulting in a light green powder (84.9%).^{1}H NMR (500 MHz, Benzene-d_6) δ 7.48 – 7.30 (m, 4H), 7.27 –7.12 (m, 6H), 2.05 – 1.85 (m, 6H), 1.80 (s, 1H), 1.70 (s, 2H), 1.09 (d, J = 59.6 Hz, 11H), 0.90 (q, J = 7.4, 6.7 Hz, 3H), 0.83 (d, J = 7.2 Hz, 4H). Thermal analysis (T_g=153°C, T_{d1}=401 °C). Photoluminescence wavelength = 433 nm. UV-Vis Absorbance wavelength= 411 nm. Optical band gap energy = 2.90 eV.

FlAnS-$COOCH_3$

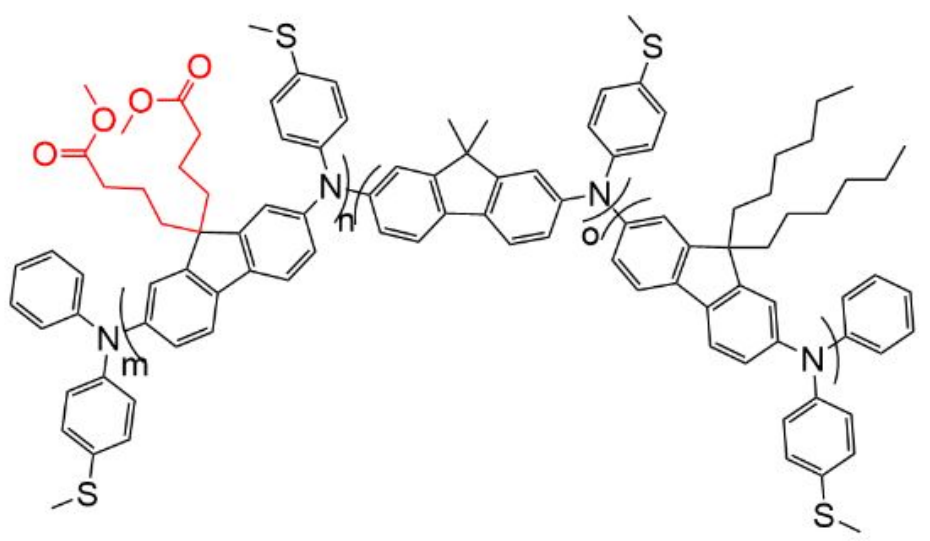

A 100 mL Schlenk flask and a stir bar were oven dried at 105 °C and then charged with 2,7-dibromo-9,9-dihexyl-9H-fluorene (2.01 g, 4.09 mmol), dimethyldibromofluorene (0.336 g, 0.951 mmol), dimethyl 4,4'-(2,7-dibromo-9H-fluorene-9,9-diyl)dibutyrate (0.278 g, 0.560 mmol), and sodium tert-butoxide (1.90 g, 19.77 mmol). The flask was then purged and refilled with N_2 three times. Bis(tri-tert-butylphosphine)palladium(0) (0.135 g, 0.263 mmol) was collected from the glovebox and added to the flask with positive nitrogen flow was added under a positive nitrogen flow. Anhydrous toluene (50 mL) and 4-methylmercaptoaniline (0.711 mL, 5.71 mmol) were added to the flask while stirring via a syringe. The reaction mixture was heated to 80℃ for 24 hours at which point bromobenzene (0.035 mL, 0.330 mmol) was added under positive N_2 flow and allowed to react for 1 hour at 90℃. The solution was then allowed to cool to room temperature while stirring. Once the reaction was cool, 5 mL of THF was added, and the mixture was added dropwise into 300 mL of stirred acidic MeOH for precipitation, followed by 15 min of stirring. The precipitate was collected via vacuum filtration and placed in an oven at 55℃ for 1 hour. The product was then dissolved in a minimal amount of chloroform in a vial and charged with diethylammonium diethyldithiocarbamate (0.560 g, 2.52 mmol) to remove any remaining Pd. This solution was heated at 60℃ while stirring for 2 hours. The reaction was again cooled down to room temperature and precipitated into MeOH. The product was collected via vacuum filtration and dried in a vacuum oven at 50℃ for 24 hours, resulting in a light green powder (82.8%). ^{1}H NMR (500 MHz, Benzene-d_6) δ 7.64 – 7.36 (m, 4H), 7.36 – 7.20 (m, 6H), 2.10 (s, 1H), 2.04 (d, J = 11.0 Hz, 3H), 1.89 (dt, J = 17.1, 6.9 Hz, 1H), 1.82 – 1.55 (m, 3H), 1.46 – 1.28 (m, 1H), 1.22 (dd, J = 15.2, 8.1 Hz, 5H), 1.15 – 1.03 (m, 7H), 1.03 – 0.91 (m, 3H), 0.88 (dtd, J = 7.4, 4.4, 2.3 Hz, 5H). Thermal analysis (T_g=135℃, T_d=454℃). UV-Vis Absorbance wavelength = 412 nm. Optical band gap energy = 2.87 eV.

S3. ^{1}H NMR

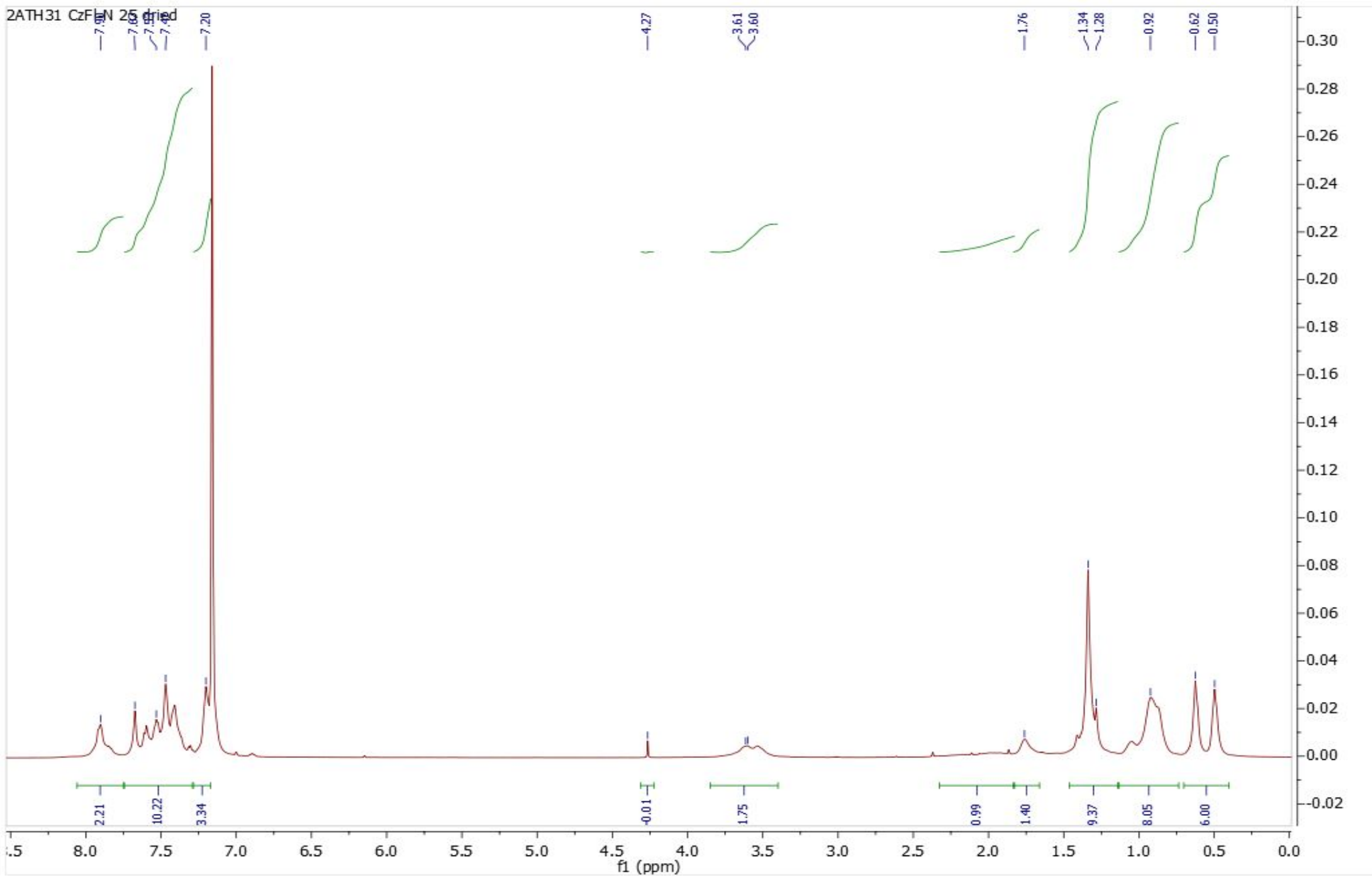

Figure S1. ^{1}H NMR spectrum of CzFl-N

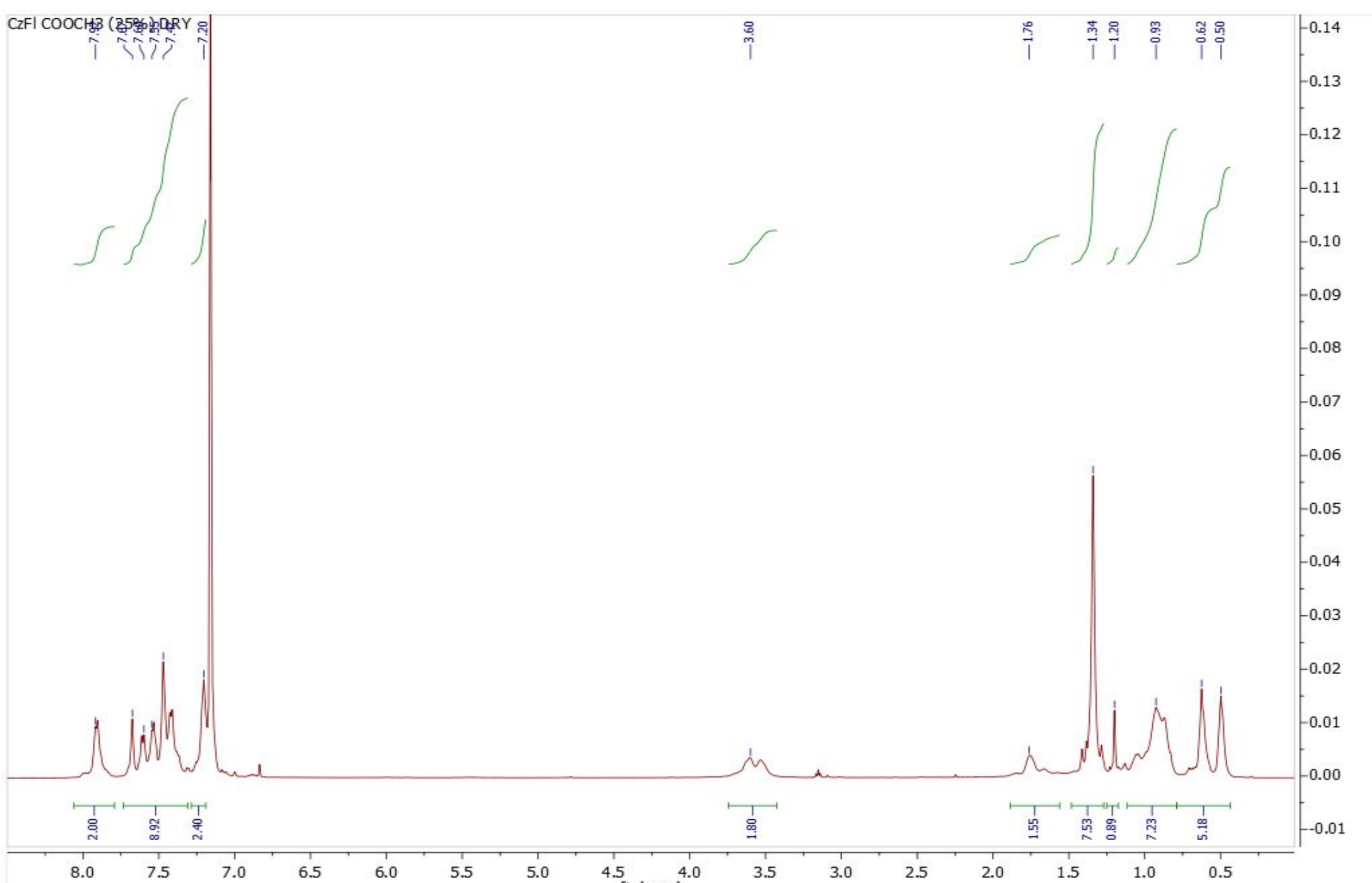

Figure S2. ^{1}H NMR spectrum of CzFl-$COOCH_3$

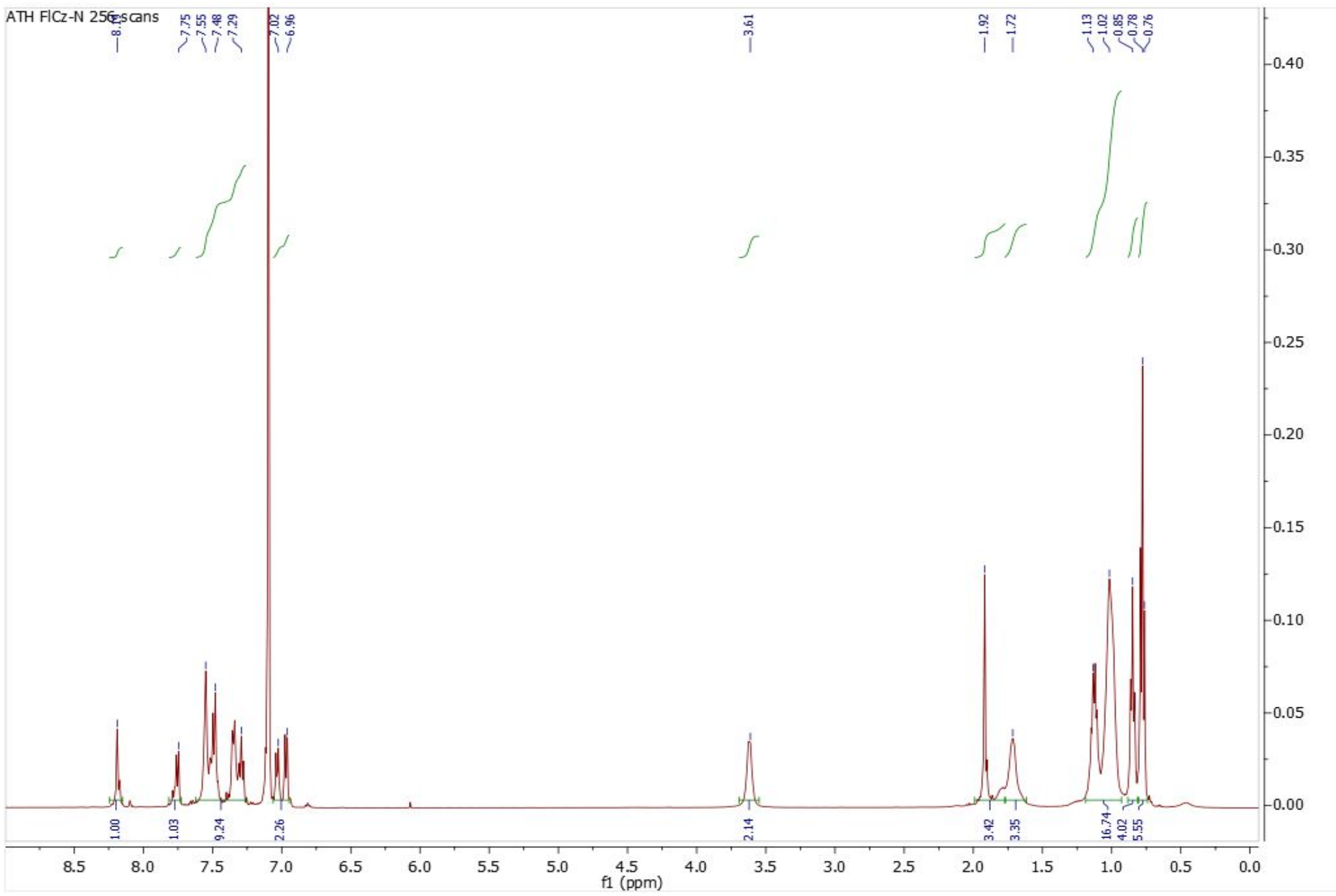

Figure S3. ^{1}H NMR spectrum of FlCz-N

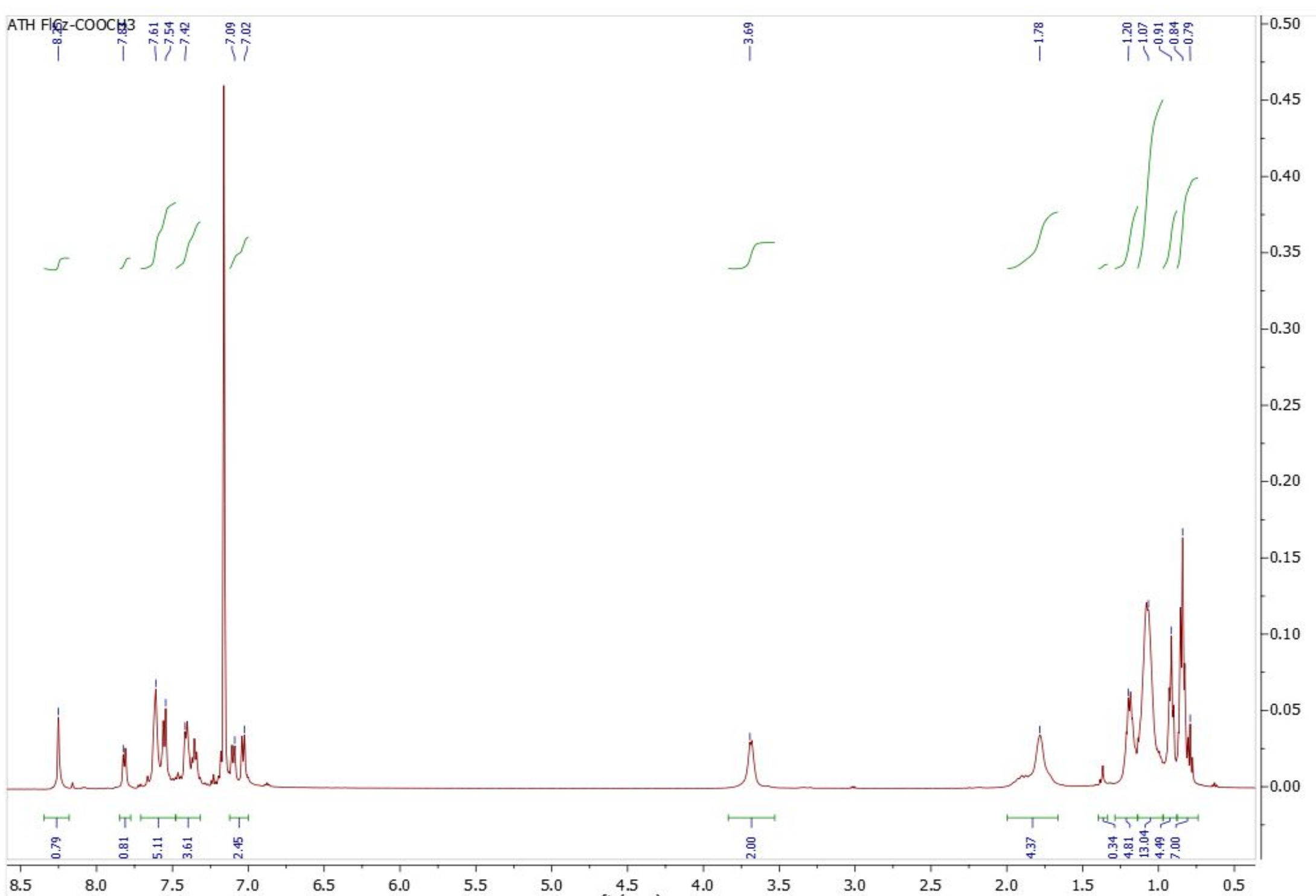

Figure S4. ^{1}H NMR spectrum of FlCz-$COOCH_3$

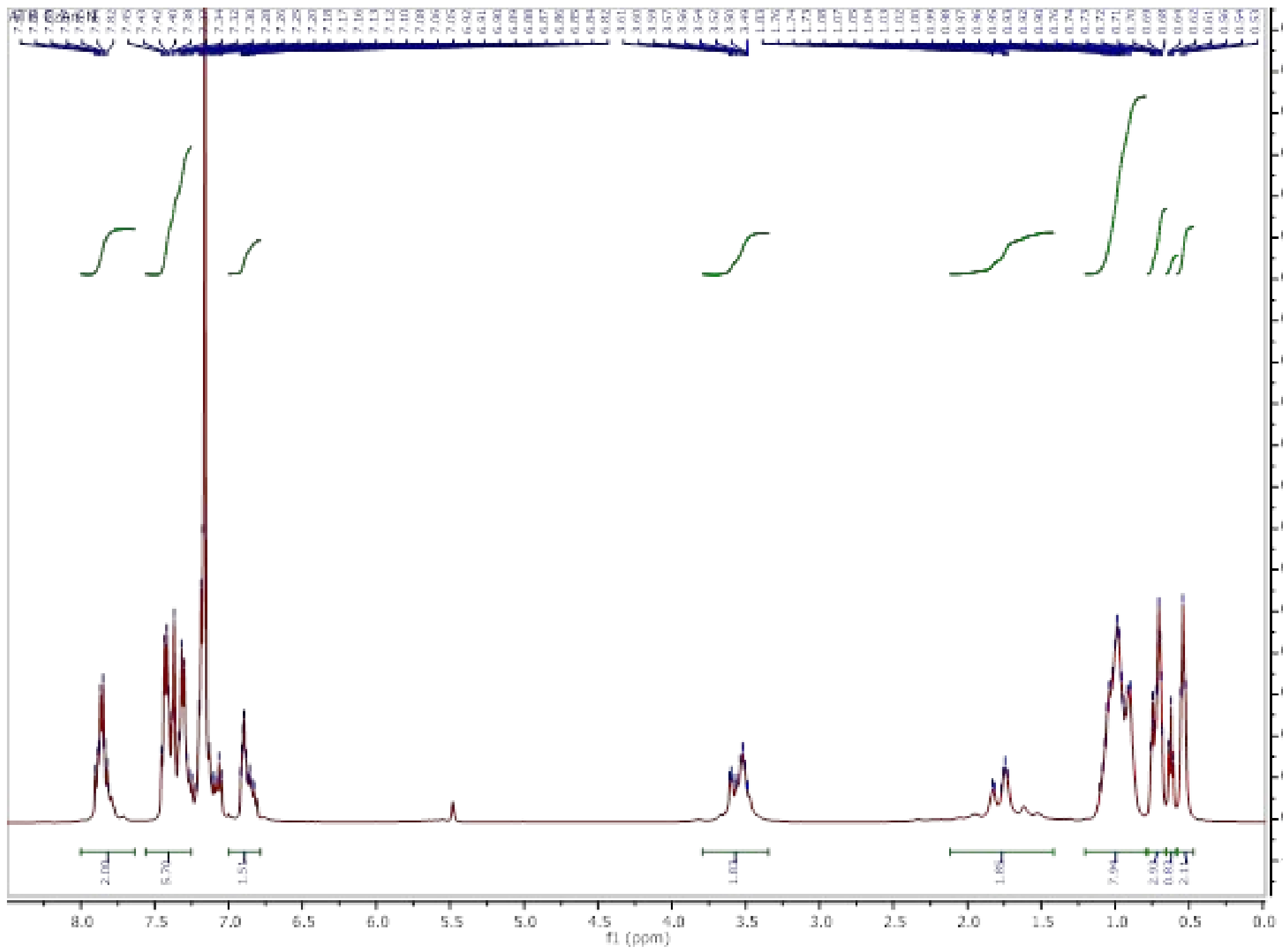

Figure S5. ^{1}H NMR spectrum of CzAn-N

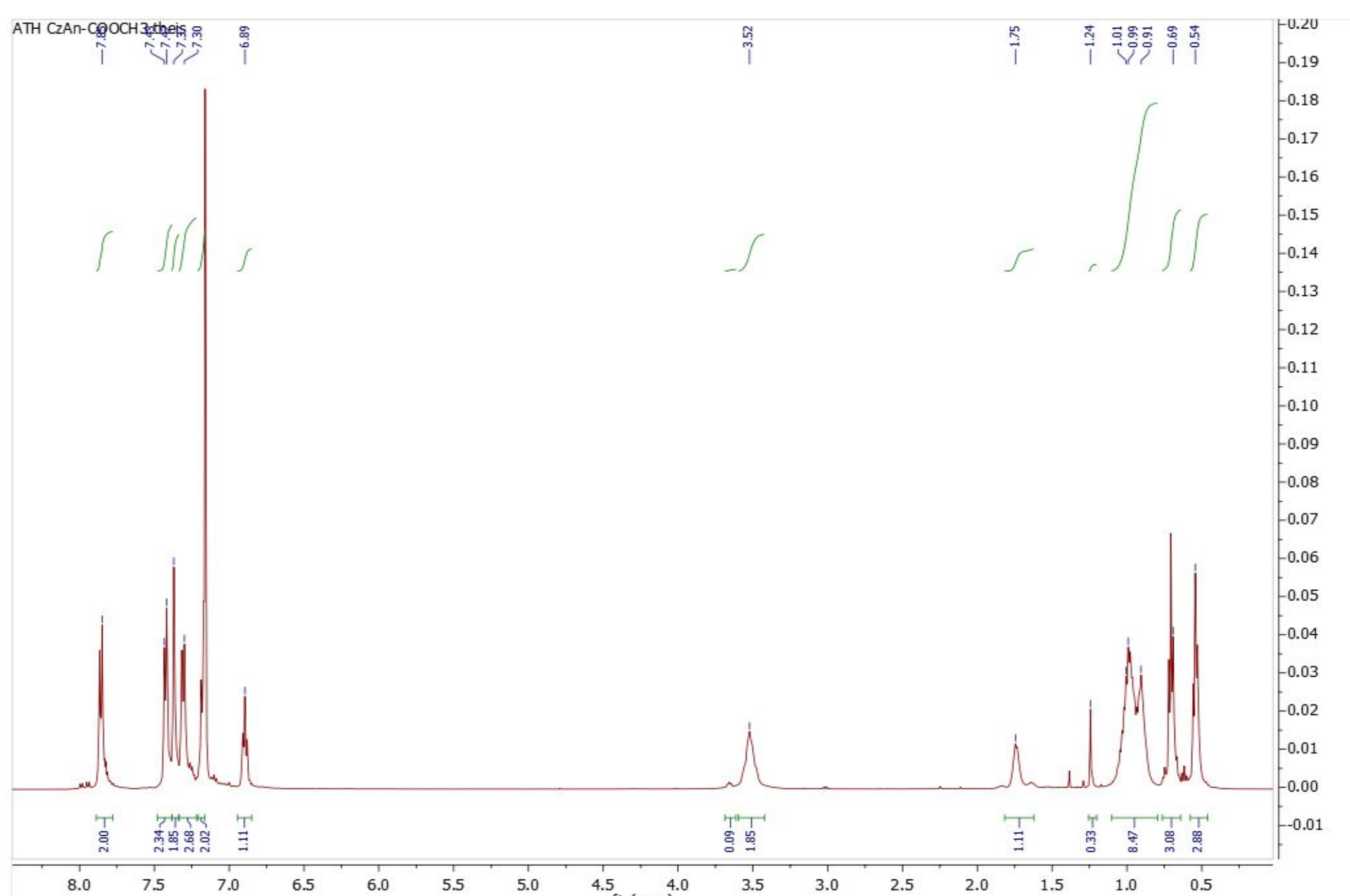

Figure S6. ^{1}H NMR of spectrum CzAn-$COOCH_3$

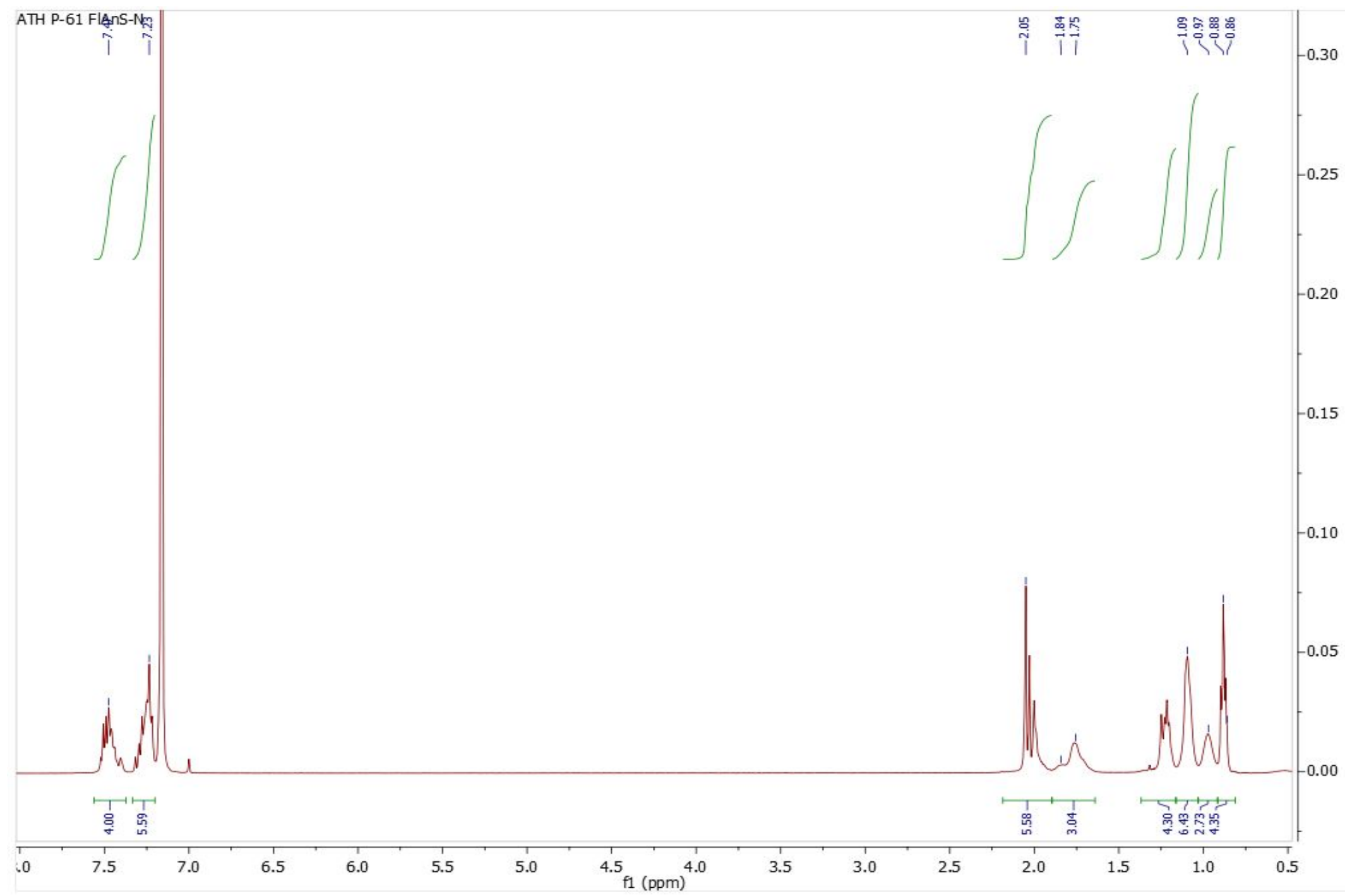

Figure S7. ^{1}H NMR spectrum of FlAnS-N

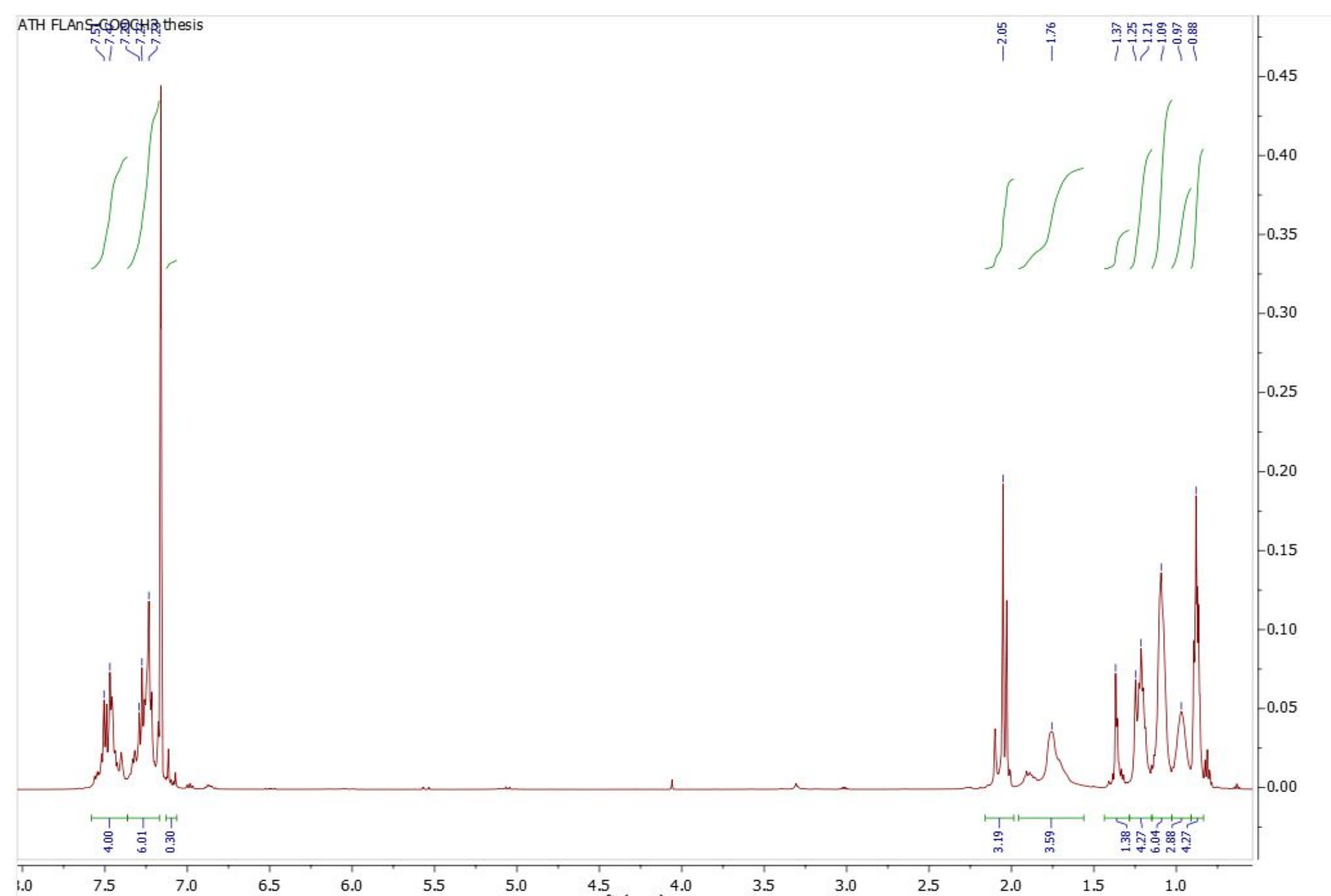

Figure S8. ^{1}H NMR spectrum of FlAnS-$COOCH_3$

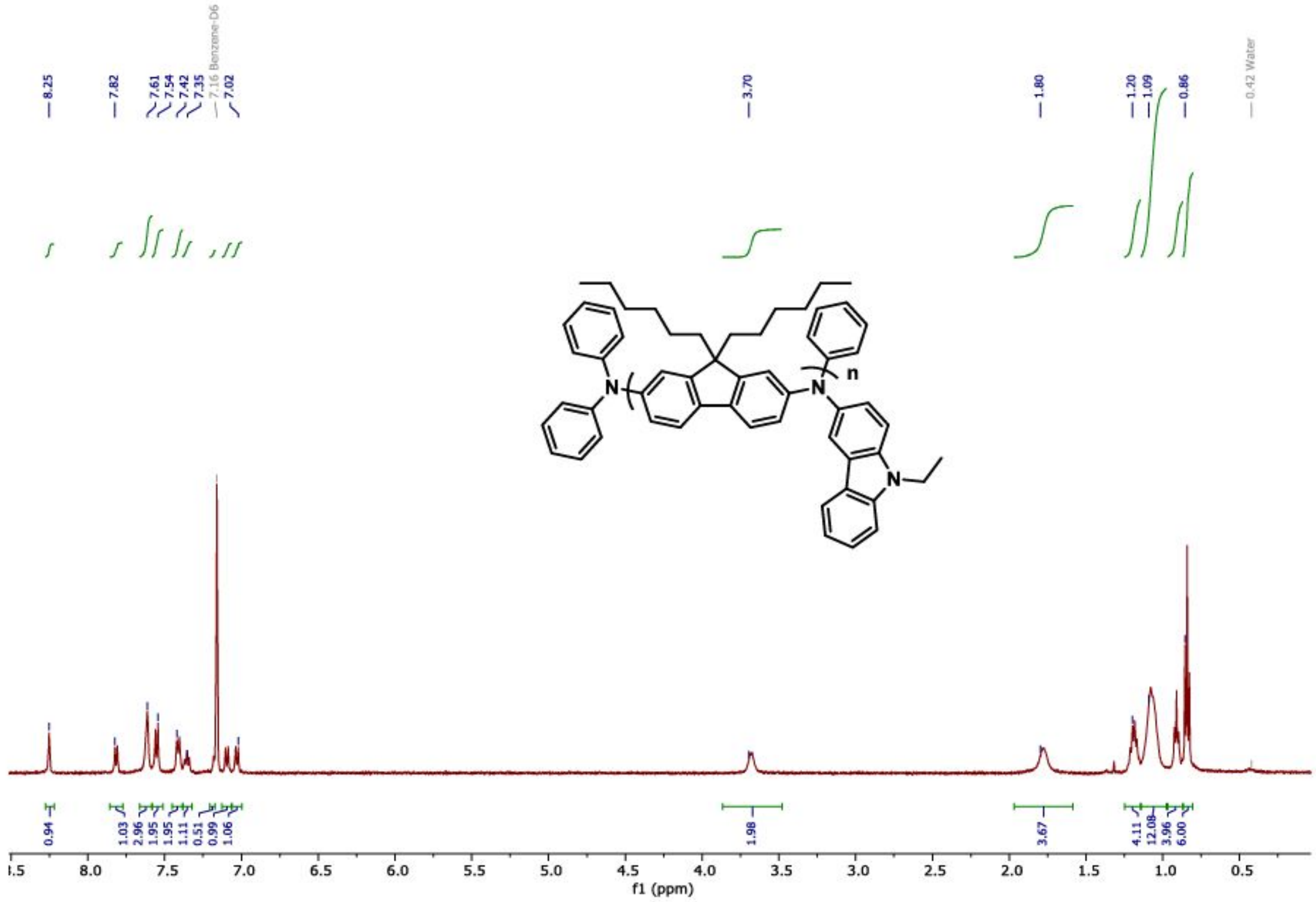

Figure S9. ^{1}H NMR spectrum of FlCz

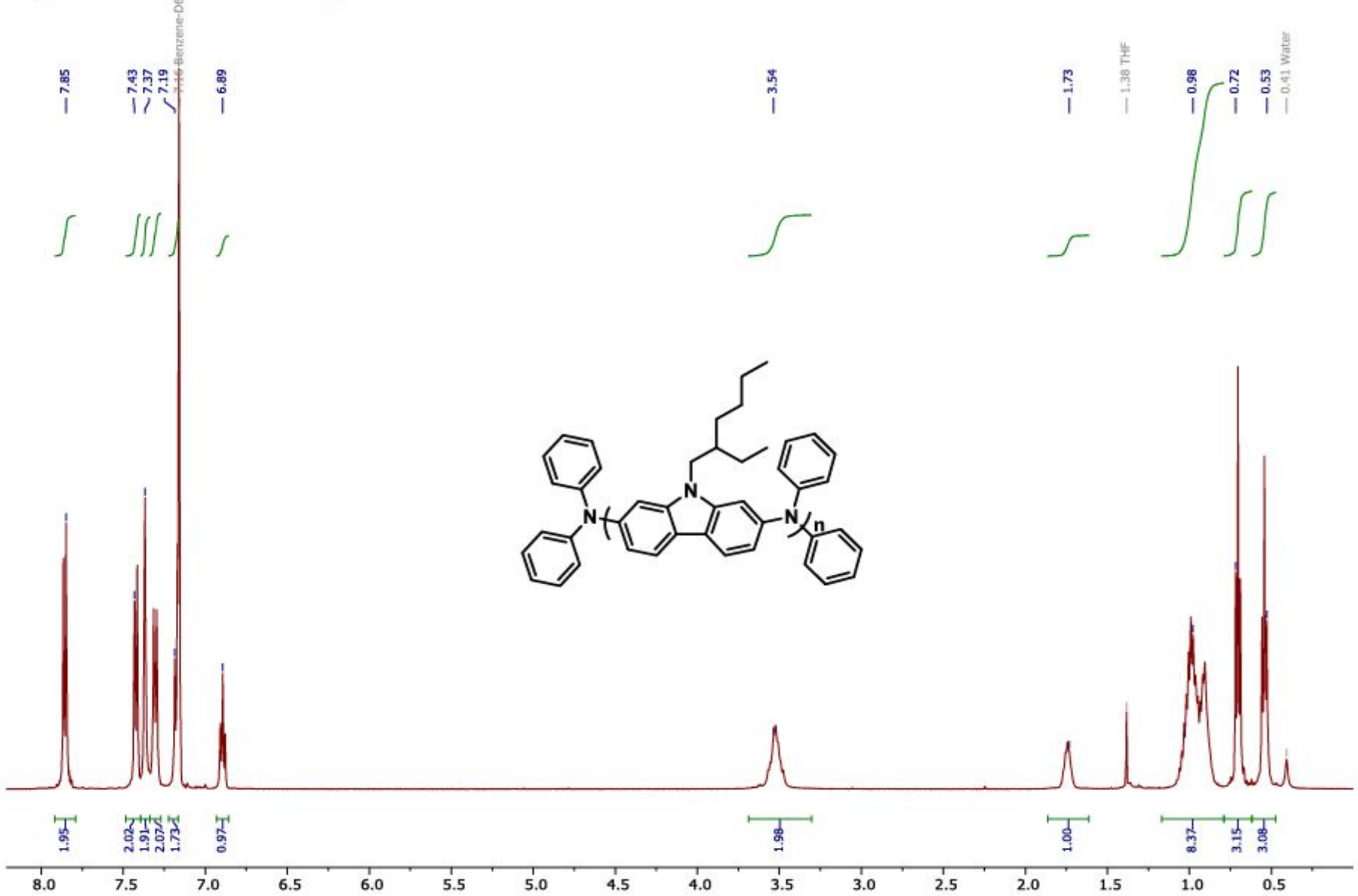

Figure S10. ^{1}H NMR spectrum of CzAn

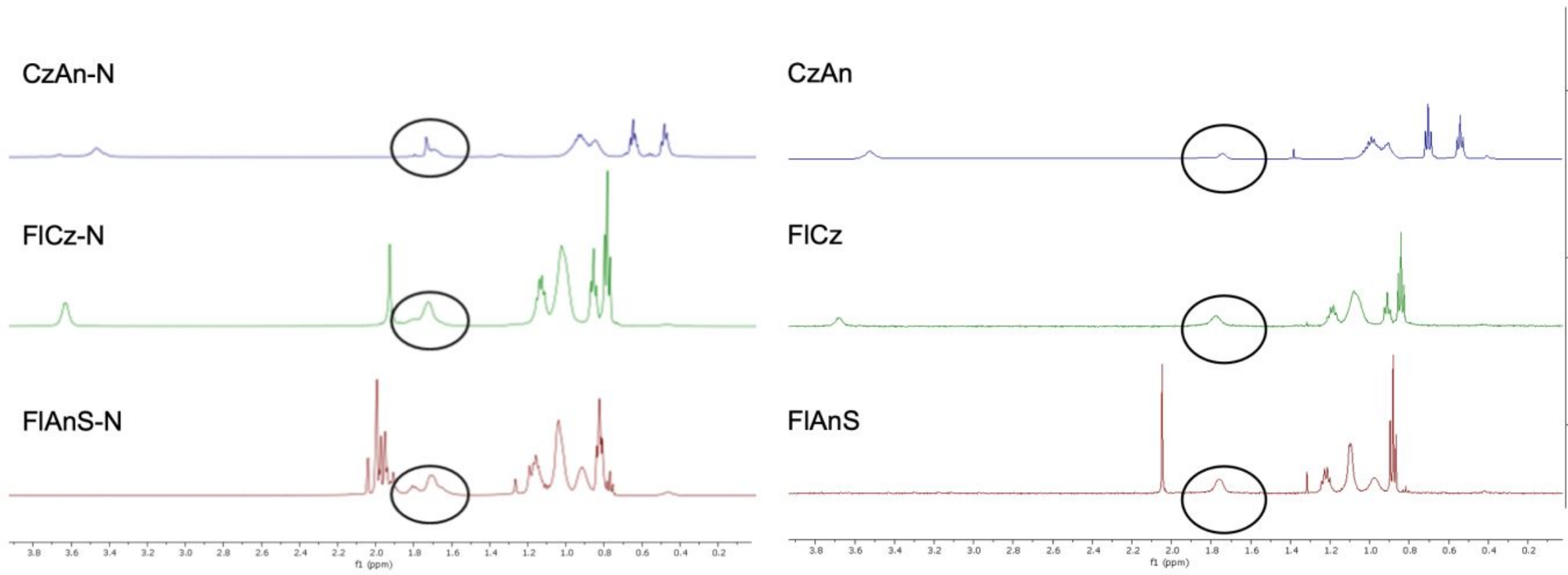

Figure S11. ^{1}H NMR of CzAn-N, FlCz-N, and FlAnS-N compared to the non-functionalized parent polymers. Circled peaks represent the characteristic Cz-N monomer peaks.

S4. TGA

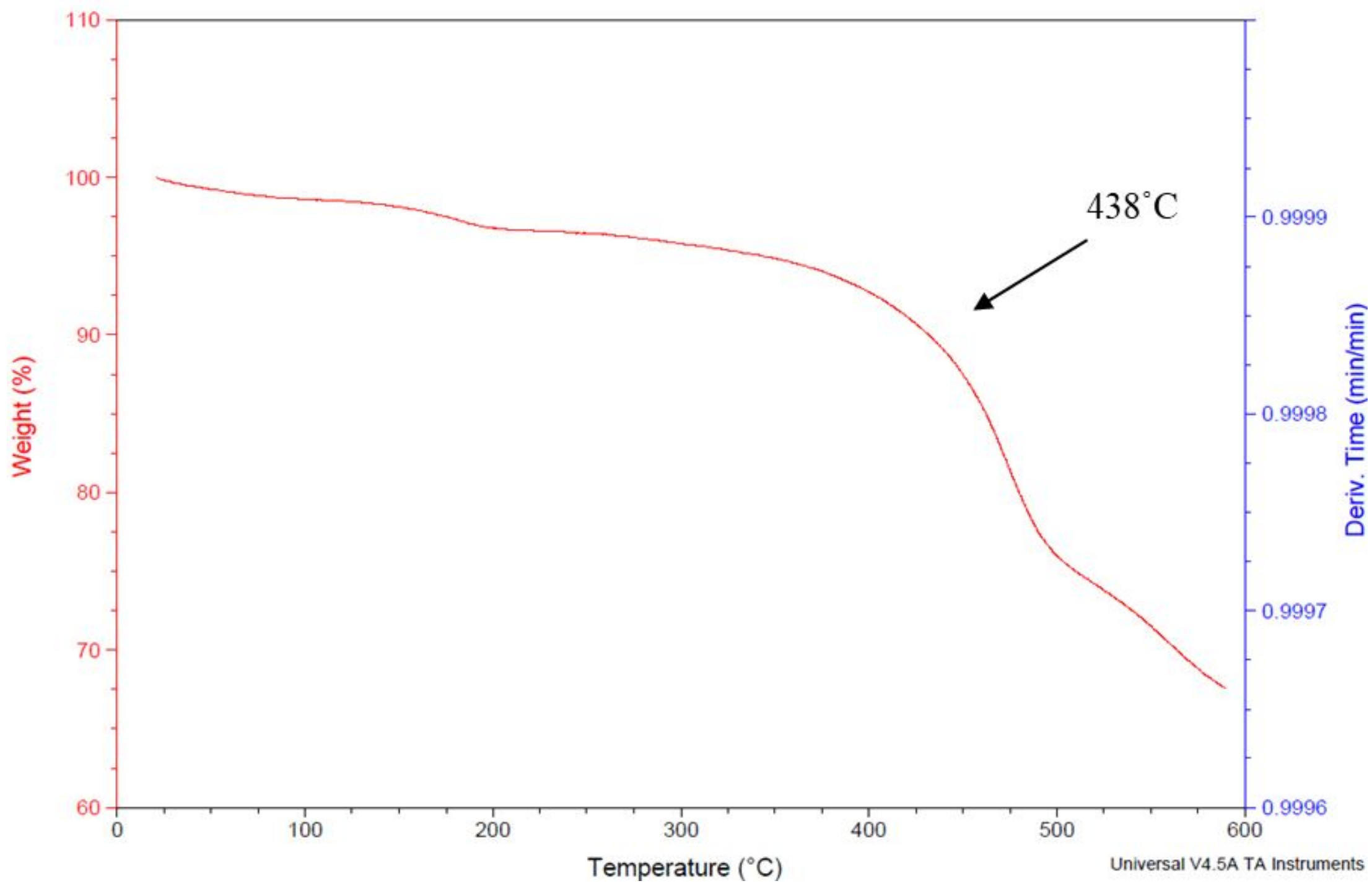

Figure S12. TGA spectrum of CzFl-N

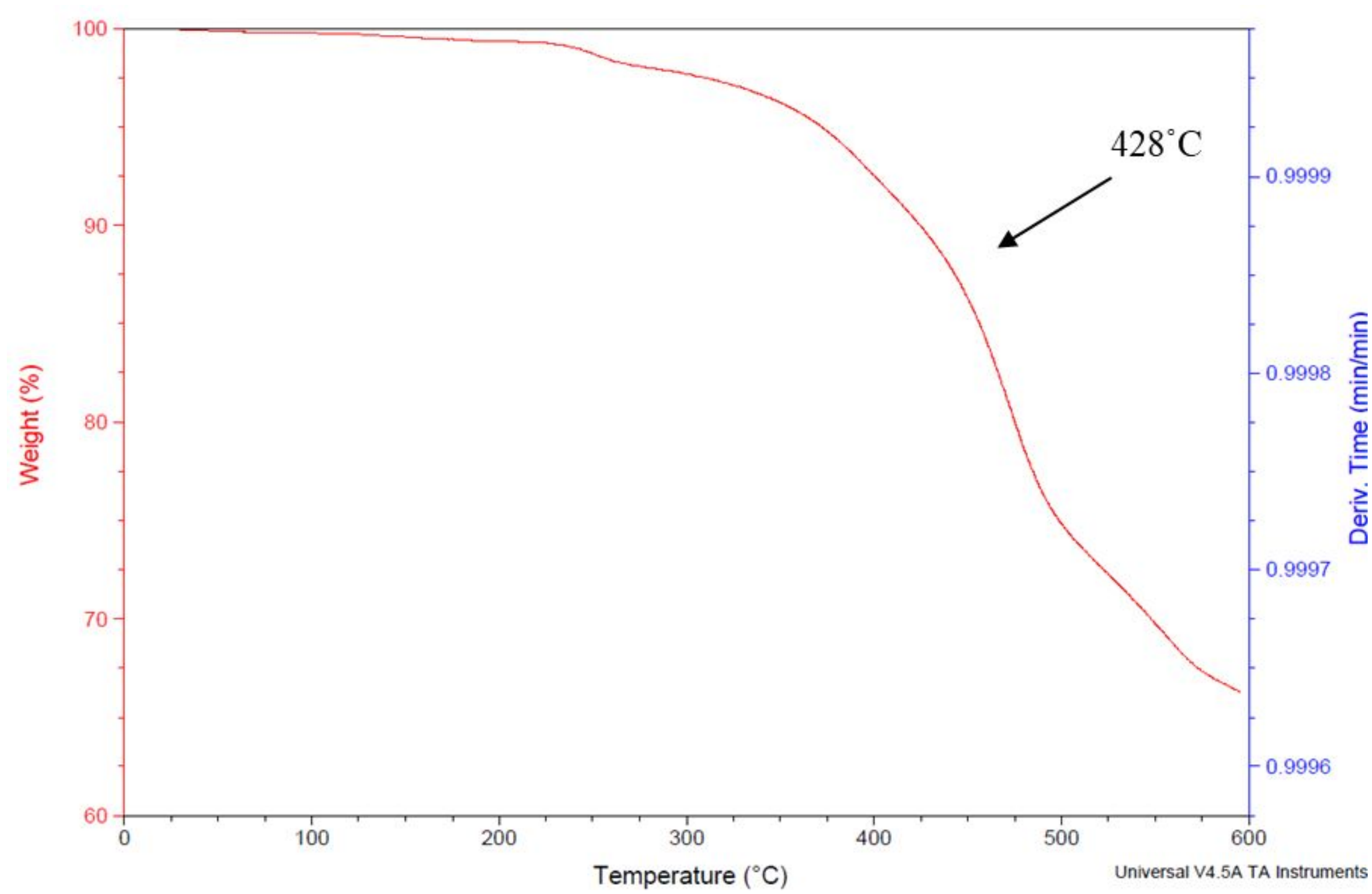

Figure S13.TGA spectrum of CzFl-$COOCH_3$

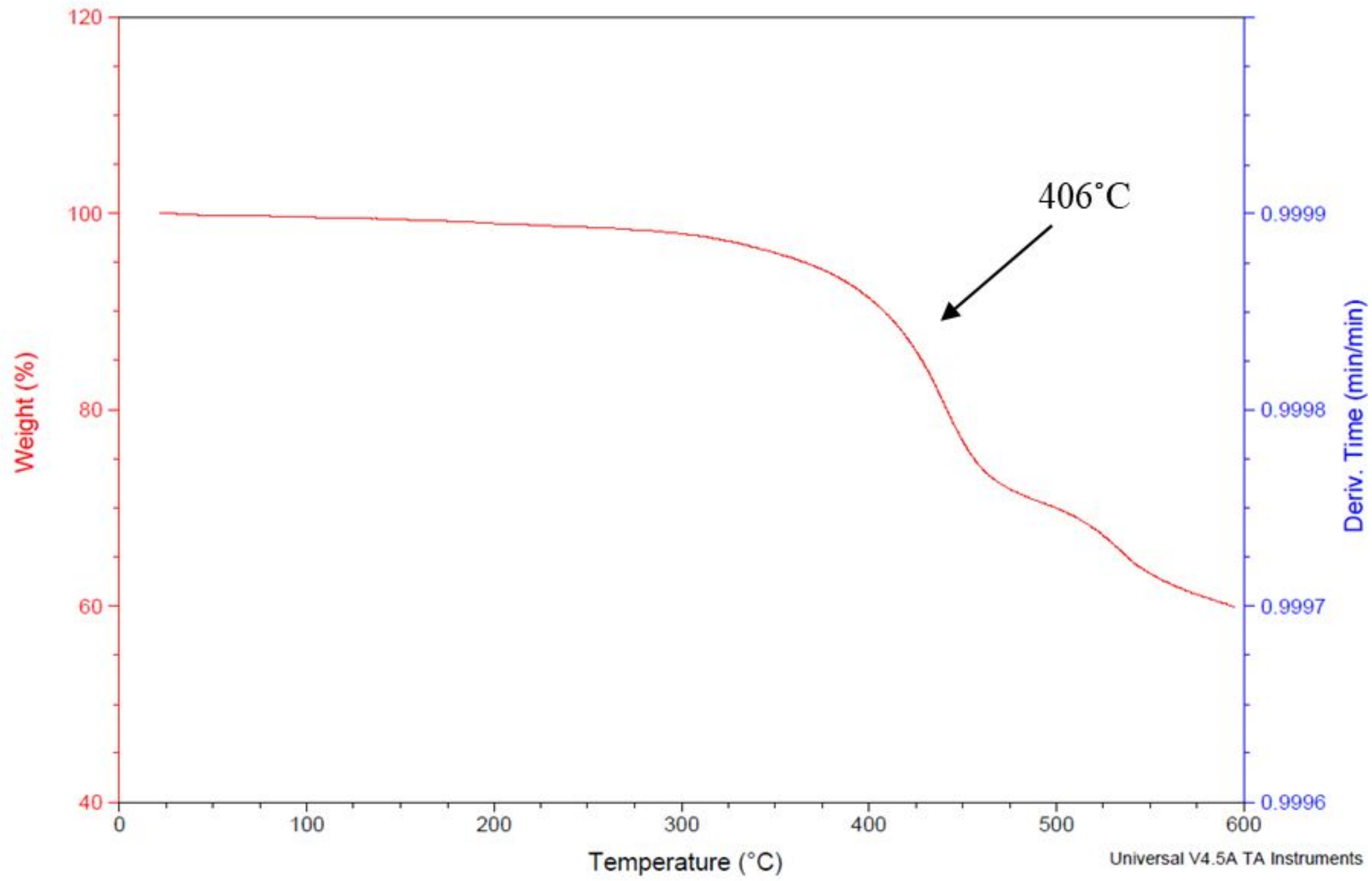

Figure S14. TGA spectrum of FlCz-N

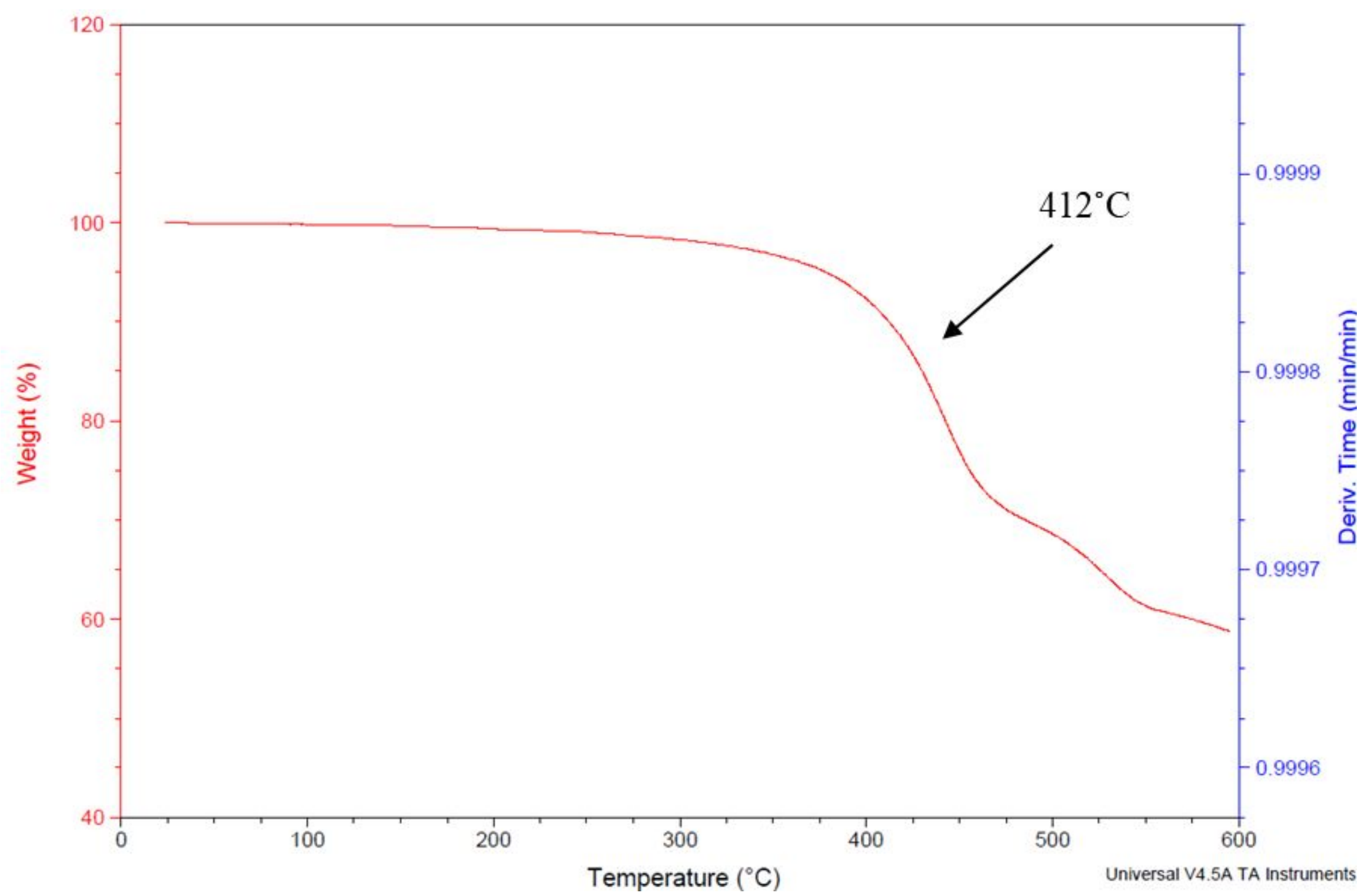

Figure S15. TGA spectrum of FlCz-$COOCH_3$

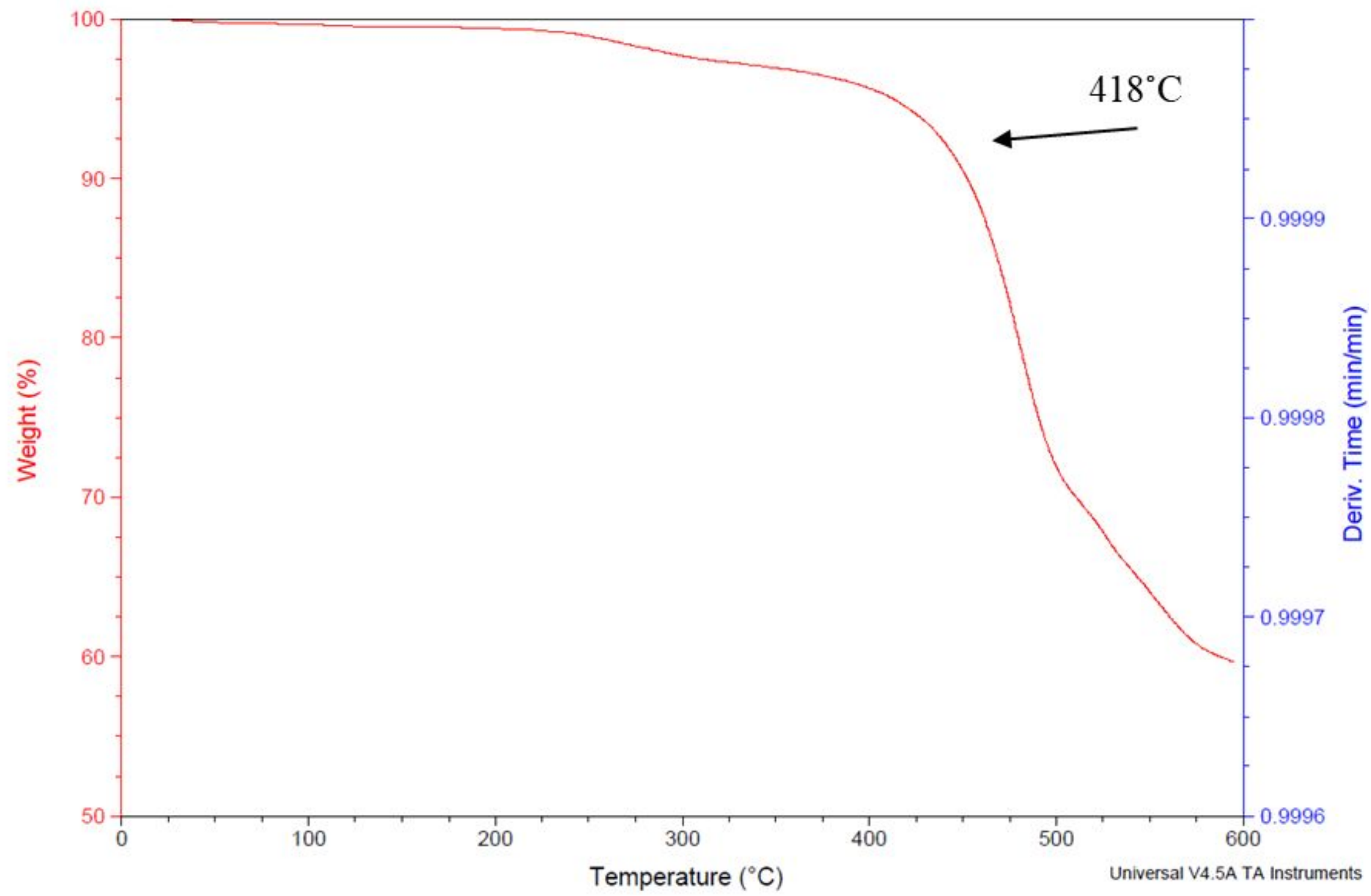

Figure S16. TGA spectrum of CzAn-N

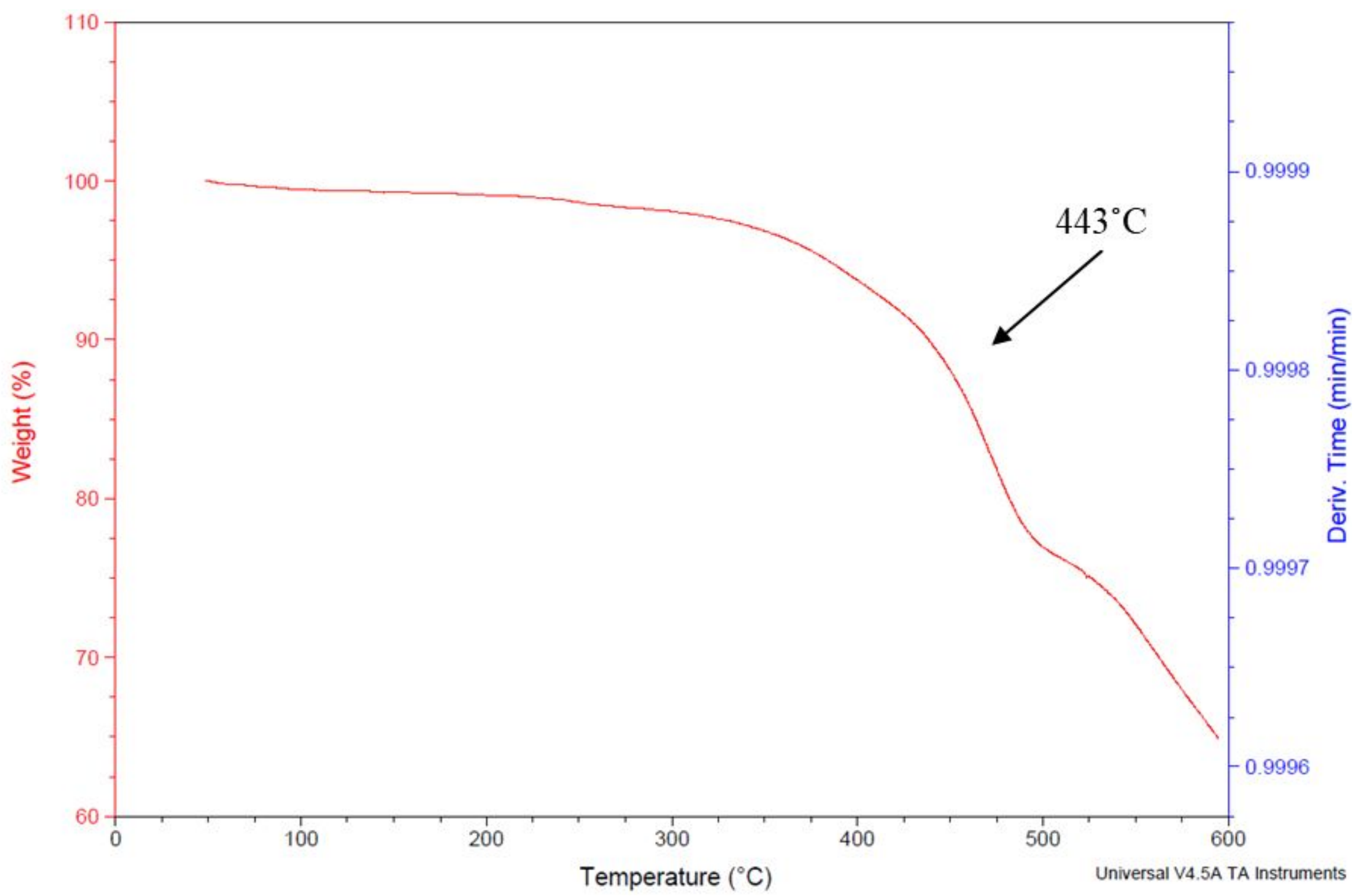

Figure S17. TGA Spectrum of CzAn-$COOCH_3$

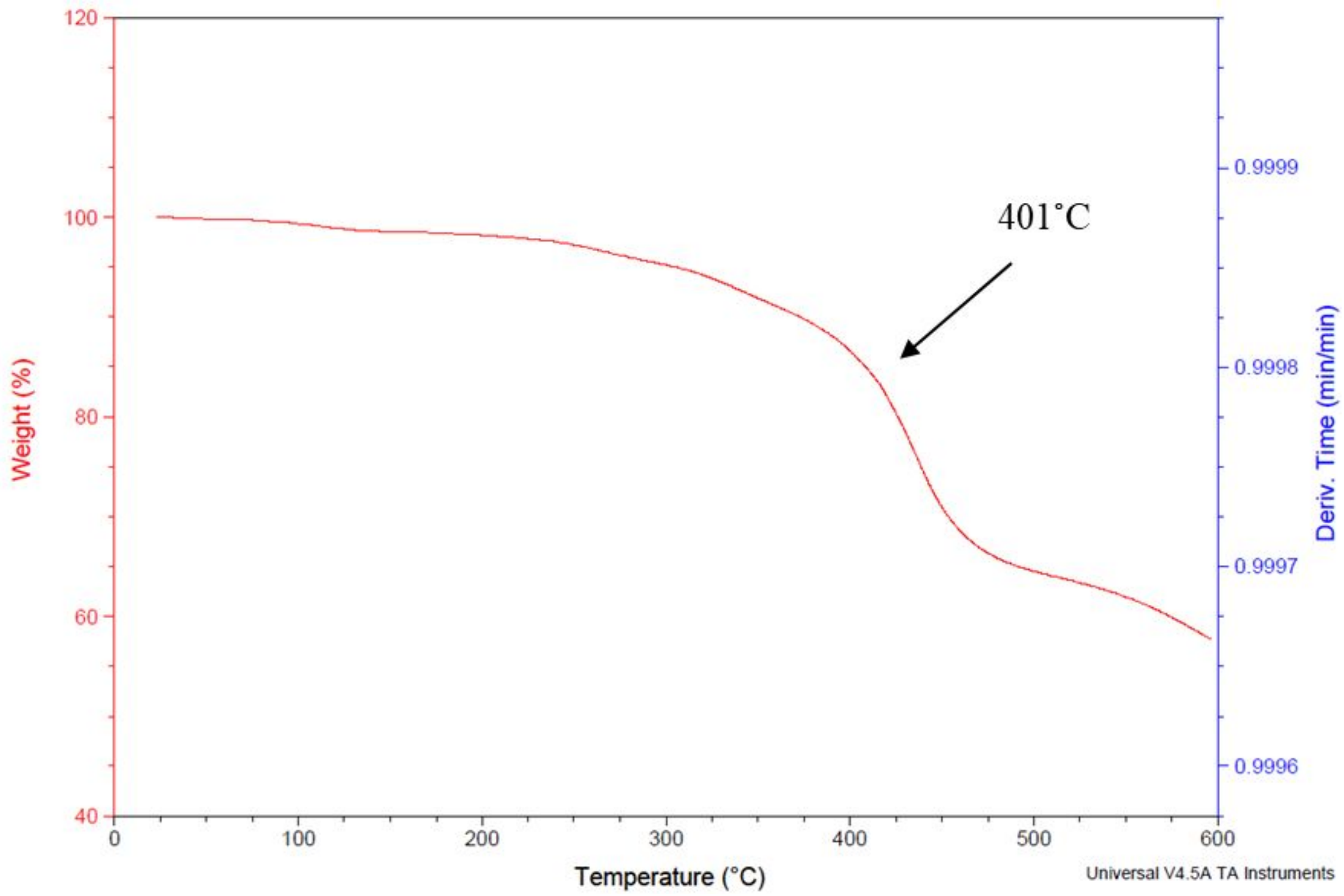

Figure S18. TGA spectrum of FlAnS-N

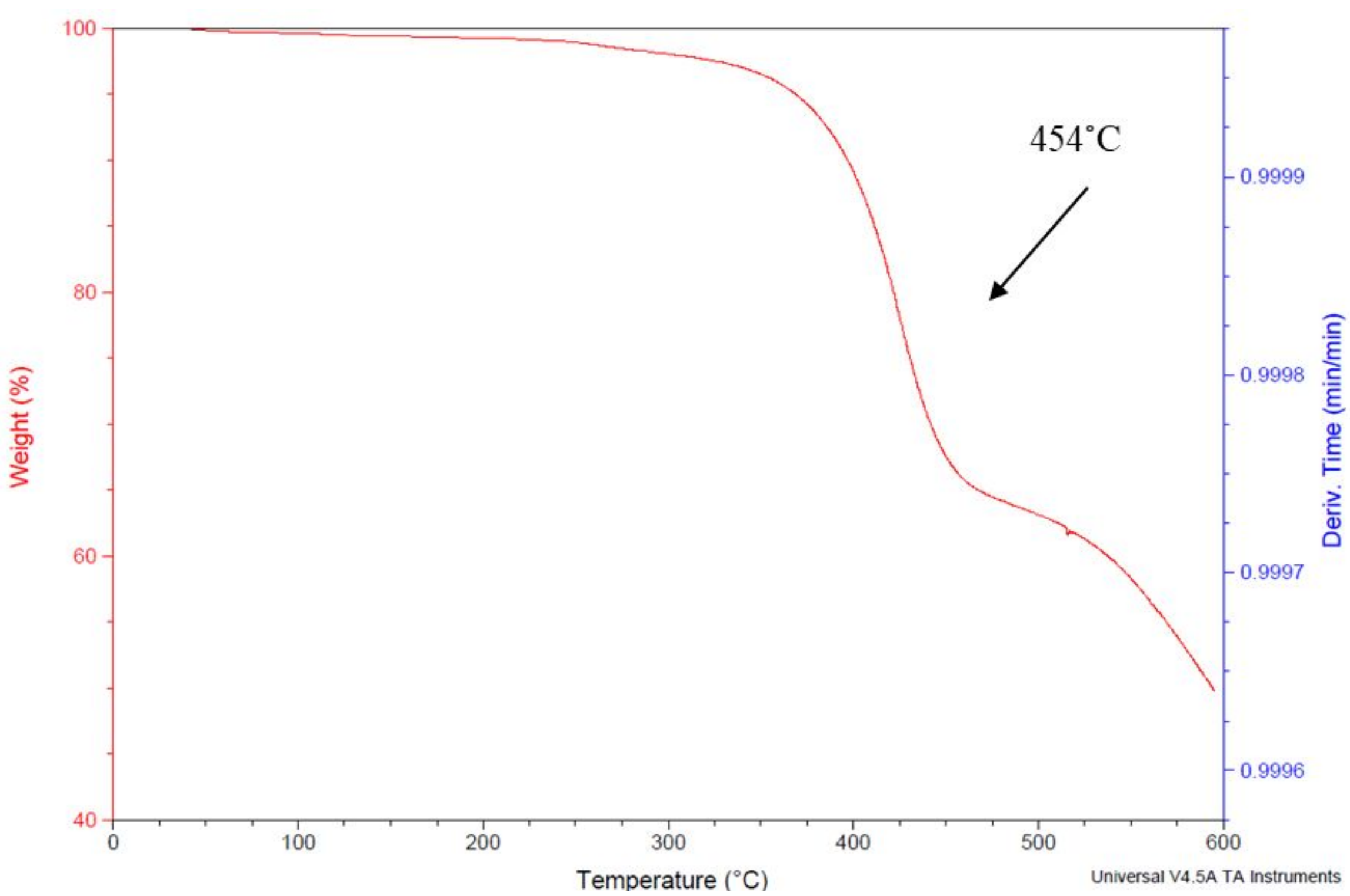

Figure S19. TGA spectrum of FlAnS-$COOCH_3$

S5. DSC

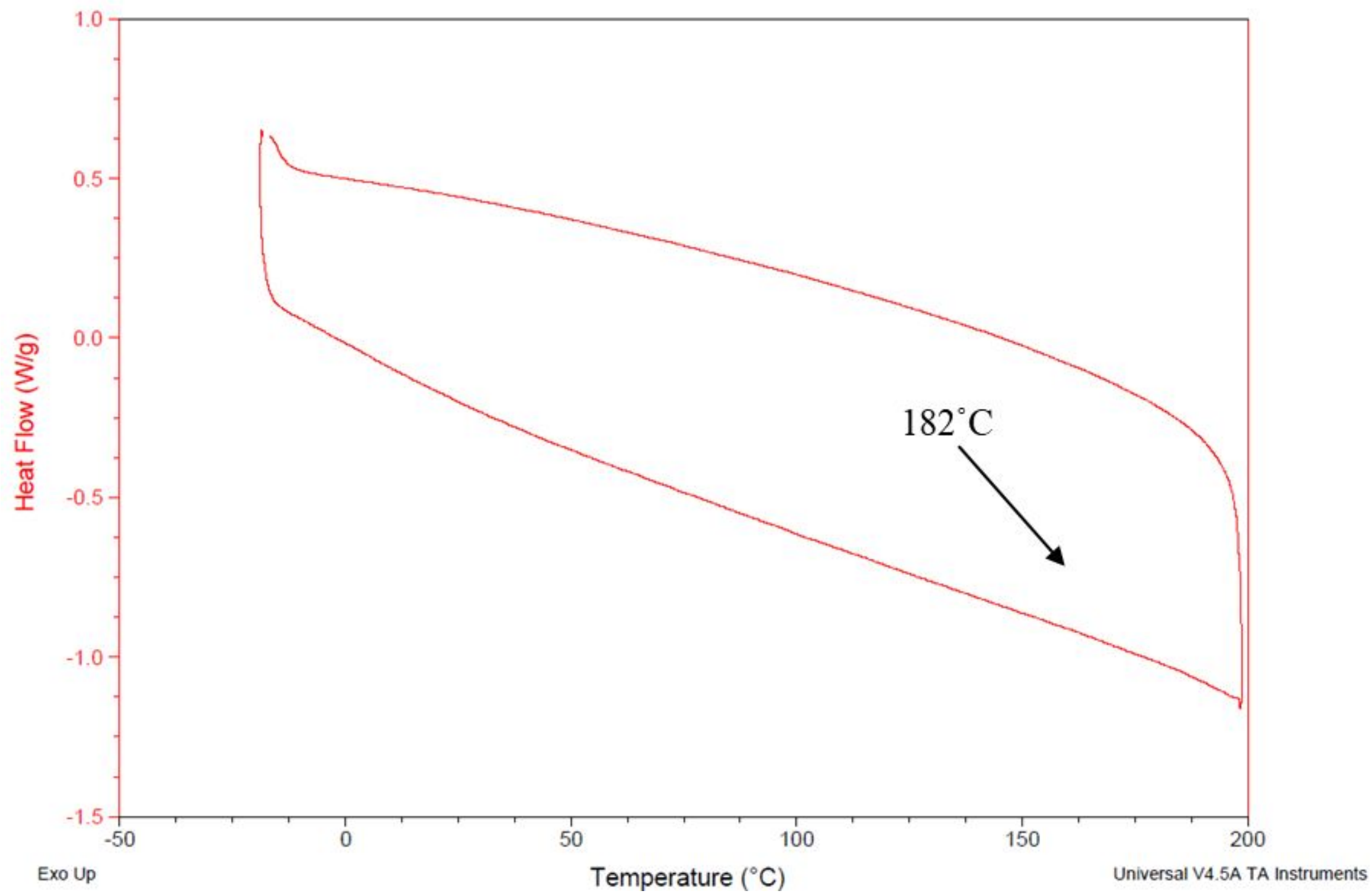

Figure S20. DSC spectrum of CzFl-N

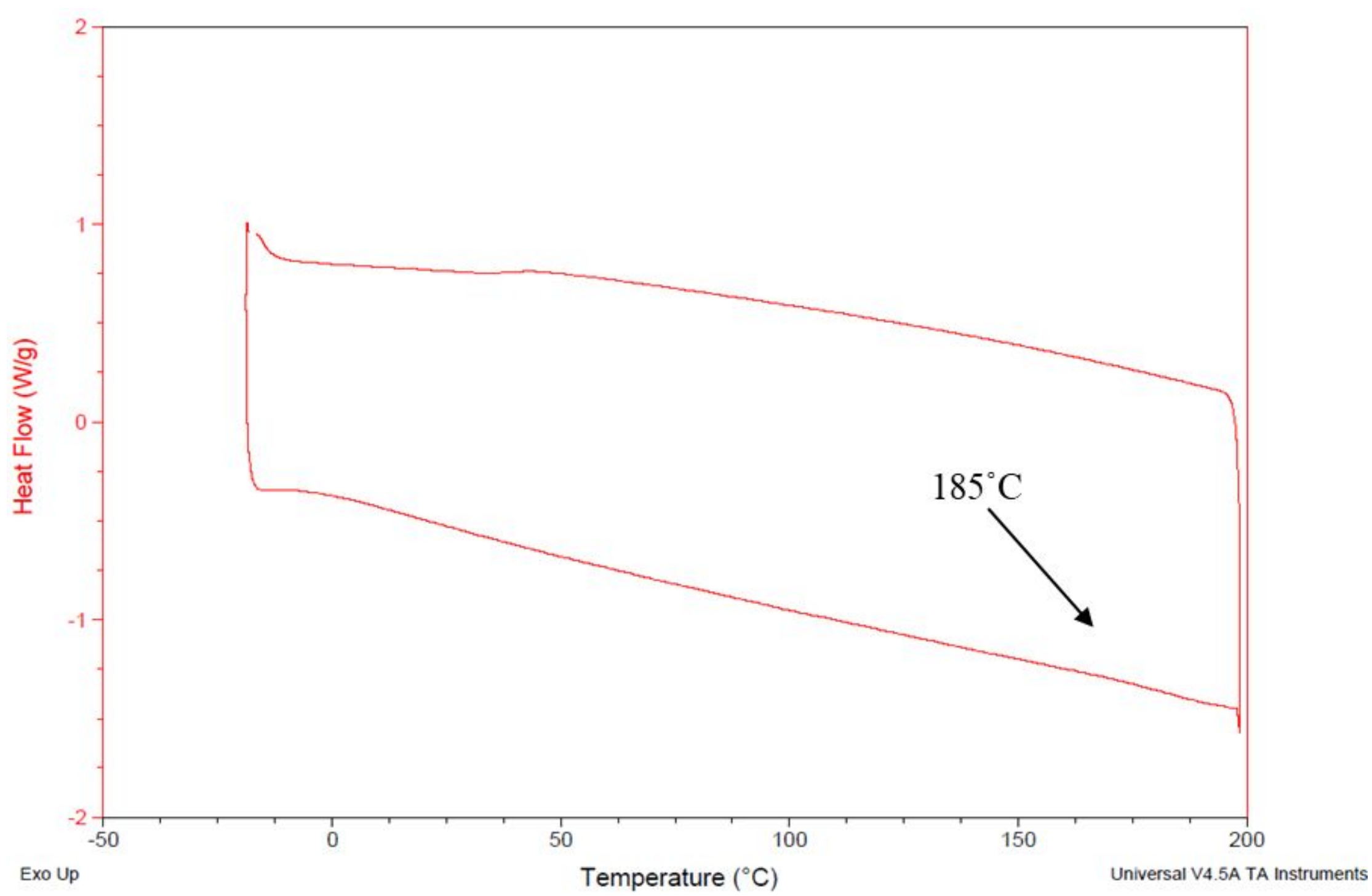

Figure S21. DSC spectrum of CzFl-$COOCH_3$

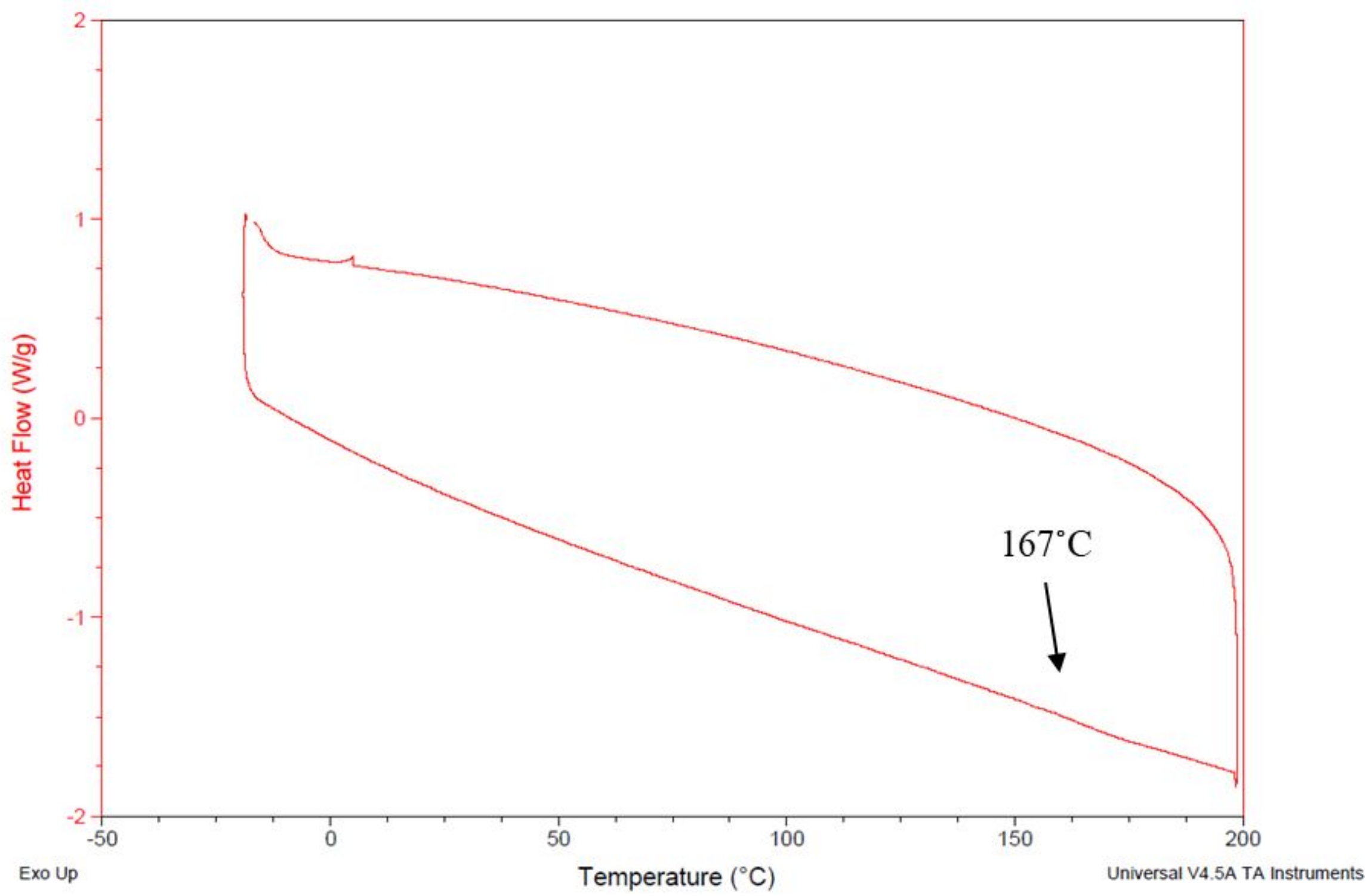

Figure S22. DSC spectrum of FlCz-N

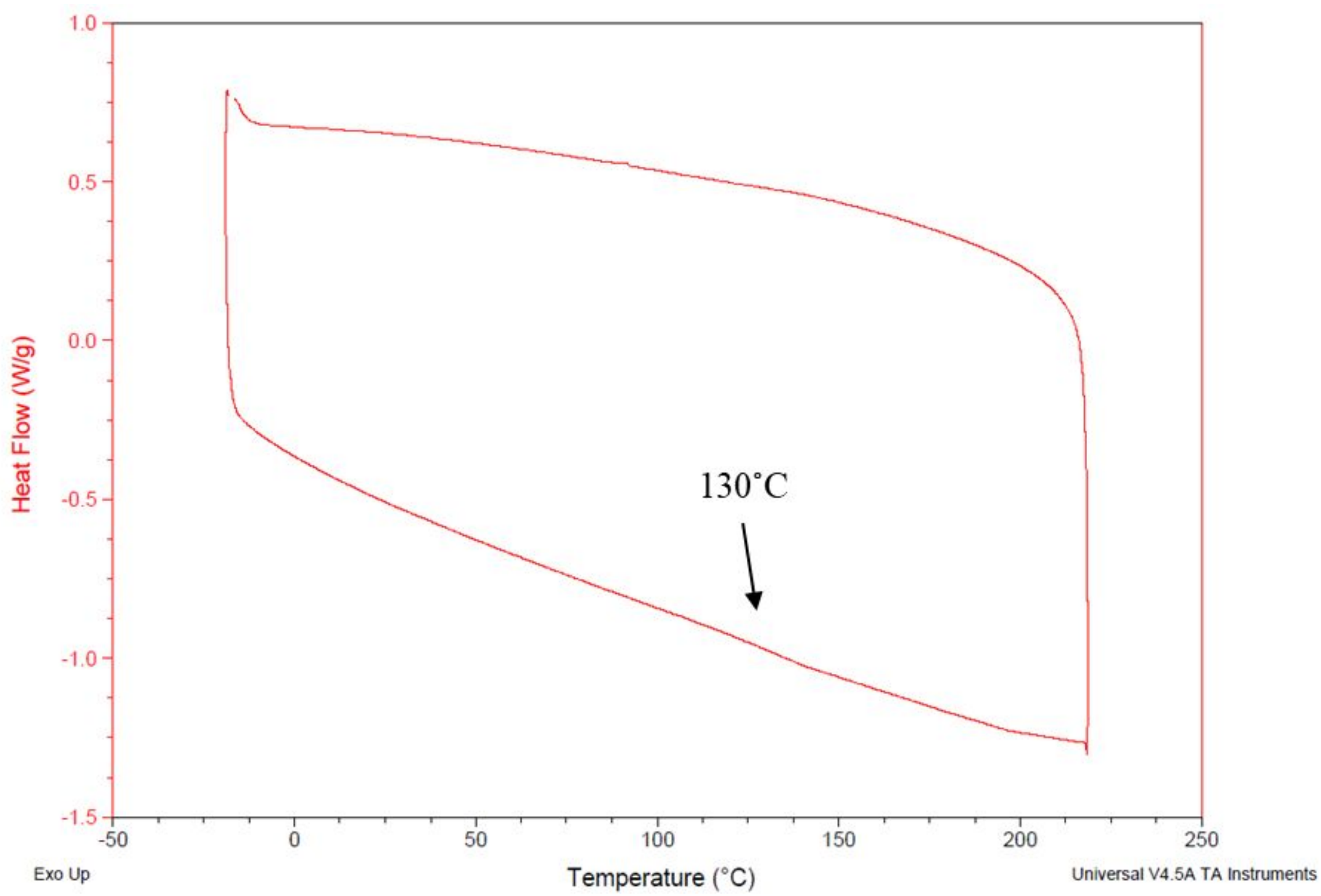

Figure S23. DSC spectrum of FlCz-$COOCH_3$

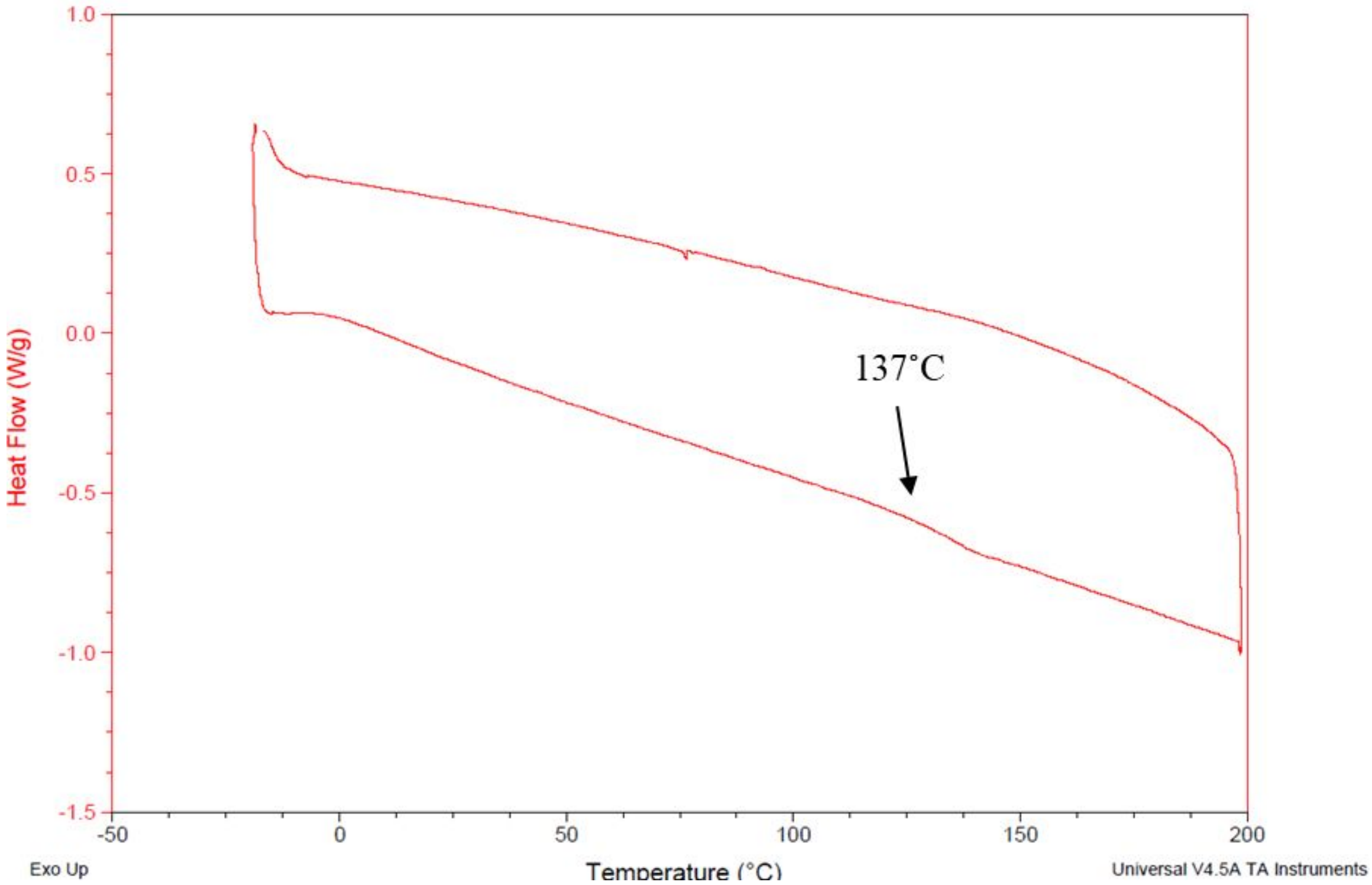

Figure S24. DSC spectrum of CzAn-N

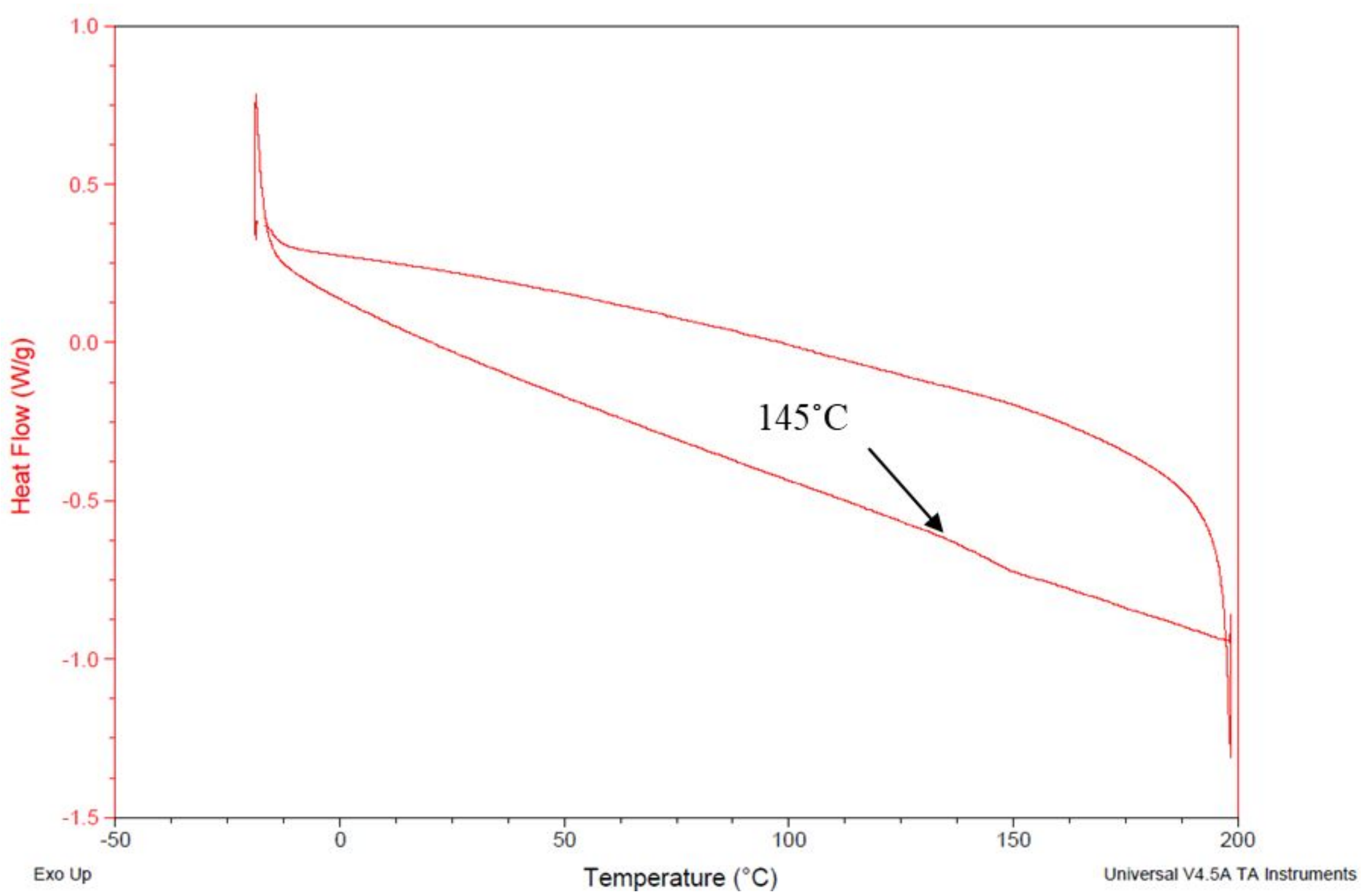

Figure S25. DSC spectrum of CzAn-$COOCH_3$

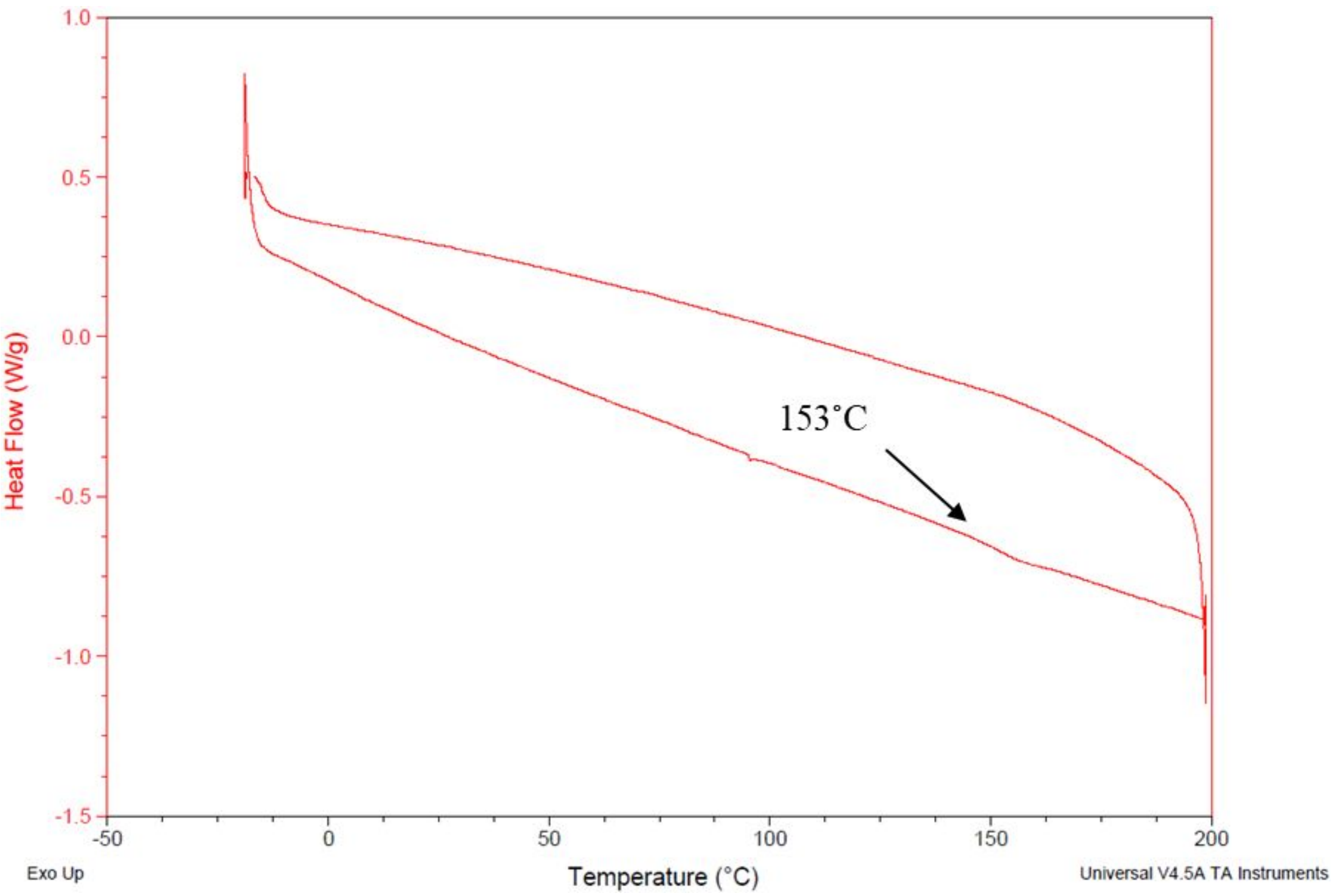

Figure S26. DSC spectrum of FlAnS-N

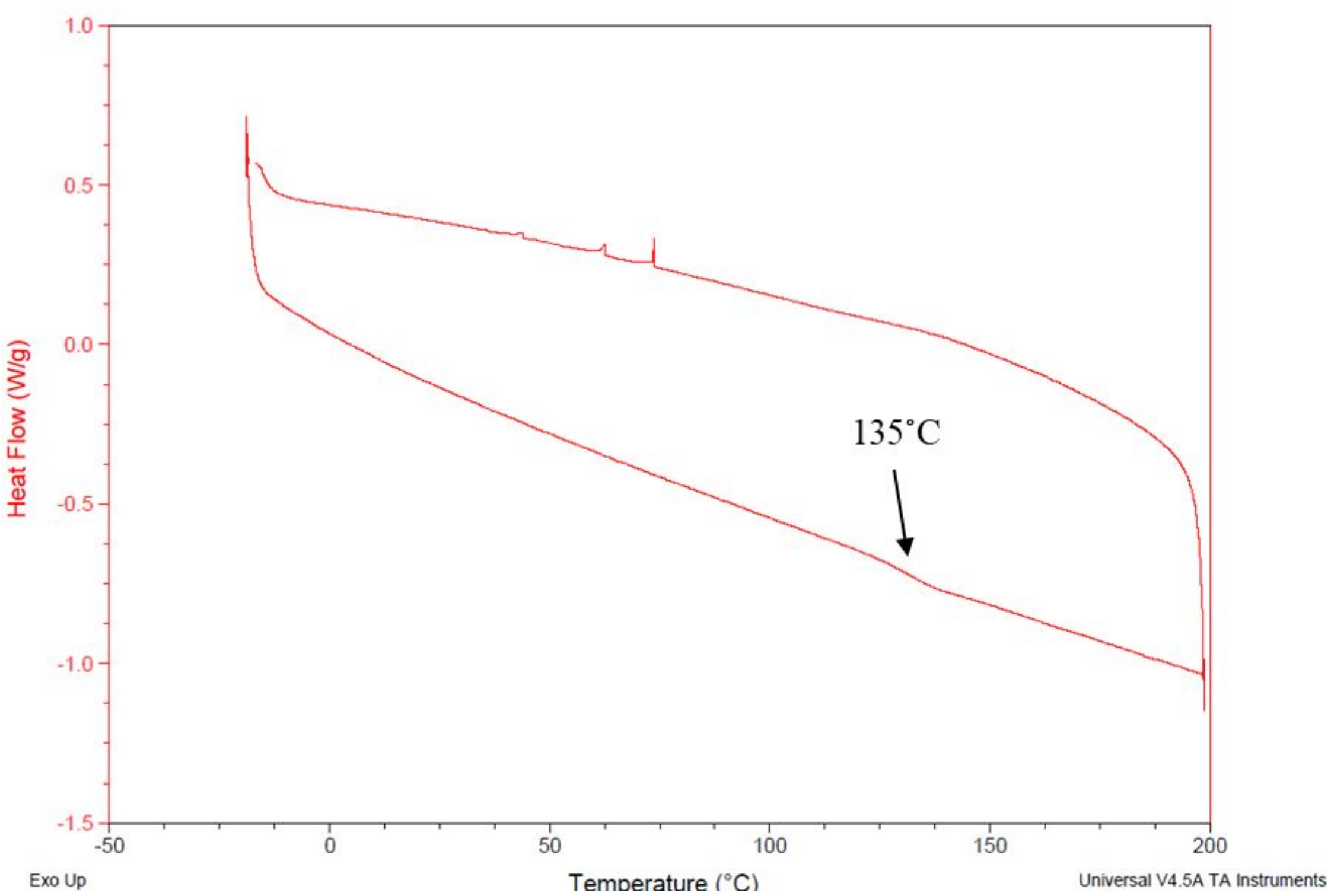

Figure S27. DSC spectrum of FlAnS-$COOCH_3$

S6. UV-Vis and Work function Measurements

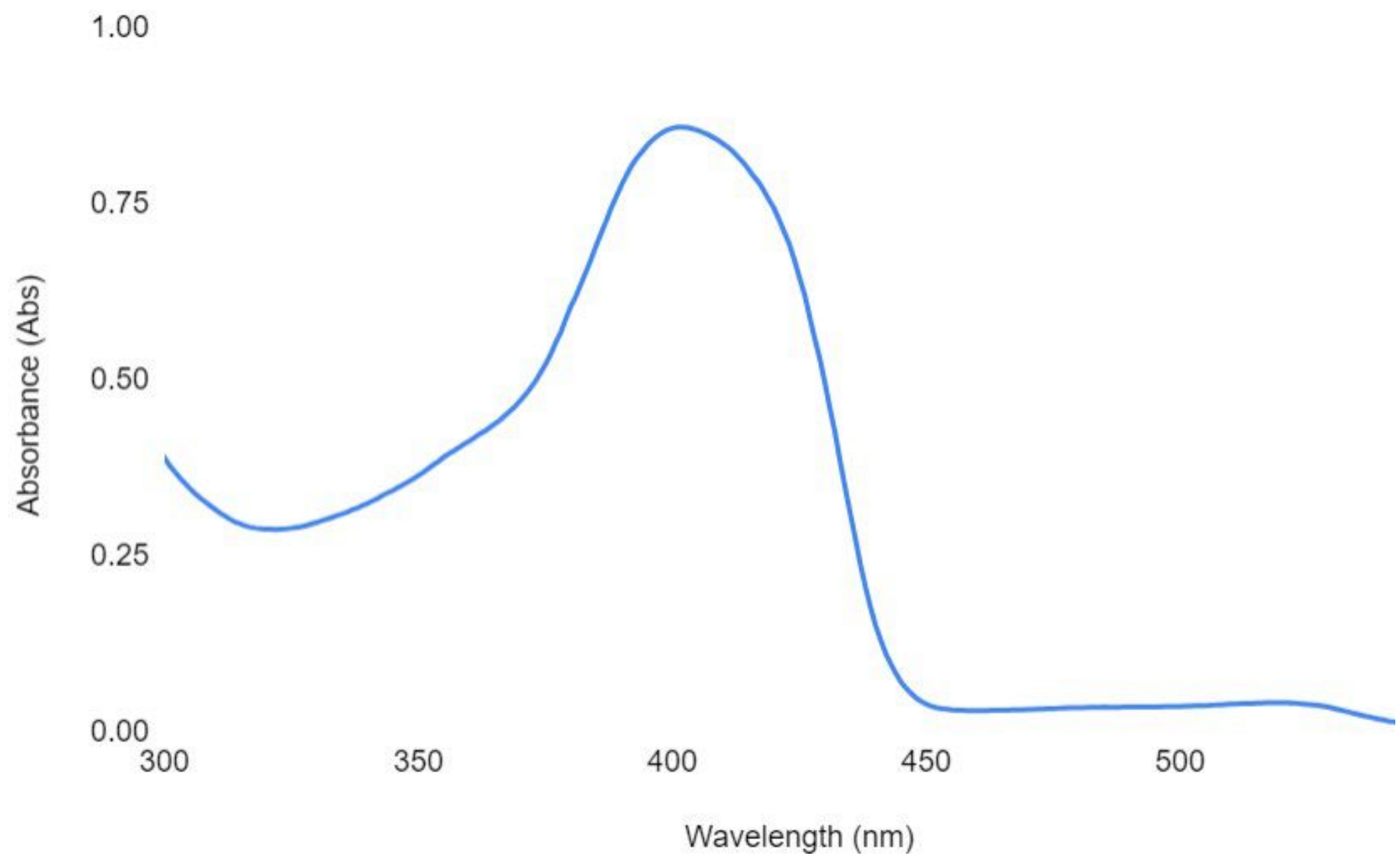

Figure S28. UV-Vis spectrum of CzFl-N

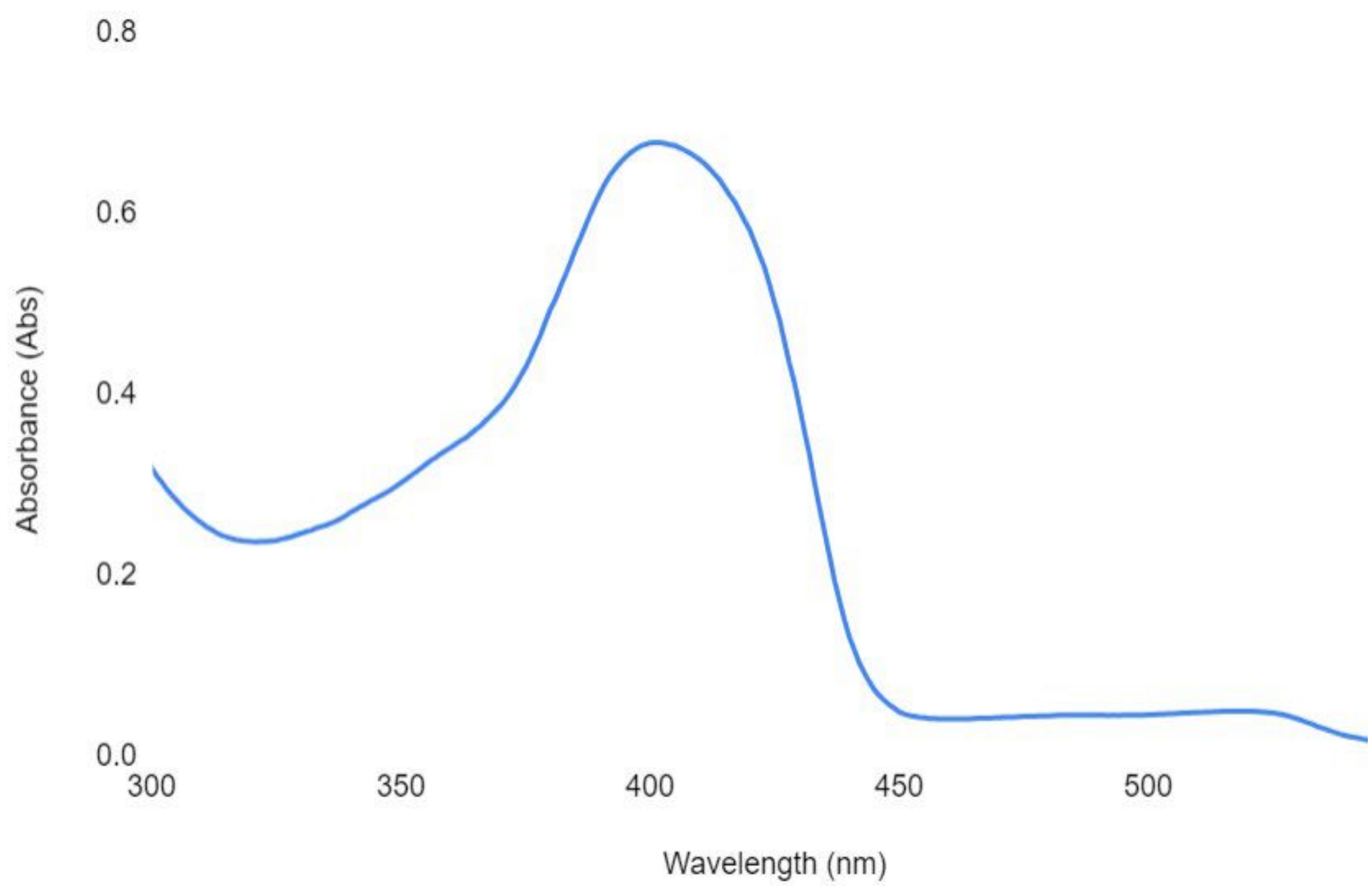

Figure S29. UV-Vis spectrum of CzFl-$COOCH_3$

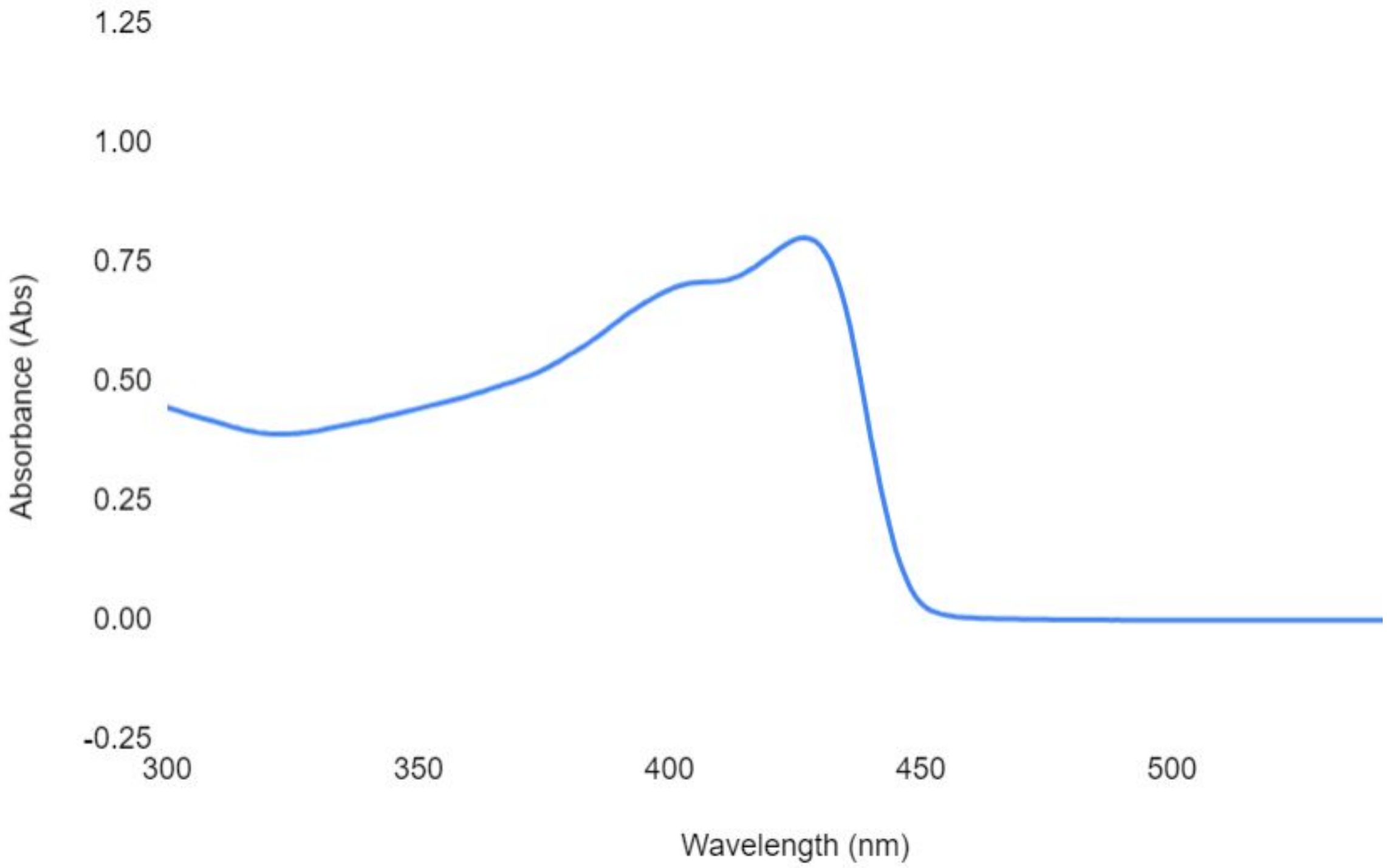

Figure S30. UV-Vis spectrum of FlCz-N

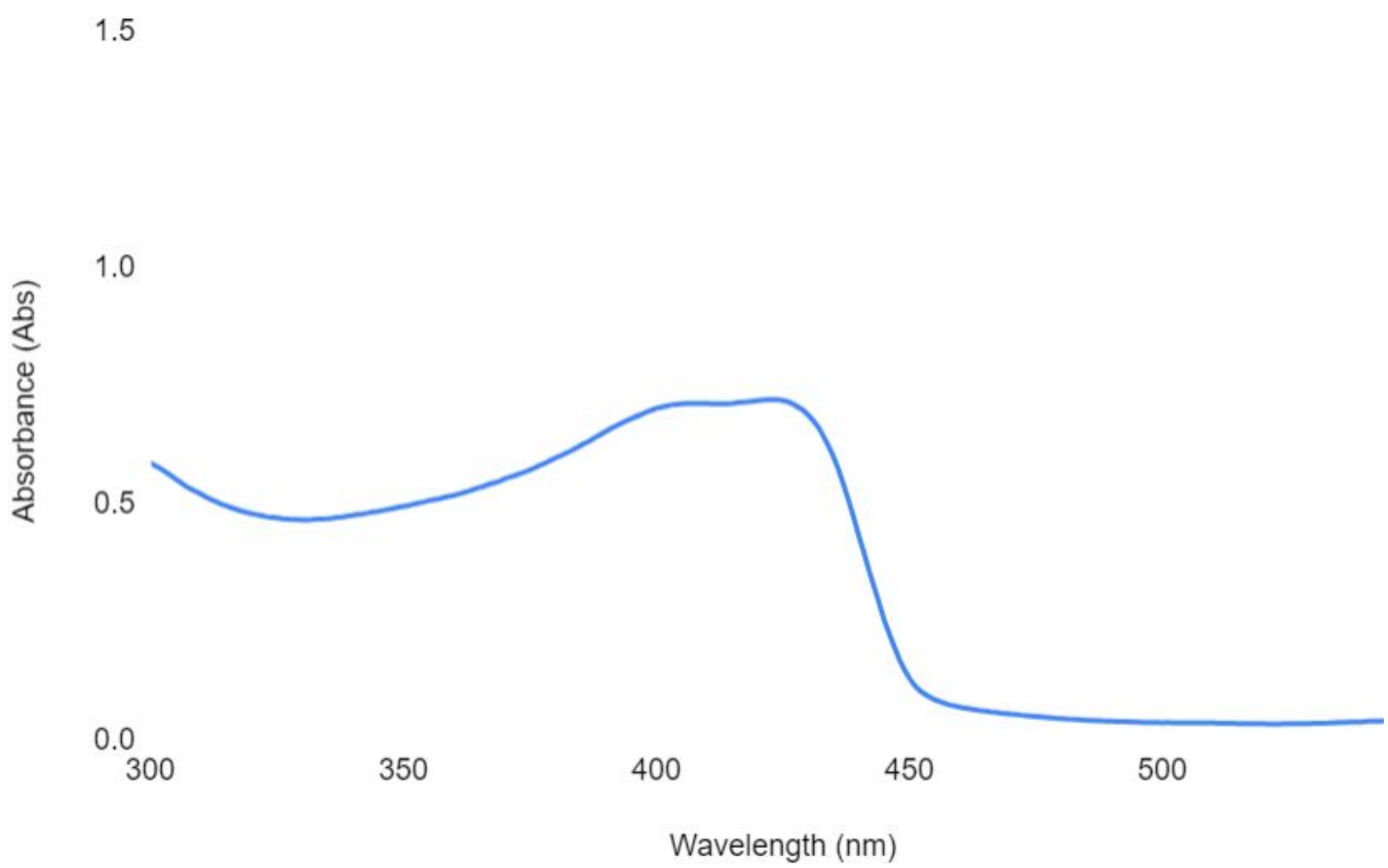

Figure S31. UV-Vis spectrum of FlCz-$COOCH_3$

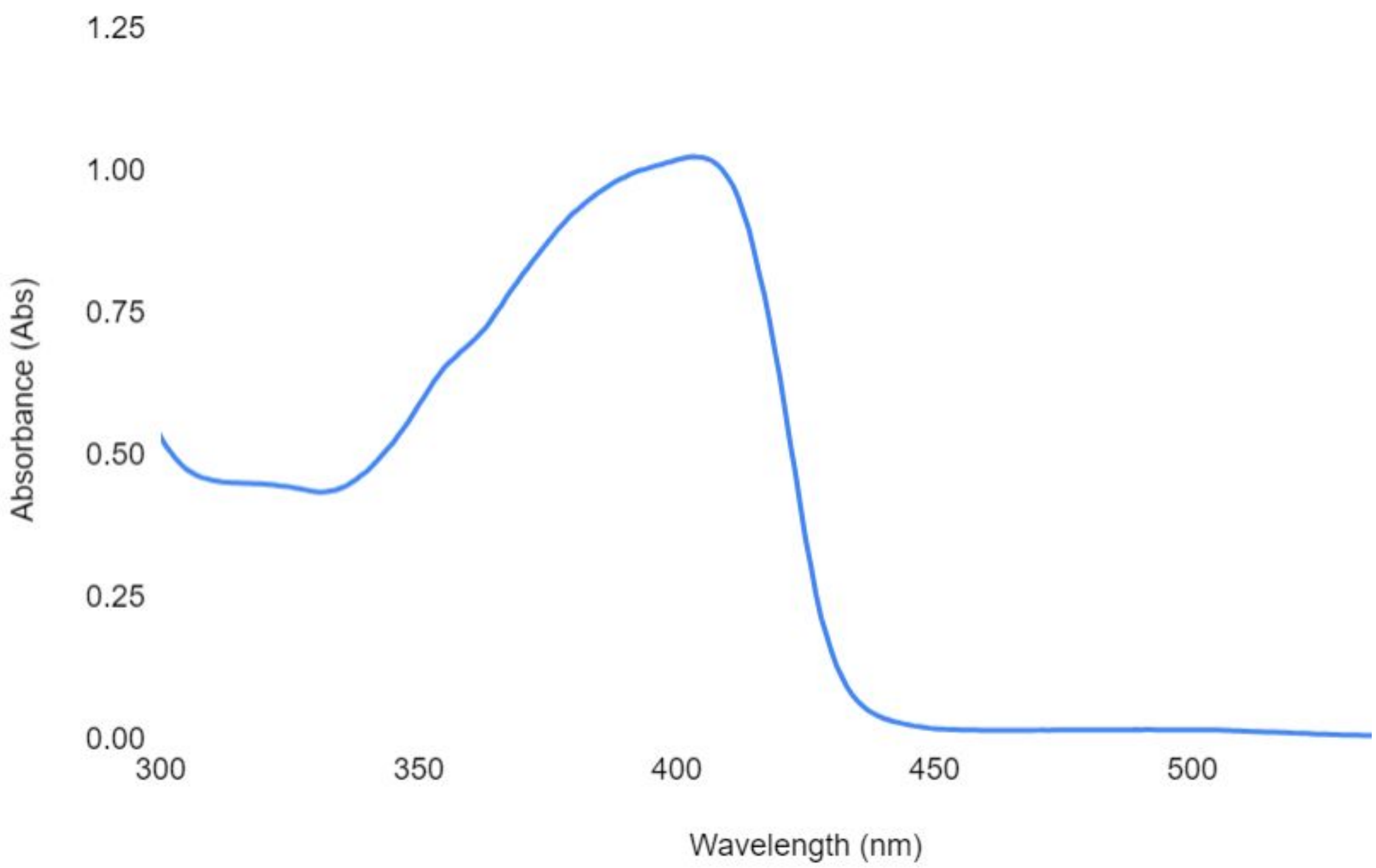

Figure S32. Uv-Vis spectrum of CzAn-N

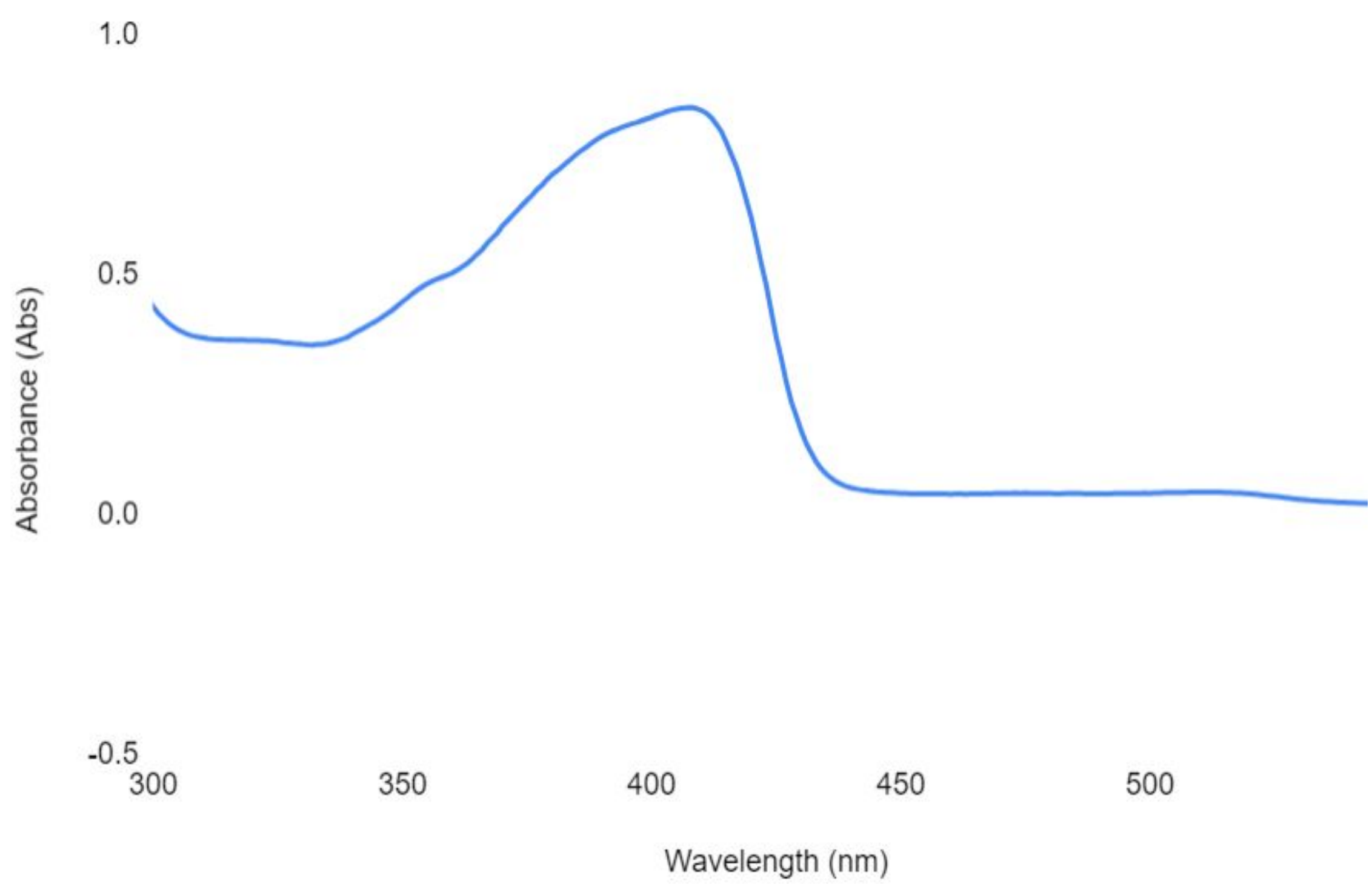

Figure S33. UV-Vis spectrum of CzAn-$COOCH_3$

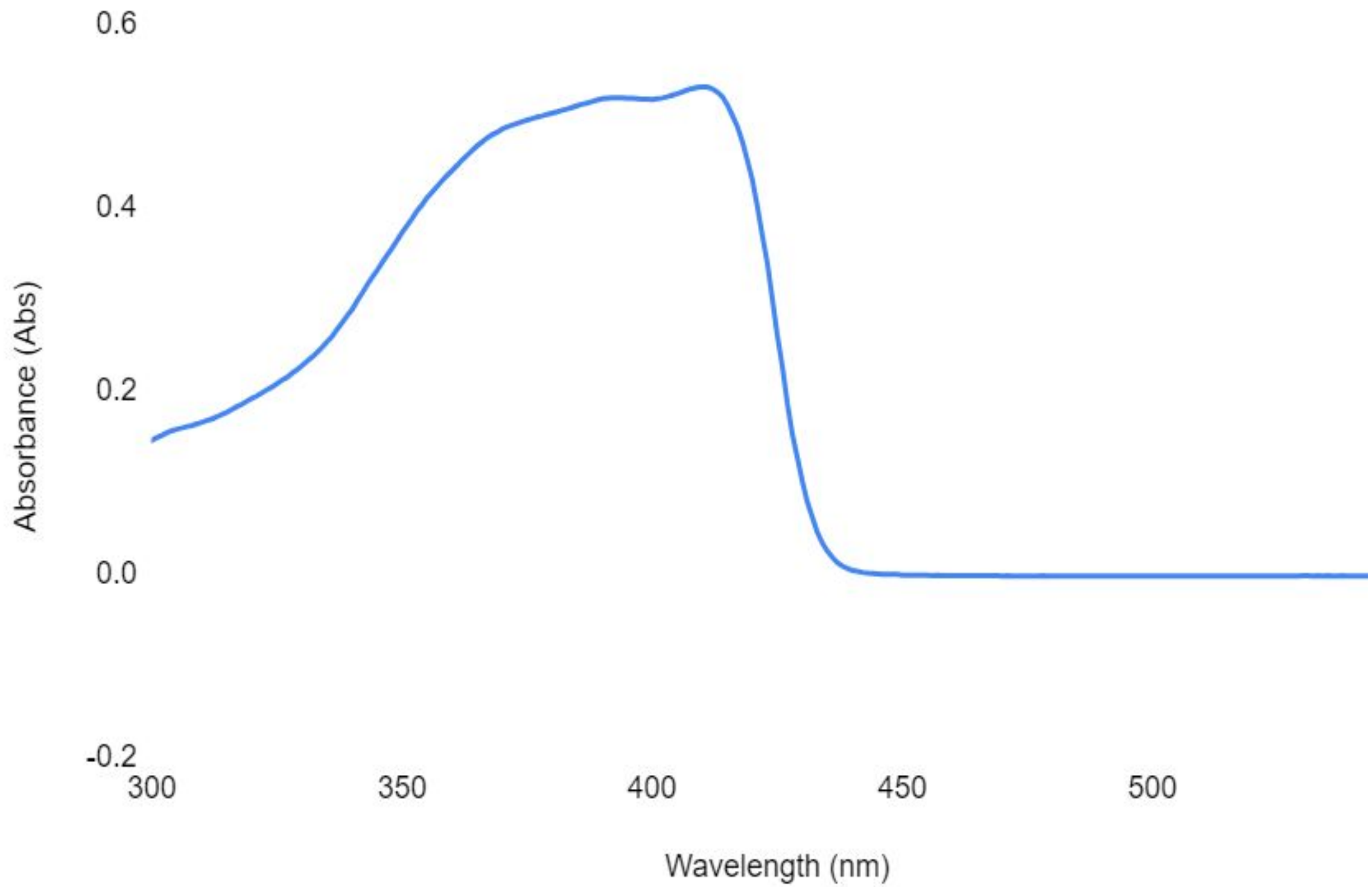

Figure S34. UV-Vis spectrum of FlAnS-N

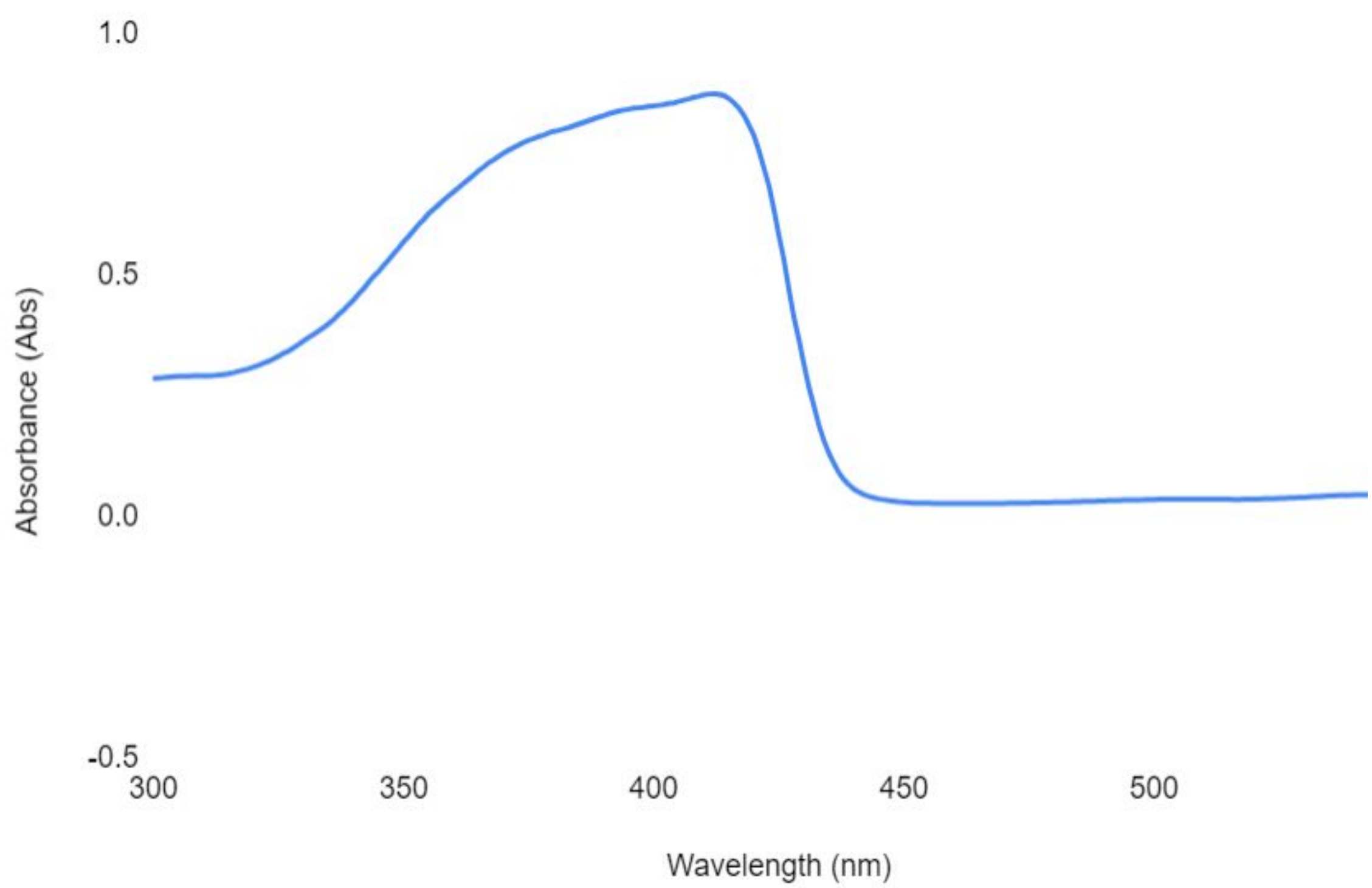

Figure S35. UV-Vis spectrum of FlAnS-$COOCH_3$

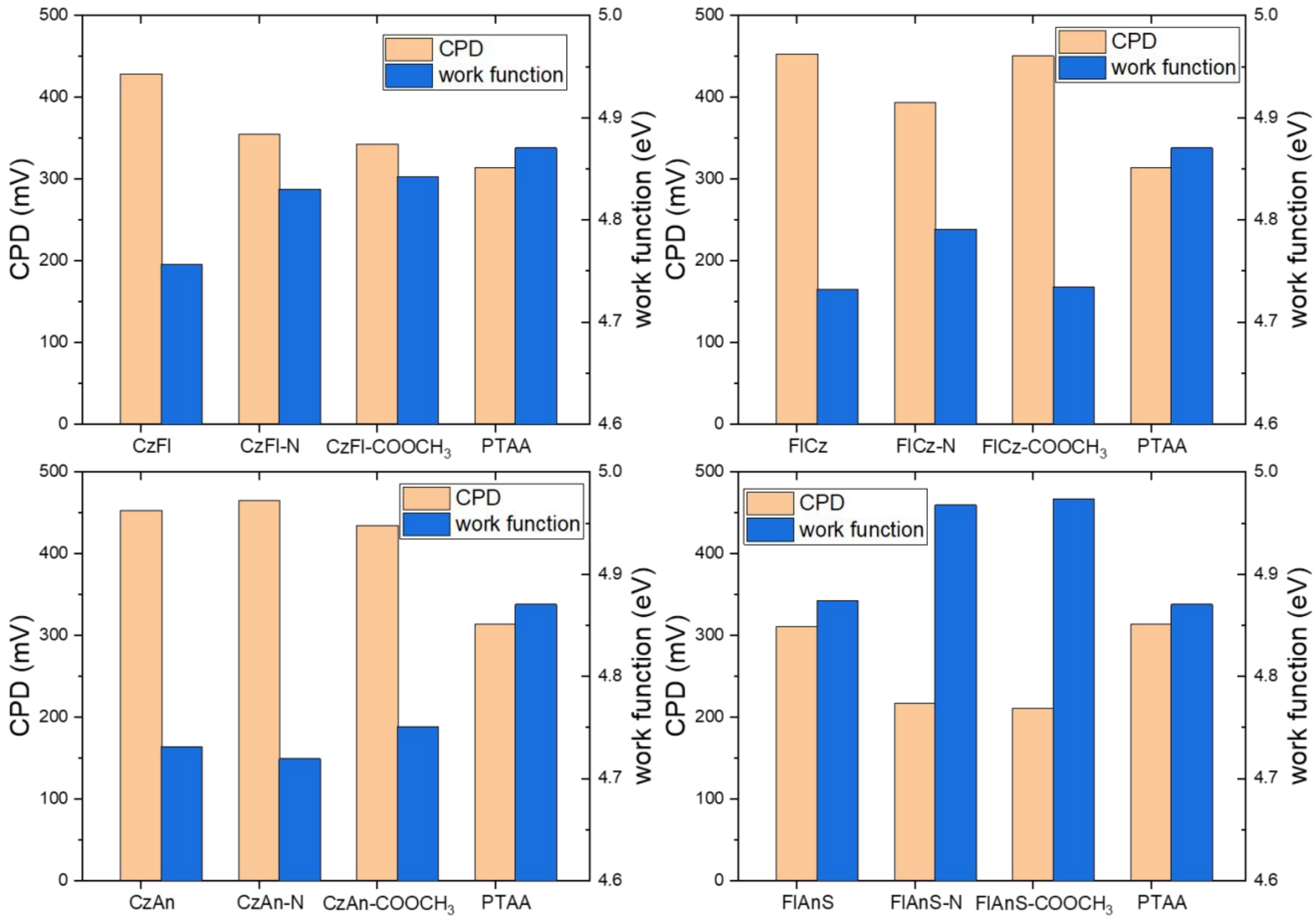

Figure S36. KPFM data showing CPD and work function for different polymer HTMs.

S7. Comparison of PHTM M_n

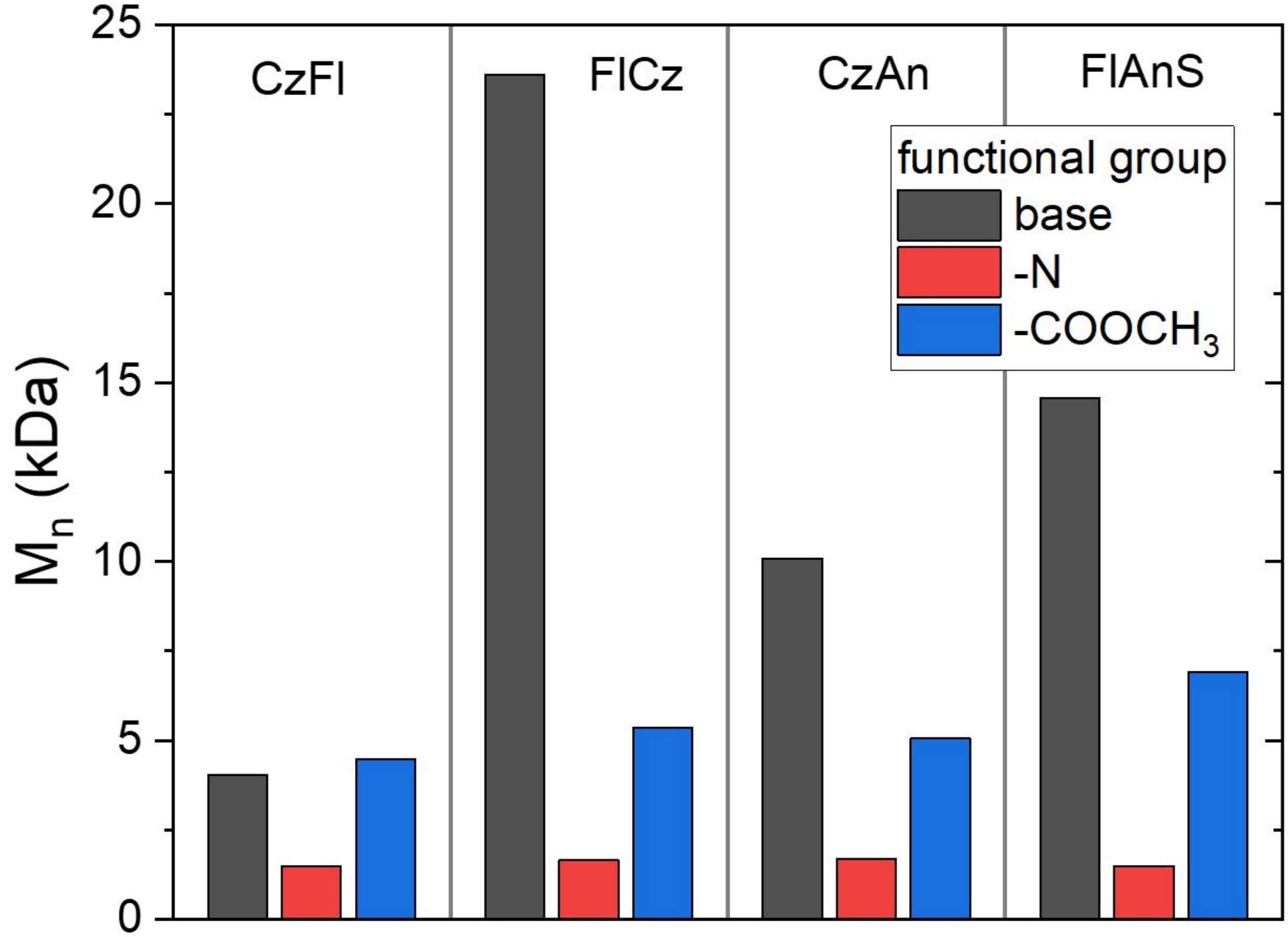

Figure S37. Comparison of M_n of wettable PHTM to parent polymers determined against polystyrene standards.

S8. Water Contact Angle

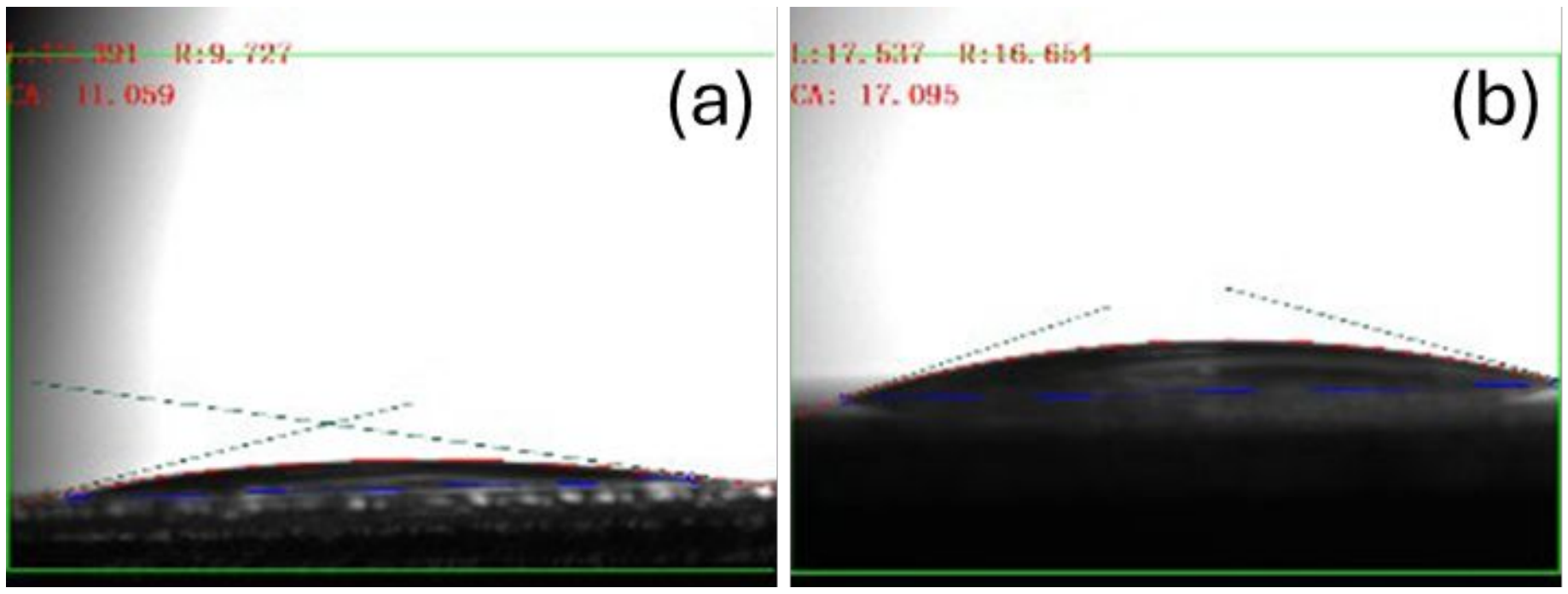

Figure S38. Contact angle measurement of (a) CzFl-N (b) CzFl-$COOCH_3$

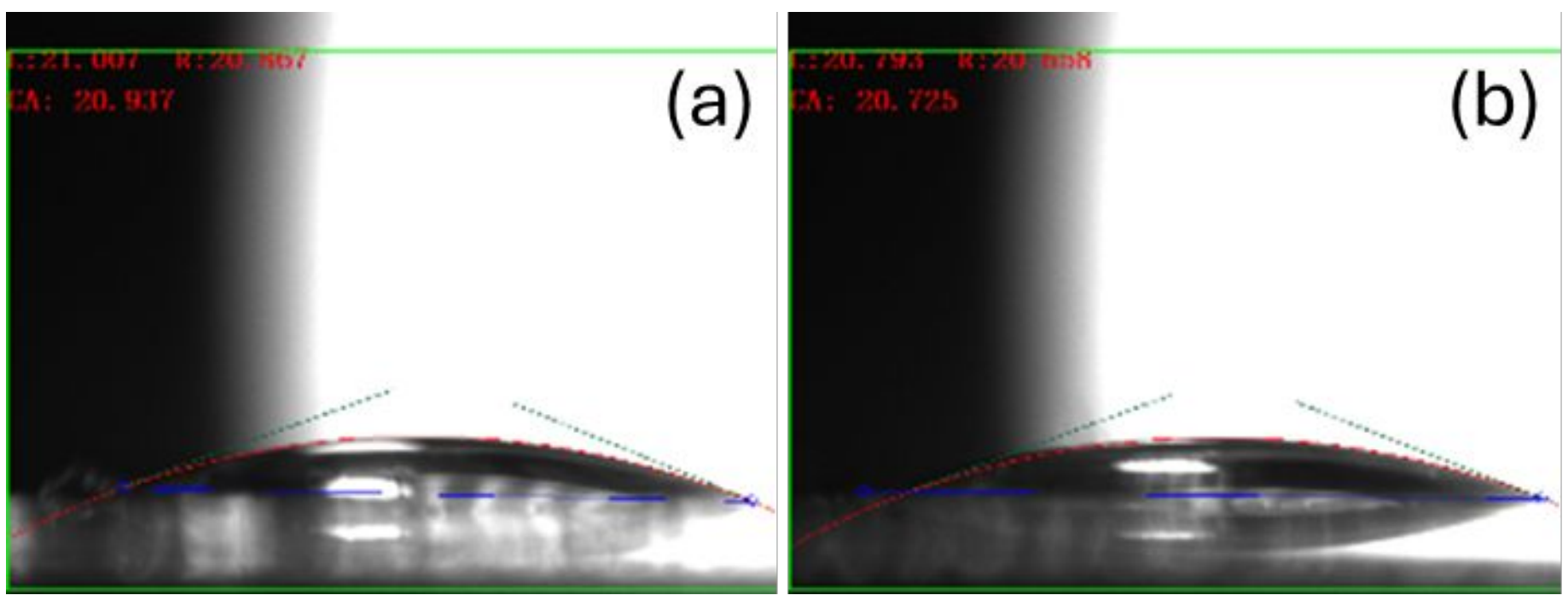

Figure S39. Contact angle measurement of (a)FlCz-N (b) FlCz-$COOCH_3$

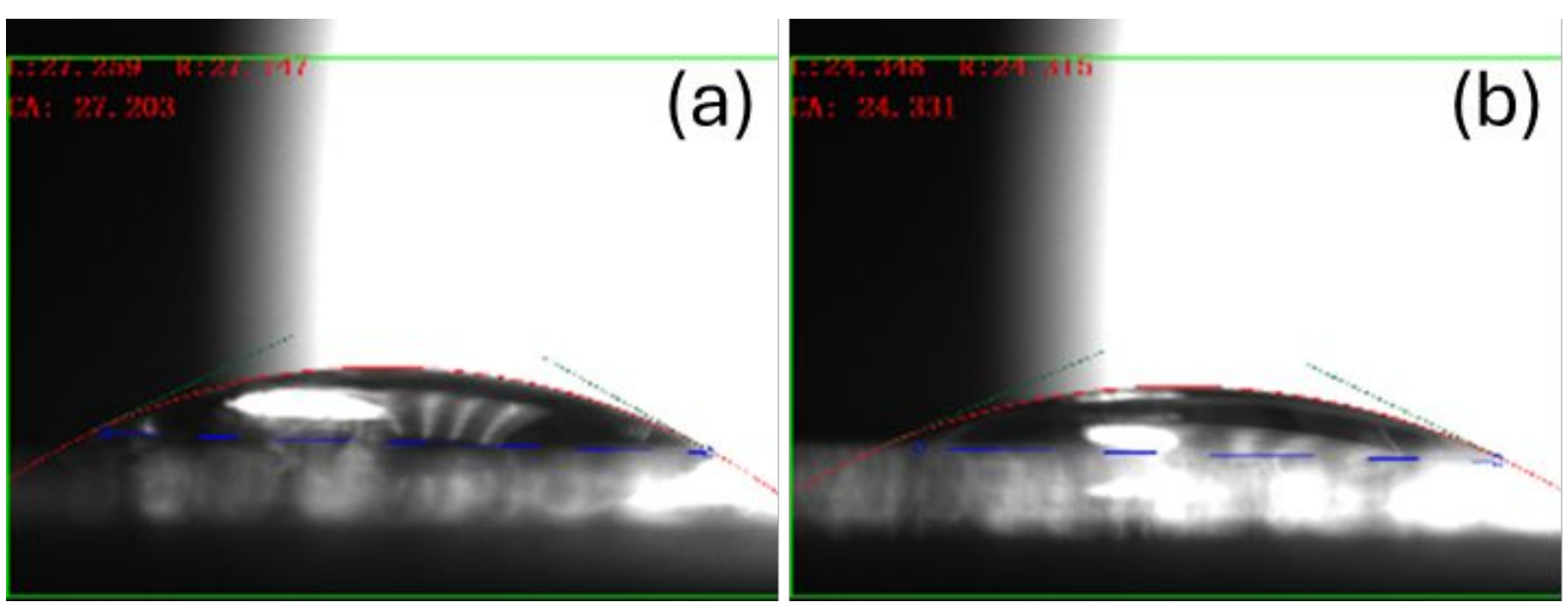

Figure S40. Contact angle measurement of (a) CzAn-N (b) CzAn-$COOCH_3$

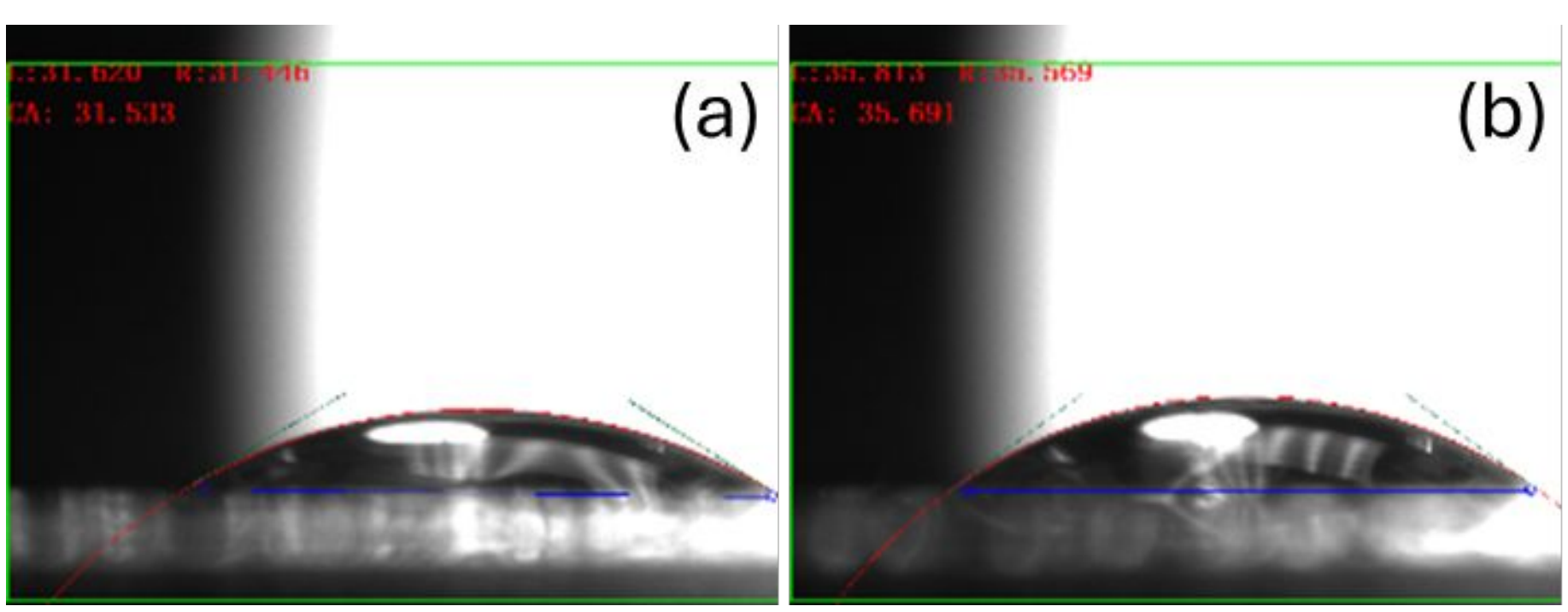

Figure S41. Contact angle measurement of (a) FlAnS-N (b) FlAnS-$COOCH_3$

S9. AFM

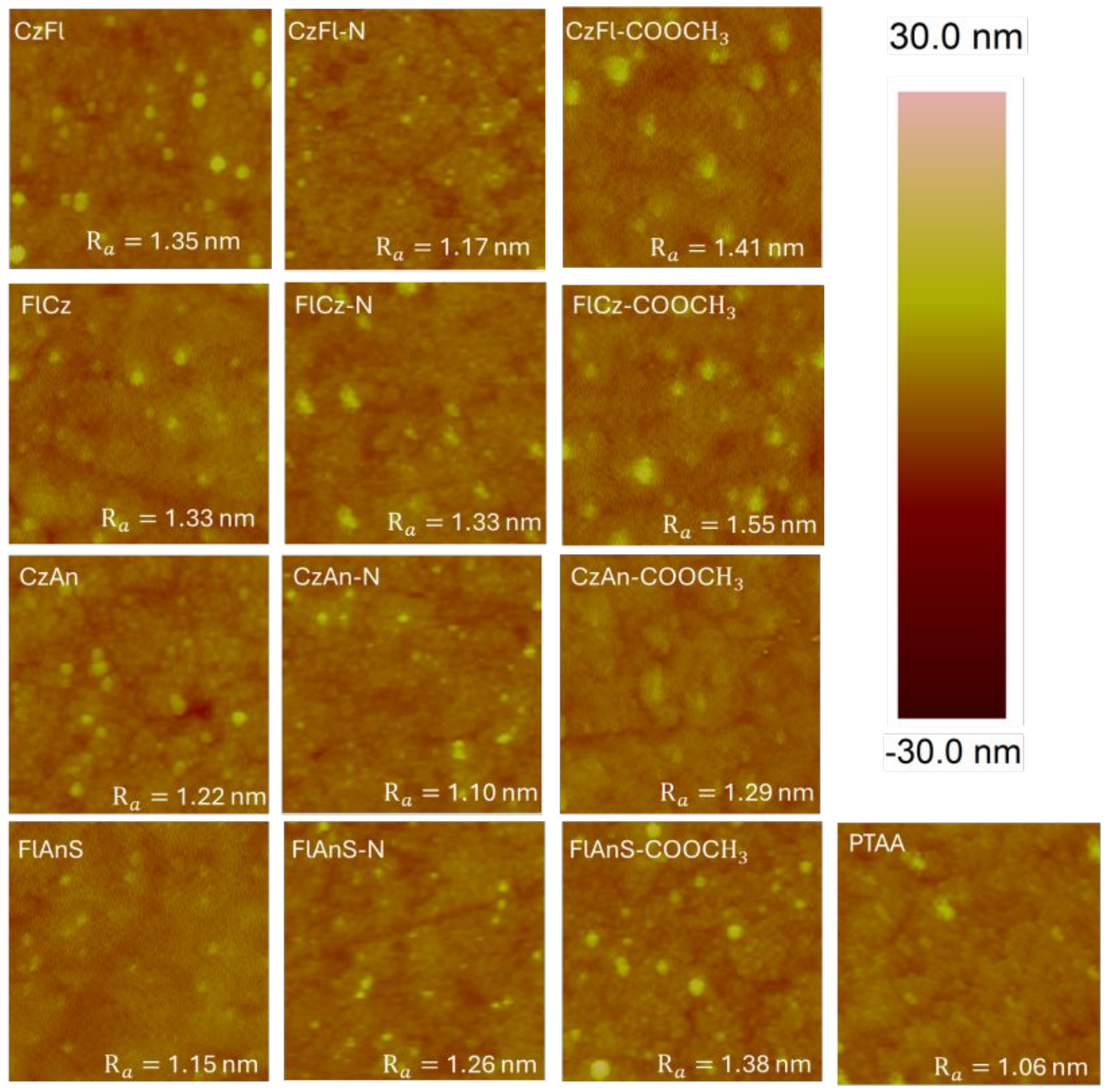

Figure S42. AFM images for different polymer HTMs coated on ITO substrates.

S10. Precursor Contact Angle

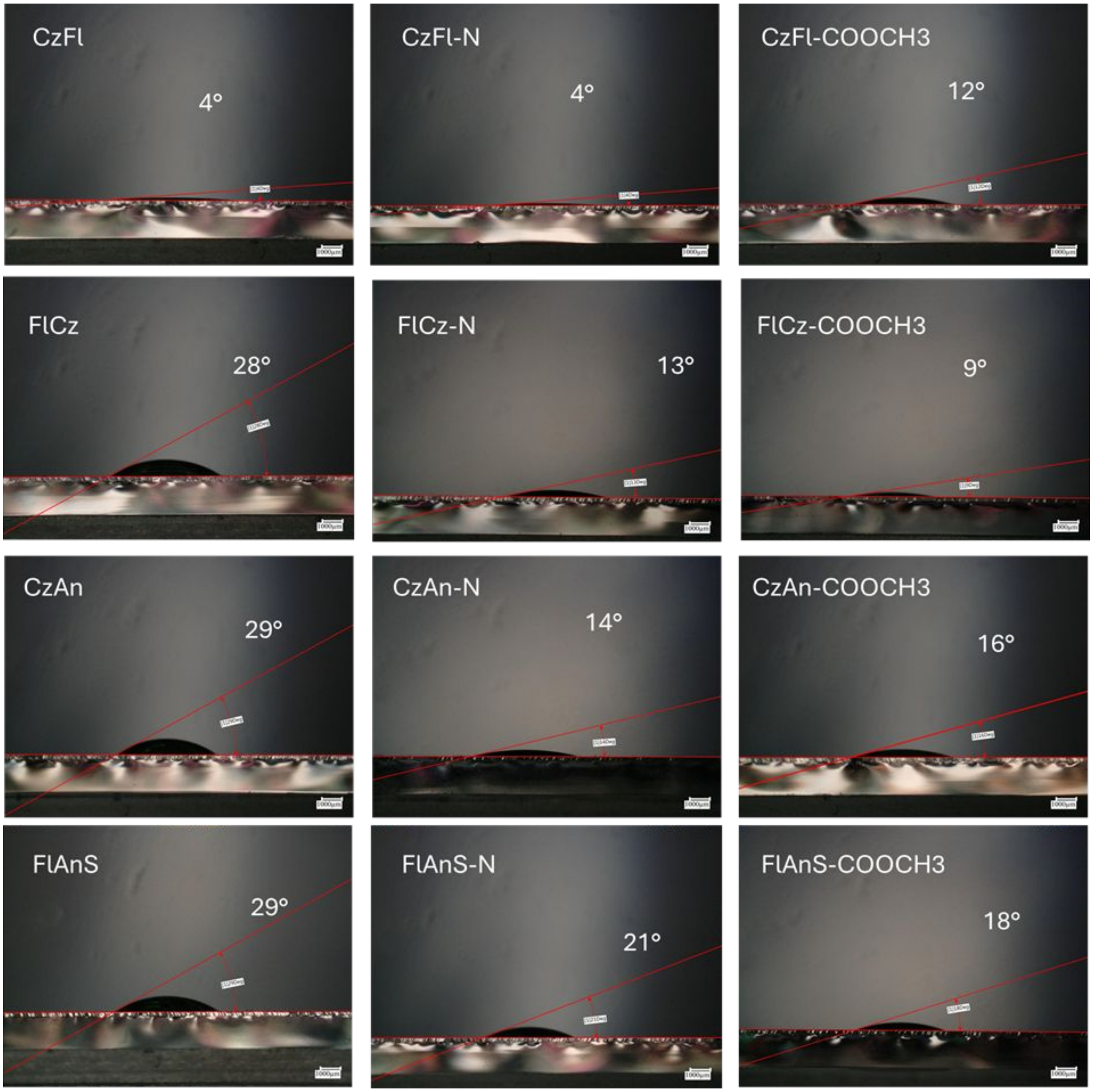

Figure S43. Images of contact angle measurement with perovskite solution on different polymer HTMs.

S11. Precursor Film Coverage

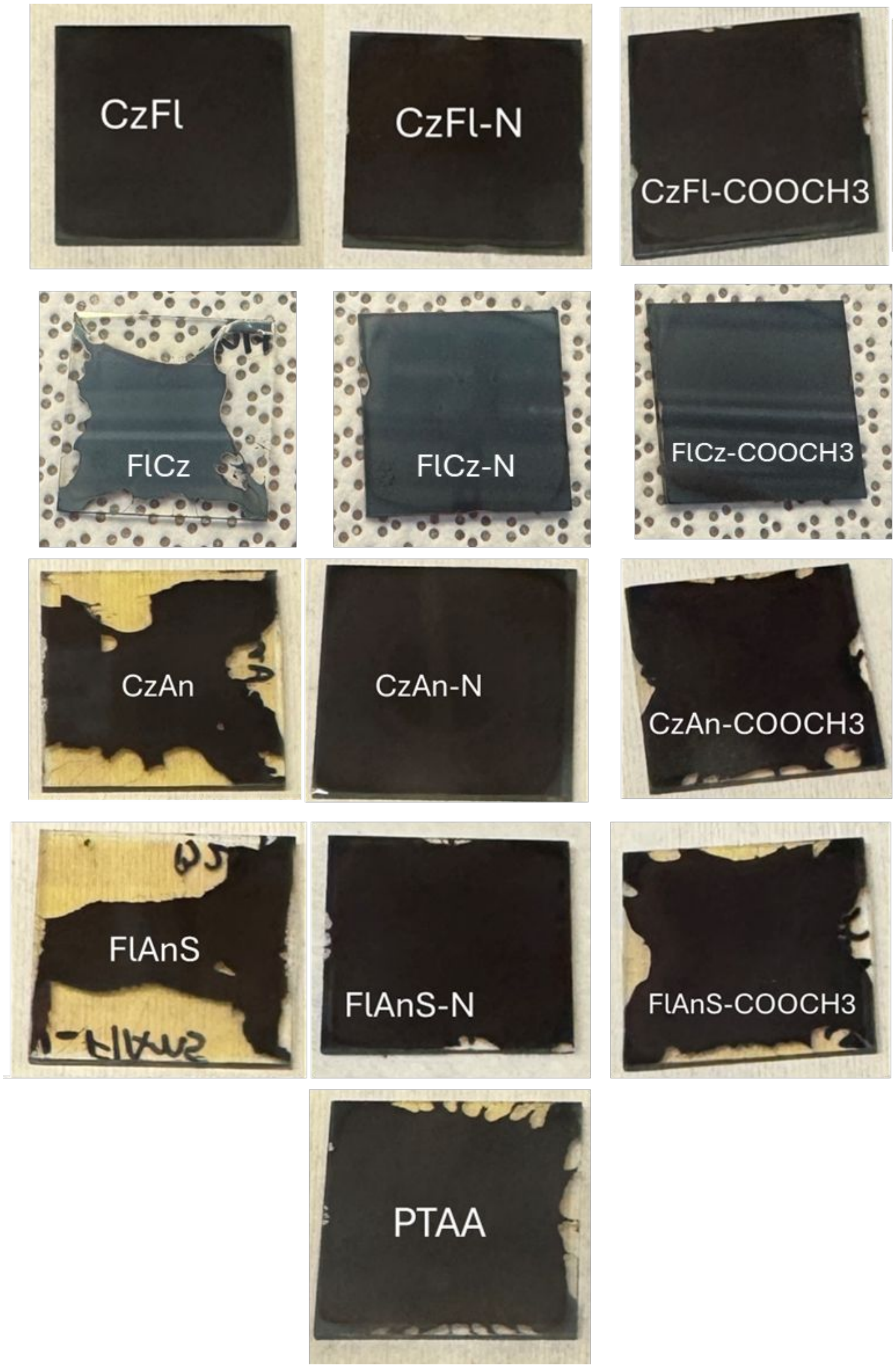

Figure S44. Photos of perovskite films deposited on different polymer HTMs.

S12. Perovskite film and Device Characterization

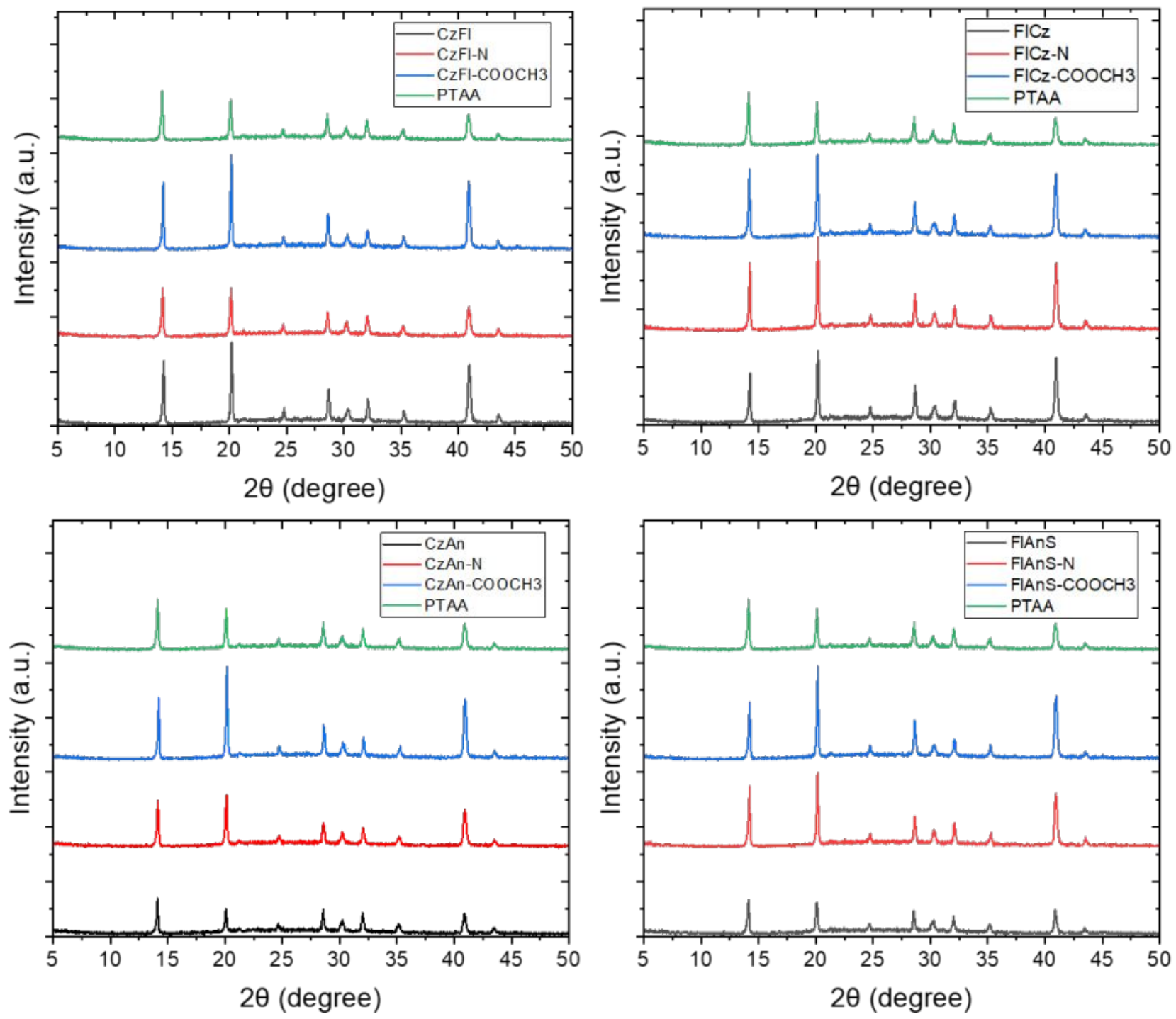

Figure S45. XRD pattern for perovskite film deposited on different polymer HTMs.

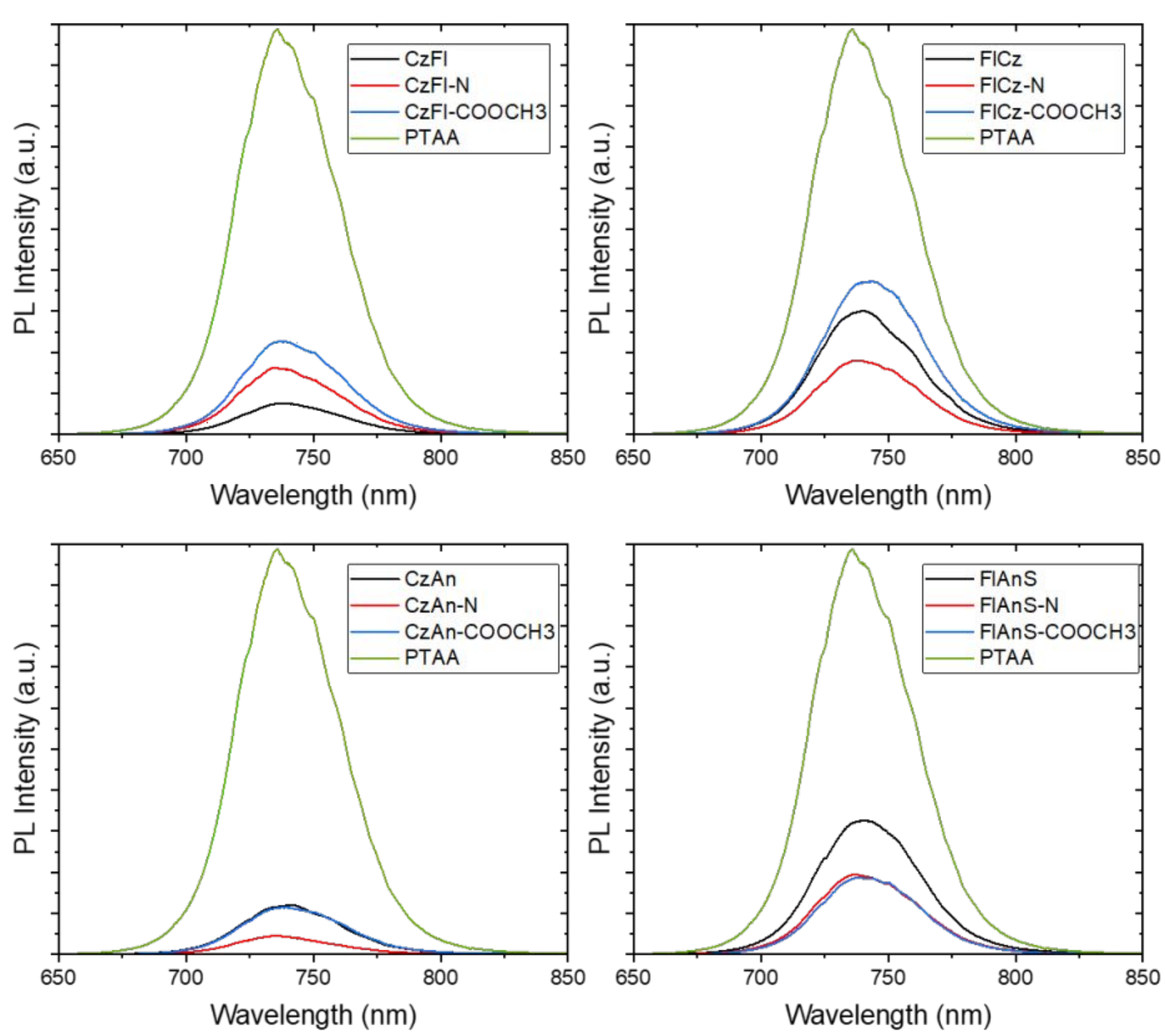

Figure S46. Steady-state PL spectra for perovskite film deposited on different polymer HTMs.

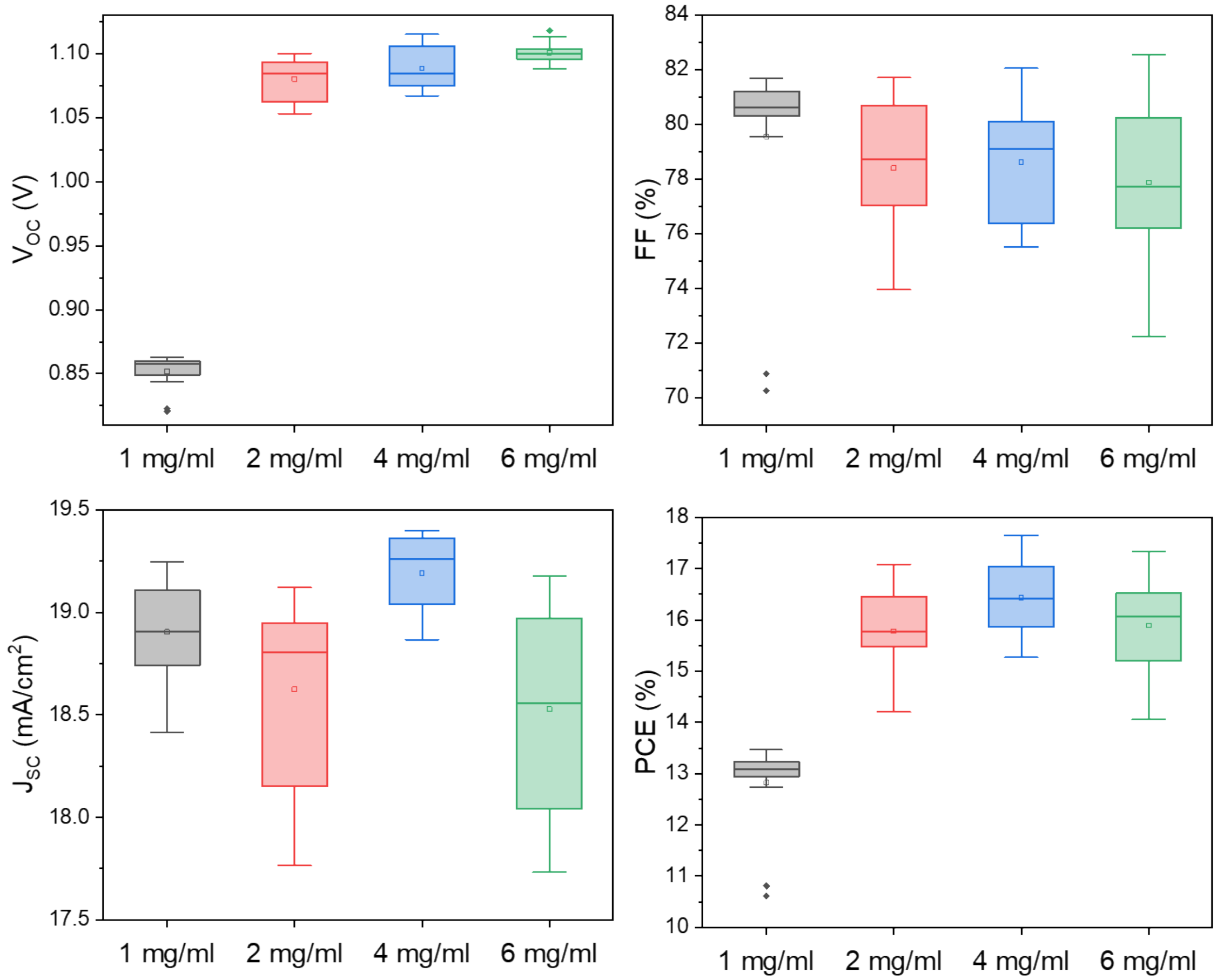

Figure S47. J-V parameter variation with different concentration of CzFl-N PHTM.

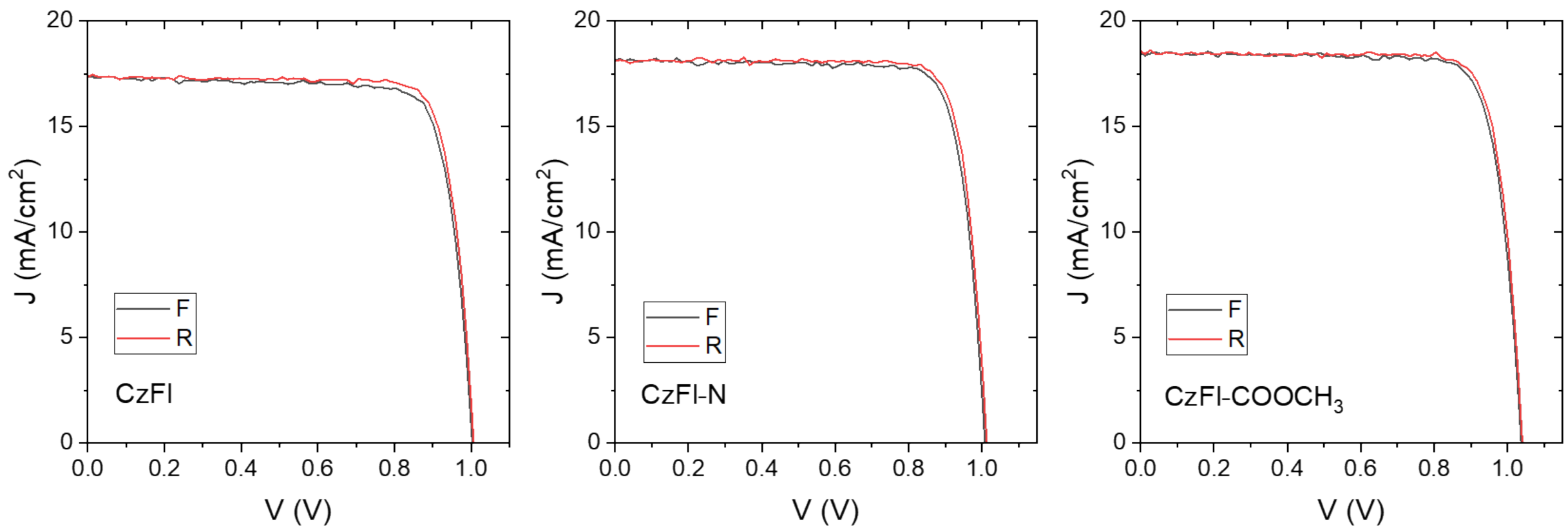

Figure S48. J-V curves of champion devices with CzFl, CzFl-N and CzFl-COOCH$_3$ PHTM.

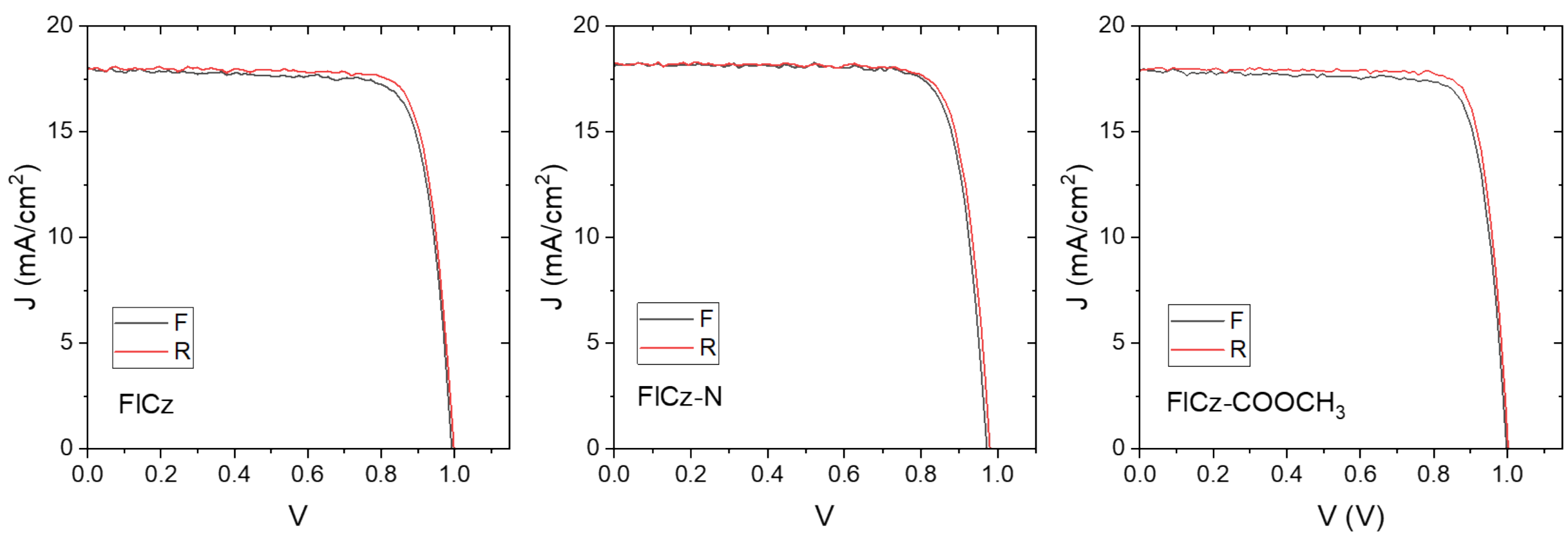

Figure S49. J-V curves of champion devices with FlCz, FlCz-N and FlCz-COOCH$_3$ PHTM.

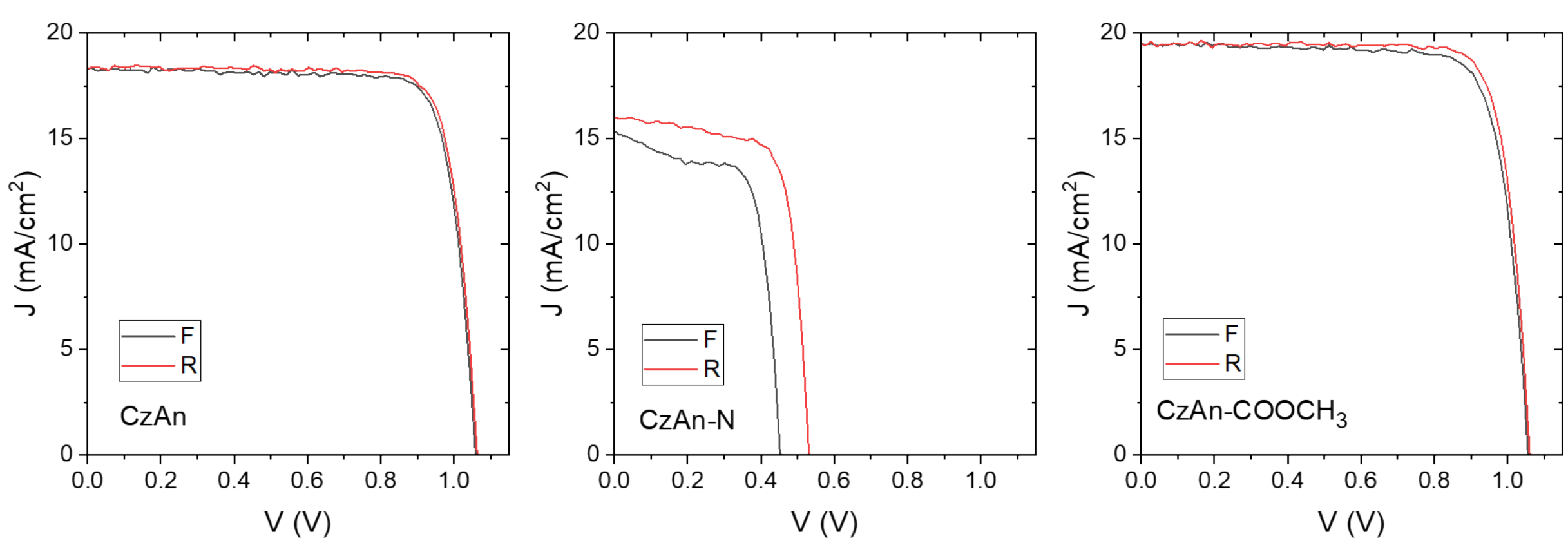

Figure S50. J-V curves of champion devices with CzAn, CzAn-N and CzAn-COOCH$_3$ PHTM.

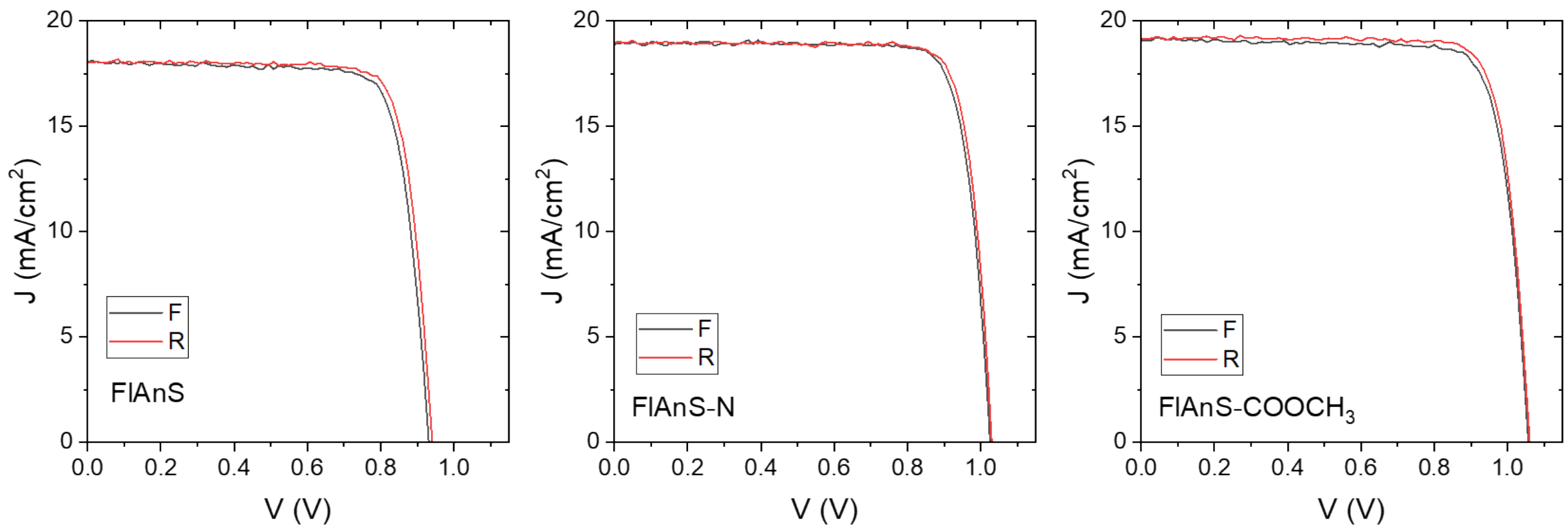

Figure S51. J-V curves of champion devices with FlAnS, FlAnS-N and FlAnS-$COOCH_3$ PHTM.

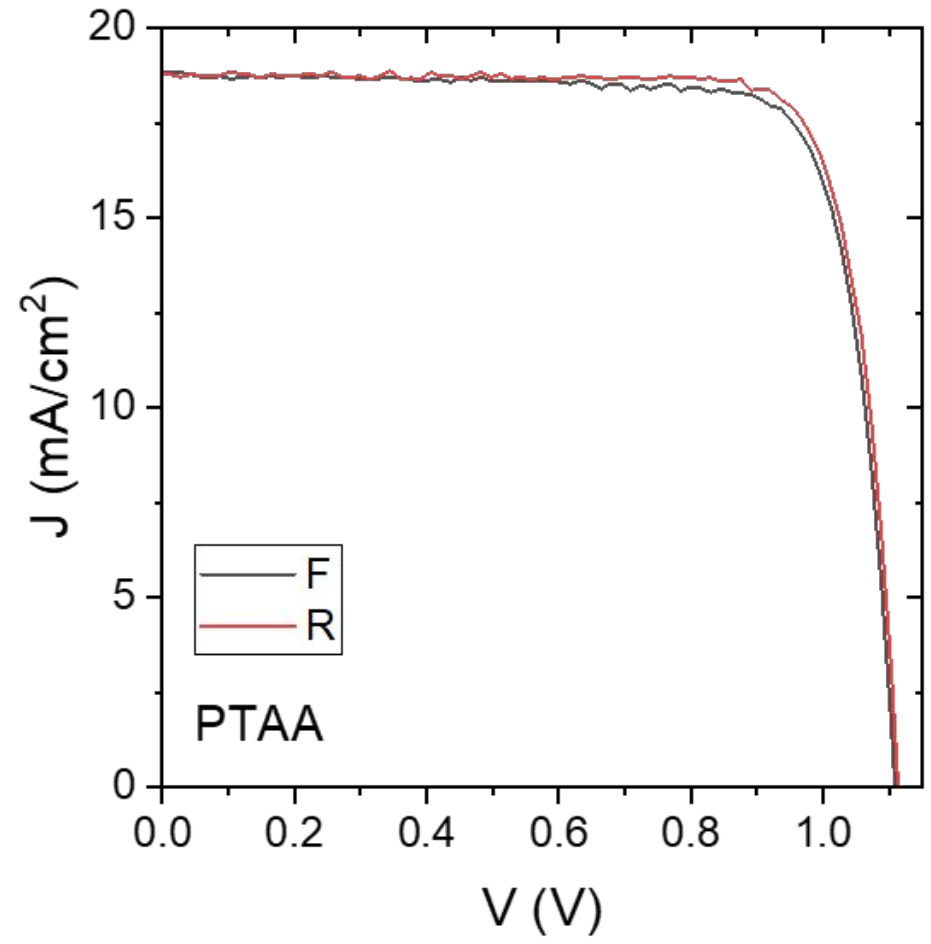

Figure S52. J-V curves of champion device with PTAA.

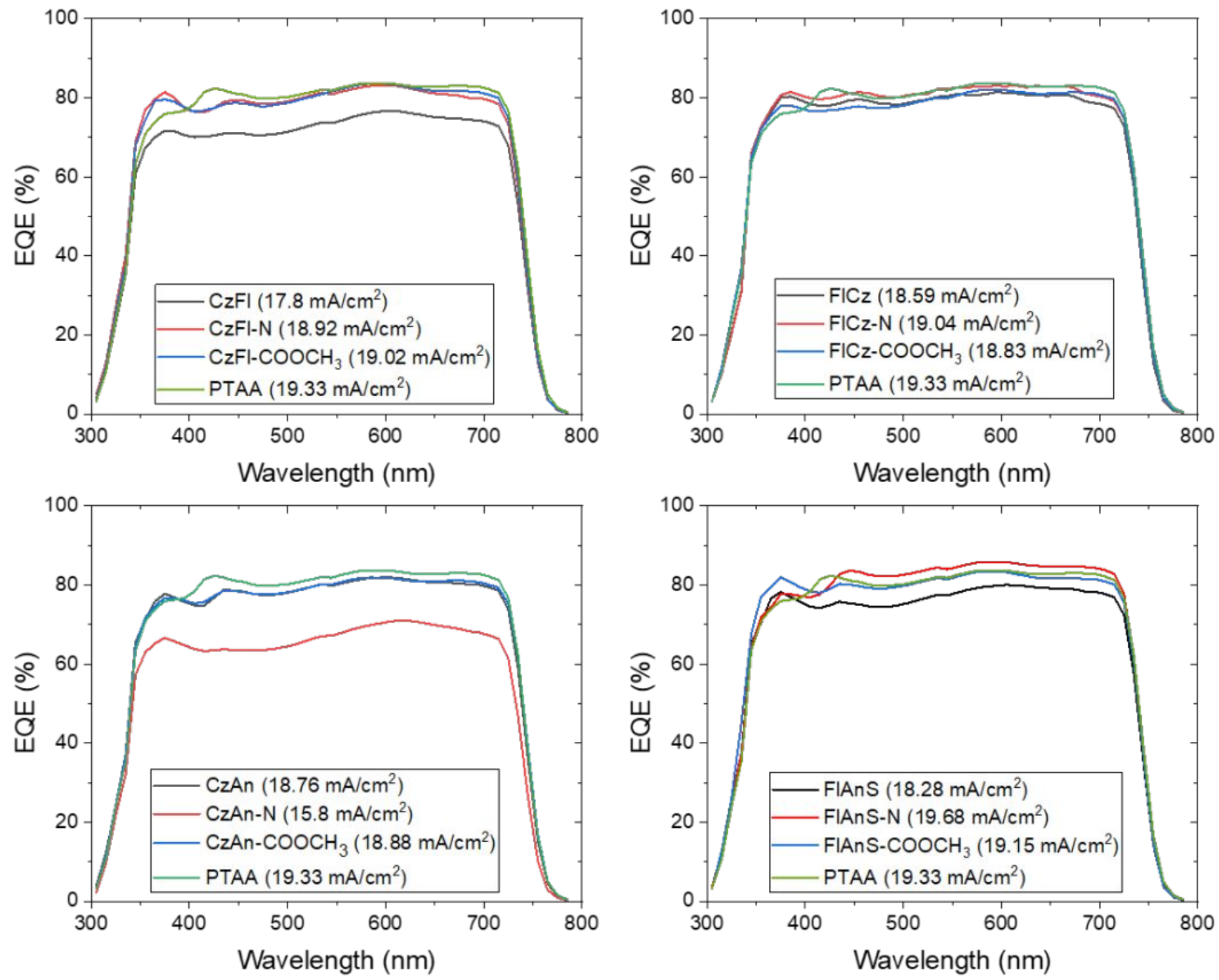

Figure S53. EQE of PSCs made with different polymer HTMs.

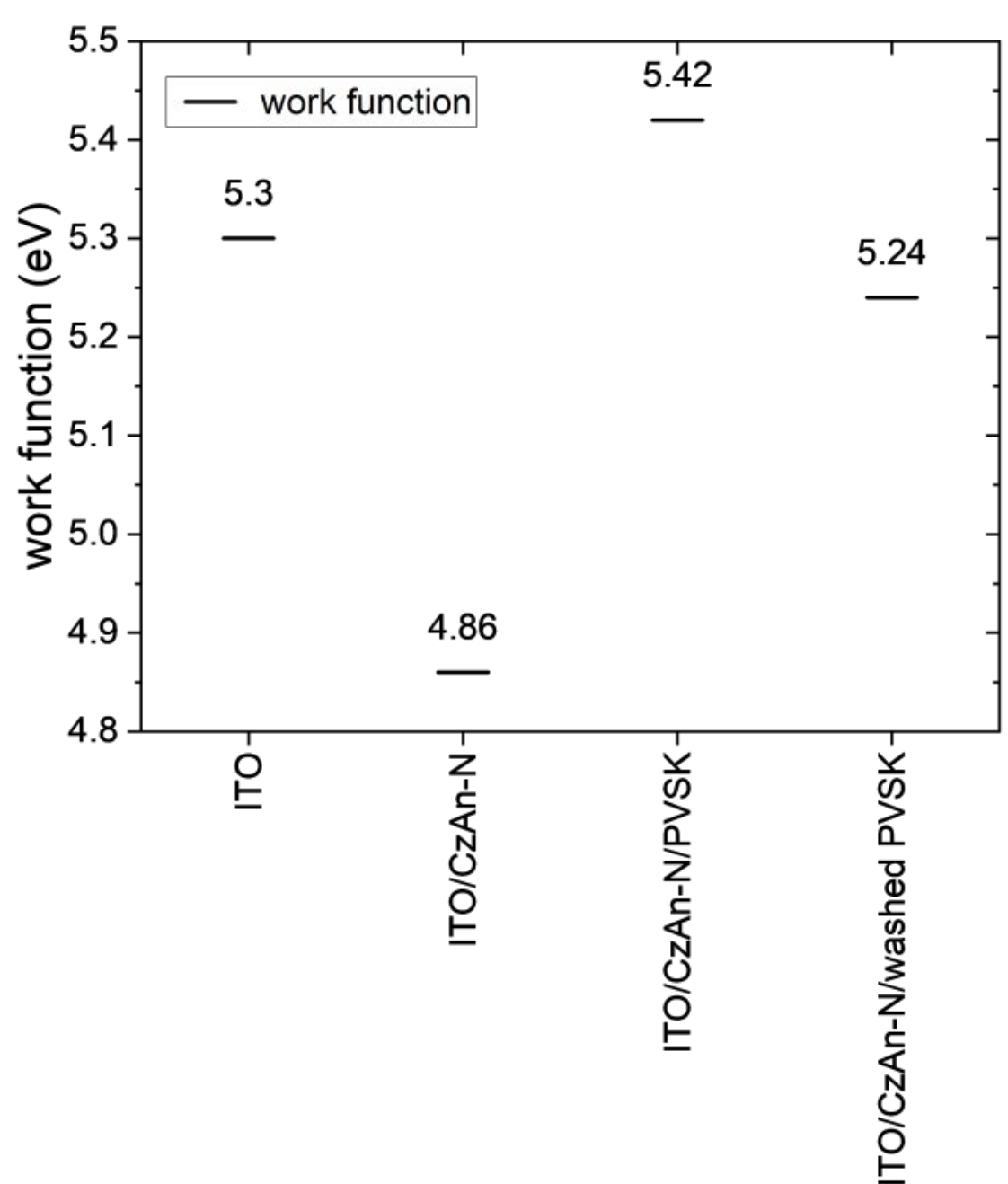

Figure S54. KPFM data for ITO, CzAn-N HTM, perovskite and washed off perovskite layer.

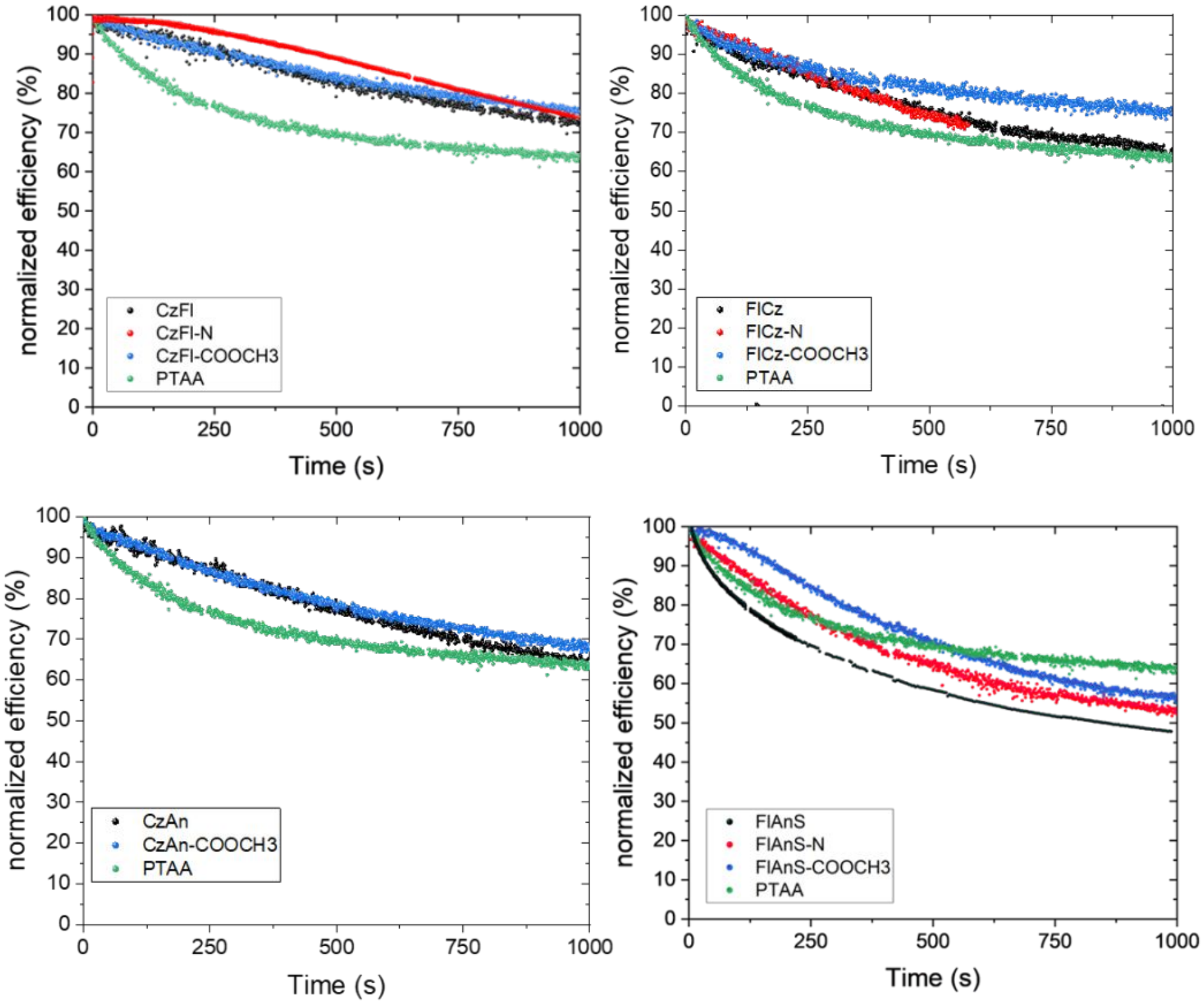

Figure S55: Short-time MPPT data for unencapsulated devices on different polymer HTMs. (CzAn-N has no significant performance to measure stability.

S13. Tables of Device Characteristics

Table S1. XRD analysis of perovskite films deposited on different polymer HTMs.

Peak center	(100)	(110)	(200)	(220)
**Czfl**	**14.254** ± 0.005	**20.197** ± 0.004	**28.673** ± 0.008	**40.948** ± 0.006
CzFl-N	**14.171** ± 0.006	**20.125** ± 0.007	**28.586** ± 0.012	**40.890** ± 0.011
CzFl-COOCH3	**14.217** ± 0.004	**20.156** ± 0.004	**28.617** ± 0.008	**40.911** ± 0.005
**FlCz**	**14.249** ± 0.005	**20.186** ± 0.005	**28.654** ± 0.008	**40.939** ± 0.006
FlCz-N	**14.240** ± 0.004	**20.183** ± 0.004	**28.646** ± 0.009	**40.930** ± 0.005
FlCz-COOCH3	**14.208** ± 0.005	**20.145** ± 0.004	**28.621** ± 0.008	**40.905** ± 0.006
**CzAn**	**14.120** ± 0.008	**20.054** ± 0.009	**28.530** ± 0.012	**40.845** ± 0.015
CzAn-N	**14.153** ± 0.007	**20.108** ± 0.006	**28.564** ± 0.012	**40.884** ± 0.009
CzAn-COOCH3	**14.220** ± 0.004	**20.156** ± 0.004	**28.624** ± 0.008	**40.912** ± 0.005
**FlAnS**	**14.130** ± 0.007	**20.068** ± 0.009	**28.527** ± 0.014	**40.867** ± 0.011
FlAnS-N	**14.216** ± 0.005	**20.158** ± 0.004	**28.619** ± 0.008	**40.907** ± 0.006
FlAnS-COOCH3	**14.218** ± 0.005	**20.157** ± 0.004	**28.609** ± 0.009	**40.912** ± 0.005
**PTAA**	**14.136** ± 0.007	**20.096** ± 0.008	**28.560** ± 0.012	**40.871** ± 0.013

Peak intensity	(100)	(110)	(200)	(220)
**Czfl**	**368** ± 10	**505** ± 14	**214** ± 11	**412** ± 13
CzFl-N	**261** ± 7	**261** ± 9	**133** ± 8	**188** ± 7
CzFl-COOCH3	**387** ± 10	**592** ± 15	**217** ± 10	**468** ± 13
**FlCz**	**263** ± 8	**416** ± 12	**191** ± 10	**366** ± 12
FlCz-N	**375** ± 11	**526** ± 14	**193** ± 11	**444** ± 14
FlCz-COOCH3	**344** ± 9	**487** ± 13	**190** ± 11	**383** ± 11
**CzAn**	**172** ± 6	**151** ± 10	**125** ± 9	**119** ± 6
CzAn-N	**241** ± 6	**311** ± 10	**136** ± 8	**240** ± 8
CzAn-COOCH3	**348** ± 11	**552** ± 15	**197** ± 12	**422** ± 13
**FlAnS**	**185** ± 6	**199** ± 9	**116** ± 9	**146** ± 8
FlAnS-N	**315** ± 8	**477** ± 15	**180** ± 12	**360** ± 12
FlAnS-COOCH3	**312** ± 9	**547** ± 15	**210** ± 10	**438** ± 13
**PTAA**	**255** ± 7	**225** ± 9	**139** ± 8	**161** ± 7

Peak FWHM	(100)	(110)	(200)	(220)
**Czfl**	**0.171** ± 0.005	**0.156** ± 0.004	**0.169** ± 0.011	**0.216** ± 0.009
CzFl-N	**0.215** ± 0.007	**0.183** ± 0.007	**0.208** ± 0.016	**0.279** ± 0.017
CzFl-COOCH3	**0.159** ± 0.005	**0.147** ± 0.004	**0.175** ± 0.011	**0.210** ± 0.008
**FlCz**	**0.176** ± 0.006	**0.163** ± 0.005	**0.174** ± 0.011	**0.231** ± 0.010
FlCz-N	**0.149** ± 0.005	**0.153** ± 0.004	**0.169** ± 0.011	**0.209** ± 0.009
FlCz-COOCH3	**0.174** ± 0.005	**0.161** ± 0.005	**0.175** ± 0.015	**0.238** ± 0.009
**CzAn**	**0.196** ± 0.009	**0.147** ± 0.015	**0.185** ± 0.019	**0.274** ± 0.022
CzAn-N	**0.212** ± 0.007	**0.173** ± 0.006	**0.195** ± 0.017	**0.265** ± 0.013
CzAn-COOCH3	**0.146** ± 0.005	**0.149** ± 0.005	**0.164** ± 0.013	**0.216** ± 0.009
**FlAnS**	**0.196** ± 0.008	**0.174** ± 0.009	**0.182** ± 0.020	**0.237** ± 0.019
FlAnS-N	**0.184** ± 0.006	**0.149** ± 0.005	**0.162** ± 0.015	**0.22** ± 0.01
FlAnS-COOCH3	**0.176** ± 0.006	**0.147** ± 0.004	**0.181** ± 0.011	**0.222** ± 0.009
**PTAA**	**0.211** ± 0.007	**0.183** ± 0.008	**0.203** ± 0.014	**0.283** ± 0.018

Table S2. Champion and average J-V parameters for PSCs made with different HTMs.

HTL	direction	Voc (V)		Jsc (mA/cm^2)		Fill Factor (%)		Efficiency (%)	
		average	best	average	best	average	best	average	best
**CzFl**	F	0.965	1.003	17.03	17.32	79.94	81.18	13.14	14.10
	R	0.973	1.007	17.11	17.33	81.68	82.50	13.60	14.40
**CzFl-N**	F	1.007	1.02	17.83	18.00	81.30	83.01	14.60	15.24
	R	1.015	1.033	17.89	18.18	82.42	83.98	14.97	15.76
**CzFl-$COOCH_3$**	F	1.029	1.045	18.28	19.06	80.68	81.24	15.17	16.19
	R	1.033	1.052	18.34	19.04	81.57	82.55	15.44	16.53
**FlCz**	F	0.998	0.998	17.89	18.57	80.54	81.81	14.37	15.16
	R	1.007	1.011	17.92	18.61	81.93	83.35	14.79	15.68
**FlCz-N**	F	0.972	0.987	18.21	18.71	81.58	82.55	14.45	15.24
	R	0.983	0.998	18.28	18.84	82.39	82.84	14.80	15.57
**FlCz-$COOCH_3$**	F	1.006	1.04	18.15	18.71	80.56	81.38	14.72	15.84
	R	1.015	1.048	18.26	18.82	81.94	82.83	15.20	16.35
**CzAn**	F	1.028	1.059	18.48	18.49	81.56	80.56	15.49	15.77
	R	1.038	1.065	18.52	18.36	82.26	82.36	15.81	16.11
**CzAn-N**	F	0.408	0.452	15.03	15.35	64.58	67.84	3.97	4.71
	R	0.458	0.532	15.86	16.00	68.76	71.96	5.02	6.12
**CzAn-$COOCH_3$**	F	1.030	1.055	18.96	19.51	80.88	79.49	15.80	16.37
	R	1.037	1.061	19.02	19.48	82.49	81.87	16.27	16.91
**FlAnS**	F	0.968	1.016	17.64	17.13	79.18	80.50	13.51	14.00
	R	0.979	1.023	17.72	17.17	80.38	82.12	13.93	14.43
**FlAnS-N**	F	1.062	1.059	18.59	19.40	79.74	82.71	15.76	16.99
	R	1.044	1.068	18.59	19.37	81.14	83.51	15.76	17.28
**FlAnS-$COOCH_3$**	F	1.015	1.056	18.58	19.07	82.49	81.04	15.57	16.33
	R	1.008	1.06	18.55	19.13	81.53	82.24	15.25	16.68
**PTAA**	F	1.108	1.135	18.62	19.28	80.94	80.97	16.68	17.72
	R	1.112	1.141	18.65	19.11	81.57	82.03	16.91	17.88

Table S3. J-V parameter changes with addition of different functional groups to the polymer.

	Voc (V)	Jsc (mA/cm^2)	Fill Factor (%)	PCE (%)
**CzFl**	**0.973**	**17.11**	**81.68**	**13.60**
	**% change**			
CzFl-N	4.4%	4.6%	1.3%	10.6%
CzFl-$COOCH_3$	6.4%	7.2%	0.4%	14.5%
**CzAn**	**1.038**	**18.52**	**82.26**	**15.81**
	**% change**			
CzAn-N	-2.5%	1.9%	0.9%	0.3%
CzAn-$COOCH_3$	0.8%	1.7%	0.0%	2.6%
**FlCz**	**1.007**	**17.92**	**81.93**	**14.79**
	**% change**			
FlCz-N	-58.1%	-16.5%	-18.6%	-71.3%
FlCz-$COOCH_3$	0.1%	2.6%	-0.3%	2.5%
**FlAnS**	**0.979**	**17.72**	**80.38**	**13.93**
	**% change**			
FlAnS-N	8.8%	5.4%	1.6%	16.7%
FlAnS-$COOCH_3$	3.9%	5.0%	2.8%	12.3%

Table S4. J-V data for devices made with CzFl family HTM.

HTM	direction	Voc (V)	Jsc (mA/cm^2)	Fill Factor (%)	Efficiency (%)
**CzFl**	F	0.812	16.95	78.60	10.81
**CzFl**	R	0.826	17.13	80.50	11.39
**CzFl**	F	0.785	17.03	78.86	10.55
**CzFl**	R	0.807	17.25	81.16	11.30
**CzFl**	F	0.968	16.69	81.29	13.13
**CzFl**	R	0.98	16.86	82.80	13.68
**CzFl**	F	0.991	16.62	78.93	13.00
**CzFl**	R	1	16.91	81.12	13.72
**CzFl**	F	0.891	17.14	79.40	12.12
**CzFl**	R	0.9	17.17	82.61	12.77
**CzFl**	F	0.91	16.72	80.46	12.24
**CzFl**	R	0.917	17.02	82.38	12.86
**CzFl**	F	0.917	16.74	80.63	12.38
**CzFl**	R	0.924	17.00	82.09	12.89
**CzFl**	F	0.953	17.24	73.64	12.09
**CzFl**	R	0.963	17.42	74.27	12.46
**CzFl**	F	0.955	17.57	80.78	13.56
**CzFl**	R	0.963	17.51	81.77	13.79
**CzFl**	F	1.002	16.99	81.62	13.90
**CzFl**	R	1.008	17.23	82.33	14.30
**CzFl**	F	0.963	17.55	80.60	13.62
**CzFl**	R	0.97	17.59	81.55	13.91
**CzFl**	F	0.963	17.56	80.70	13.65
**CzFl**	R	0.97	17.58	82.11	14.00
**CzFl**	F	0.965	17.61	81.23	13.80
**CzFl**	R	0.972	17.61	81.87	14.00
**CzFl**	F	1.003	17.46	80.25	14.05
**CzFl**	R	1.007	17.32	82.44	14.37

**CzFl**	F	1.003	17.32	81.18	14.10
**CzFl**	R	1.007	17.33	82.50	14.40
**CzFl**	F	1.005	17.10	79.19	13.61
**CzFl**	R	1.011	17.08	81.18	14.01
**CzFl**	F	1.005	17.22	78.93	13.66
**CzFl**	R	1.01	17.15	81.29	14.08
**CzFl**	F	0.987	16.89	80.38	13.40
**CzFl**	R	0.996	16.98	82.63	13.97
**CzFl**	F	0.989	16.98	79.69	13.38
**CzFl**	R	0.997	16.98	82.08	13.90
**CzFl**	F	0.989	16.94	80.42	13.47
**CzFl**	R	0.997	17.11	81.35	13.88
**CzFl**	F	0.99	17.13	79.47	13.47
**CzFl**	R	0.997	17.00	82.01	13.90
**CzFl**	F	0.996	17.13	80.38	13.71
**CzFl**	R	1.003	17.18	82.11	14.15
**CzFl**	F	0.998	17.00	81.05	13.76
**CzFl**	R	1.004	17.24	81.85	14.18
**CzFl**	F	0.999	17.05	79.50	13.54
**CzFl**	R	1.004	17.07	81.68	14.00
**CzFl**	F	0.999	17.05	79.22	13.50
**CzFl**	R	1.004	17.16	81.26	14.00
**CzFl**	F	0.98	16.48	80.28	12.97
**CzFl**	R	0.988	16.47	82.68	13.46
**CzFl**	F	0.984	16.41	80.67	13.03
**CzFl**	R	0.991	16.53	82.55	13.52
**CzFl**	F	0.951	16.43	79.85	12.48
**CzFl**	R	0.958	16.46	81.64	12.88
**CzFl**	F	0.953	16.45	80.33	12.59
**CzFl**	R	0.959	16.57	82.05	13.04
**CzFl**	F	0.984	17.09	80.44	13.53

**CzFl**	R	0.992	17.12	81.84	13.90
**CzFl**	F	0.987	17.09	80.50	13.57
**CzFl**	R	0.995	17.16	82.04	14.00
**CzFl**	F	0.984	17.22	80.65	13.67
**CzFl**	R	0.993	17.41	81.81	14.14
**CzFl**	F	0.979	17.17	79.02	13.28
**CzFl**	R	0.986	17.14	81.92	13.85
**CzFl-N**	F	1.035	17.63	81.99	14.96
**CzFl-N**	R	1.045	17.78	83.46	15.51
**CzFl-N**	F	1.035	17.68	82.14	15.03
**CzFl-N**	R	1.046	17.76	83.98	15.59
**CzFl-N**	F	1.037	17.66	81.83	14.99
**CzFl-N**	R	1.047	17.71	83.62	15.50
**CzFl-N**	F	1.026	19.53	76.51	15.34
**CzFl-N**	R	1.038	19.50	77.56	15.71
**CzFl-N**	F	1.02	18.00	83.01	15.24
**CzFl-N**	R	1.033	18.18	83.98	15.76
**CzFl-N**	F	1.021	18.08	82.36	15.21
**CzFl-N**	R	1.033	18.28	83.05	15.68
**CzFl-N**	F	1.026	18.00	82.11	15.16
**CzFl-N**	R	1.036	18.12	82.76	15.54
**CzFl-N**	F	0.959	17.79	80.78	13.79
**CzFl-N**	R	0.968	17.80	82.13	14.14
**CzFl-N**	F	0.971	17.31	81.04	13.62
**CzFl-N**	R	0.978	17.35	82.85	14.06
**CzFl-N**	F	0.977	17.30	81.22	13.73
**CzFl-N**	R	0.985	17.39	82.94	14.21
**CzFl-N**	F	0.989	17.84	78.74	13.89
**CzFl-N**	R	0.996	17.83	79.25	14.07
**CzFl-N**	F	1.026	17.08	81.49	14.29
**CzFl-N**	R	1.029	17.16	81.79	14.44

**CzFl-N**	F	1.027	17.14	81.21	14.29
**CzFl-N**	R	1.028	17.28	81.40	14.46
**CzFl-N**	F	0.981	17.87	82.46	14.45
**CzFl-N**	R	0.986	17.89	83.90	14.81
**CzFl-N**	F	0.984	18.02	81.23	14.41
**CzFl-N**	R	0.989	17.94	83.00	14.72
**CzFl-N**	F	1.003	18.06	82.38	14.92
**CzFl-N**	R	1.009	18.15	82.72	15.15
**CzFl-N**	F	1.008	18.09	81.63	14.88
**CzFl-N**	R	1.014	18.06	82.78	15.15
**CzFl-COOCH3**	F	1.028	18.79	80.76	15.59
**CzFl-COOCH3**	R	1.037	18.90	81.89	16.05
**CzFl-COOCH3**	F	1.005	18.58	78.77	14.71
**CzFl-COOCH3**	R	1.017	18.73	79.44	15.13
**CzFl-COOCH3**	F	1.046	18.74	80.57	15.80
**CzFl-COOCH3**	R	1.053	18.81	81.66	16.18
**CzFl-COOCH3**	F	1.045	19.06	81.24	16.19
**CzFl-COOCH3**	R	1.052	19.04	82.55	16.53
**CzFl-COOCH3**	F	1.036	18.70	81.01	15.69
**CzFl-COOCH3**	R	1.045	18.68	82.91	16.18
**CzFl-COOCH3**	F	1.049	18.66	81.69	15.99
**CzFl-COOCH3**	R	1.056	18.78	83.09	16.48
**CzFl-COOCH3**	F	1.042	18.66	81.19	15.79
**CzFl-COOCH3**	R	1.049	18.85	81.49	16.11
**CzFl-COOCH3**	F	1.023	18.41	78.79	14.84
**CzFl-COOCH3**	R	1.026	18.48	79.51	15.08
**CzFl-COOCH3**	F	1.006	18.53	82.33	15.35
**CzFl-COOCH3**	R	1.009	18.58	82.24	15.41
**CzFl-COOCH3**	F	1.008	18.62	80.30	15.07
**CzFl-COOCH3**	R	1.01	18.63	80.46	15.14
**CzFl-COOCH3**	F	1.007	18.65	78.83	14.80

**CzFl-COOCH3**	R	1.006	18.56	80.09	14.95
**CzFl-COOCH3**	F	1.029	17.56	81.98	14.81
**CzFl-COOCH3**	R	1.032	17.69	82.25	15.01
**CzFl-COOCH3**	F	1.028	17.63	81.62	14.80
**CzFl-COOCH3**	R	1.03	17.80	81.78	15.00
**CzFl-COOCH3**	F	1.028	17.66	81.79	14.85
**CzFl-COOCH3**	R	1.03	17.69	82.51	15.03
**CzFl-COOCH3**	F	1.028	17.74	81.72	14.90
**CzFl-COOCH3**	R	1.029	17.72	82.84	15.11
**CzFl-COOCH3**	F	1.018	17.68	82.21	14.79
**CzFl-COOCH3**	R	1.015	17.77	83.10	14.99
**CzFl-COOCH3**	F	1.018	17.74	81.85	14.78
**CzFl-COOCH3**	R	1.014	17.78	82.83	14.93
**CzFl-COOCH3**	F	1.03	17.66	80.19	14.58
**CzFl-COOCH3**	R	1.032	17.58	81.83	14.84
**CzFl-COOCH3**	F	1.029	17.61	80.58	14.61
**CzFl-COOCH3**	R	1.032	17.71	81.10	14.81
**CzFl-COOCH3**	F	1.037	18.38	80.97	15.43
**CzFl-COOCH3**	R	1.041	18.40	81.69	15.66
**CzFl-COOCH3**	F	1.037	18.52	80.27	15.41
**CzFl-COOCH3**	R	1.041	18.42	82.42	15.79
**CzFl-COOCH3**	F	1.037	18.41	80.37	15.34
**CzFl-COOCH3**	R	1.041	18.46	81.63	15.69
**CzFl-COOCH3**	F	1.038	18.48	80.94	15.52
**CzFl-COOCH3**	R	1.041	18.58	81.73	15.82
**CzFl-COOCH3**	F	1.04	18.23	76.24	14.45
**CzFl-COOCH3**	R	1.042	18.40	76.75	14.72

Table S5. J-V data for devices made with FlCz family HTM

HTM	direction	Voc (V)	Jsc (mA/cm^2)	Fill Factor (%)	Efficiency (%)
**FlCz**	F	0.998	18.57	81.81	15.16
**FlCz**	R	1.011	18.61	83.35	15.68
**FlCz**	F	1.008	18.11	79.76	14.56
**FlCz**	R	1.018	18.23	81.03	15.04
**FlCz**	F	1.009	18.09	79.97	14.60
**FlCz**	R	1.019	18.14	81.14	15.00
**FlCz**	F	1.005	18.30	80.55	14.82
**FlCz**	R	1.015	18.23	82.68	15.30
**FlCz**	F	1.006	18.32	80.55	14.84
**FlCz**	R	1.015	18.28	82.32	15.26
**FlCz**	F	0.977	17.85	81.91	14.29
**FlCz**	R	0.992	17.94	82.91	14.75
**FlCz**	F	0.98	17.82	81.77	14.28
**FlCz**	R	0.993	17.92	82.68	14.72
**FlCz**	F	0.985	17.81	81.56	14.30
**FlCz**	R	0.996	17.94	82.37	14.72
**FlCz**	F	0.987	17.86	80.82	14.24
**FlCz**	R	0.998	17.83	82.24	14.63
**FlCz**	F	0.988	17.92	80.55	14.25
**FlCz**	R	0.999	17.88	82.51	14.73
**FlCz**	F	0.989	17.89	80.15	14.18
**FlCz**	R	0.999	17.91	81.61	14.60
**FlCz**	F	0.989	17.89	80.15	14.18
**FlCz**	R	1	17.89	81.77	14.63
**FlCz**	F	0.99	17.78	80.48	14.17
**FlCz**	R	1	17.83	81.66	14.57
**FlCz**	F	0.992	17.79	80.35	14.18
**FlCz**	R	1.001	17.85	80.95	14.46
**FlCz**	F	1.018	17.41	76.42	13.54

**FlCz**	R	1.024	17.55	77.83	13.98
**FlCz**	F	1.019	17.40	77.08	13.66
**FlCz**	R	1.024	17.54	78.36	14.08
**FlCz**	F	1.019	17.27	78.09	13.75
**FlCz**	R	1.024	17.37	79.09	14.07
**FlCz**	F	1.007	17.69	82.09	14.62
**FlCz**	R	1.016	17.82	83.84	15.18
**FlCz**	F	1.007	17.83	81.76	14.69
**FlCz**	R	1.017	17.73	83.99	15.15
**FlCz**	F	1.008	17.70	81.93	14.62
**FlCz**	R	1.017	17.75	83.34	15.05
**FlCz**	F	1.008	17.82	82.08	14.75
**FlCz**	R	1.018	17.65	83.86	15.07
**FlCz**	F	0.982	18.48	82.03	14.89
**FlCz**	R	0.989	18.47	83.14	15.19
**FlCz**	F	0.982	18.33	80.91	14.56
**FlCz**	R	0.988	18.36	82.55	14.97
**FlCz**	F	0.992	18.04	79.05	14.15
**FlCz**	R	1.001	17.96	80.97	14.56
**FlCz**	F	0.996	17.66	81.23	14.29
**FlCz**	R	1.005	17.68	82.41	14.65
**FlCz**	F	0.995	17.45	81.10	14.08
**FlCz**	R	1.003	17.57	81.68	14.39
**FlCz-N**	F	0.955	17.87	83.05	14.18
**FlCz-N**	R	0.968	17.97	83.82	14.59
**FlCz-N**	F	0.961	17.90	82.80	14.24
**FlCz-N**	R	0.972	18.01	84.20	14.75
**FlCz-N**	F	0.963	17.90	82.75	14.26
**FlCz-N**	R	0.975	17.96	84.41	14.78
**FlCz-N**	F	0.976	18.13	82.32	14.57
**FlCz-N**	R	0.988	18.23	83.06	14.96

**FlCz-N**	F	0.978	18.17	81.88	14.55
**FlCz-N**	R	0.989	18.33	82.55	14.96
**FlCz-N**	F	0.979	18.14	82.27	14.60
**FlCz-N**	R	0.99	18.20	82.96	14.95
**FlCz-N**	F	0.979	18.18	81.78	14.56
**FlCz-N**	R	0.99	18.15	83.03	14.93
**FlCz-N**	F	0.958	18.05	81.99	14.17
**FlCz-N**	R	0.972	18.12	82.77	14.58
**FlCz-N**	F	0.968	17.93	82.51	14.32
**FlCz-N**	R	0.979	18.10	83.11	14.73
**FlCz-N**	F	0.971	18.03	82.09	14.36
**FlCz-N**	R	0.982	17.88	84.12	14.76
**FlCz-N**	F	0.972	17.96	81.90	14.30
**FlCz-N**	R	0.983	18.12	82.61	14.72
**FlCz-N**	F	0.956	18.22	78.17	13.62
**FlCz-N**	R	0.965	18.22	78.78	13.85
**FlCz-N**	F	0.973	18.08	81.94	14.42
**FlCz-N**	R	0.983	18.31	81.93	14.74
**FlCz-N**	F	0.974	18.07	81.37	14.32
**FlCz-N**	R	0.984	18.17	82.77	14.80
**FlCz-N**	F	0.975	18.02	81.48	14.31
**FlCz-N**	R	0.985	18.13	82.18	14.67
**FlCz-N**	F	0.958	18.45	81.99	14.49
**FlCz-N**	R	0.971	18.48	83.65	15.02
**FlCz-N**	F	0.963	18.37	81.90	14.48
**FlCz-N**	R	0.974	18.54	82.63	14.92
**FlCz-N**	F	0.954	18.39	83.63	14.67
**FlCz-N**	R	0.964	18.40	84.37	14.97
**FlCz-N**	F	0.956	18.43	83.40	14.69
**FlCz-N**	R	0.966	18.44	84.76	15.10
**FlCz-N**	F	0.979	18.57	80.64	14.66

**FlCz-N**	R	0.988	18.49	81.01	14.81
**FlCz-N**	F	0.978	18.22	81.18	14.47
**FlCz-N**	R	0.985	18.31	82.08	14.80
**FlCz-N**	F	0.986	18.27	79.45	14.32
**FlCz-N**	R	0.994	18.28	80.10	14.55
**FlCz-N**	F	0.986	18.27	79.45	14.32
**FlCz-N**	R	0.994	18.28	80.10	14.55
**FlCz-N**	F	0.984	18.81	81.26	15.04
**FlCz-N**	R	0.994	18.79	82.00	15.31
**FlCz-N**	F	0.987	18.71	82.55	15.24
**FlCz-N**	R	0.998	18.84	82.84	15.57
**FlCz-N**	F	0.976	18.75	81.98	15.00
**FlCz-N**	R	0.983	18.81	81.91	15.14
**FlCz-N**	F	0.971	18.12	80.35	14.14
**FlCz-N**	R	0.98	18.27	80.51	14.41
**FlCz-N**	F	0.989	18.19	80.09	14.41
**FlCz-N**	R	0.999	18.19	80.92	14.70
**FlCz-N**	F	0.991	17.96	79.80	14.21
**FlCz-N**	R	1.002	17.97	80.23	14.45
**FlCz-COOCH3**	F	1.014	18.35	79.31	14.76
**FlCz-COOCH3**	R	1.023	18.53	82.36	15.61
**FlCz-COOCH3**	F	0.994	18.40	80.01	14.64
**FlCz-COOCH3**	R	1.007	18.55	81.11	15.15
**FlCz-COOCH3**	F	1.016	18.54	80.87	15.23
**FlCz-COOCH3**	R	1.026	18.50	83.39	15.82
**FlCz-COOCH3**	F	1.039	18.77	81.29	15.84
**FlCz-COOCH3**	R	1.046	18.87	82.21	16.23
**FlCz-COOCH3**	F	1.026	18.74	81.81	15.73
**FlCz-COOCH3**	R	1.035	18.90	82.63	16.17
**FlCz-COOCH3**	F	1.04	18.71	81.38	15.84
**FlCz-COOCH3**	R	1.048	18.82	82.83	16.35

**FlCz-COOCH3**	F	1.035	18.56	81.27	15.62
**FlCz-COOCH3**	R	1.042	18.56	82.27	15.92
**FlCz-COOCH3**	F	0.95	17.86	80.05	13.57
**FlCz-COOCH3**	R	0.958	17.93	80.75	13.87
**FlCz-COOCH3**	F	0.995	17.36	76.12	13.14
**FlCz-COOCH3**	R	1.001	17.58	76.78	13.52
**FlCz-COOCH3**	F	0.991	17.84	81.23	14.36
**FlCz-COOCH3**	R	0.998	17.94	82.45	14.77
**FlCz-COOCH3**	F	0.997	17.68	80.48	14.19
**FlCz-COOCH3**	R	1.007	17.68	82.82	14.75
**FlCz-COOCH3**	F	0.997	17.91	80.88	14.45
**FlCz-COOCH3**	R	1.008	17.87	83.28	15.00
**FlCz-COOCH3**	F	0.999	18.00	81.33	14.62
**FlCz-COOCH3**	R	1.01	18.05	82.86	15.11
**FlCz-COOCH3**	F	0.996	17.92	81.53	14.55
**FlCz-COOCH3**	R	1.008	18.19	82.03	15.04
**FlCz-COOCH3**	F	1	17.68	80.84	14.30
**FlCz-COOCH3**	R	1.013	17.92	81.34	14.76

Table S6. J-V data for devices made with CzAn family HTM.

**HTM**	**direction**	**Voc (V)**	**Jsc (mA/cm^2)**	**Fill Factor (%)**	**Efficiency (%)**
**CzAn**	F	0.979	18.53	82.96	15.05
**CzAn**	R	0.994	18.74	82.57	15.38
**CzAn**	F	0.991	18.61	82.32	15.18
**CzAn**	R	1.005	18.70	82.77	15.56
**CzAn**	F	0.995	18.65	82.29	15.28
**CzAn**	R	1.009	18.72	82.51	15.58
**CzAn**	F	1.055	18.31	81.16	15.68
**CzAn**	R	1.062	18.32	82.40	16.03
**CzAn**	F	1.058	18.46	80.48	15.71

**CzAn**	R	1.065	18.54	81.11	16.02
**CzAn**	F	1.059	18.49	80.56	15.77
**CzAn**	R	1.065	18.36	82.36	16.11
**CzAn**	F	1.059	18.30	81.16	15.73
**CzAn**	R	1.065	18.25	82.12	15.97
**CzAn-N**	F	0.391	15.47	66.52	4.02
**CzAn-N**	R	0.434	16.16	69.03	4.85
**CzAn-N**	F	0.358	14.40	64.27	3.31
**CzAn-N**	R	0.403	15.22	67.34	4.13
**CzAn-N**	F	0.416	15.72	69.62	4.56
**CzAn-N**	R	0.484	16.20	71.99	5.64
**CzAn-N**	F	0.407	15.00	65.66	4.01
**CzAn-N**	R	0.453	16.36	68.60	5.08
**CzAn-N**	F	0.428	14.36	56.43	3.47
**CzAn-N**	R	0.461	15.48	65.68	4.69
**CzAn-N**	F	0.452	15.35	67.84	4.71
**CzAn-N**	R	0.532	16.00	71.96	6.12
**CzAn-N**	F	0.405	14.89	61.75	3.73
**CzAn-N**	R	0.441	15.61	66.70	4.59
**CzAn-COOCH3**	F	0.981	18.26	80.79	14.47
**CzAn-COOCH3**	R	0.993	18.33	82.80	15.08
**CzAn-COOCH3**	F	1.024	18.71	82.19	15.75
**CzAn-COOCH3**	R	1.033	18.85	83.23	16.21
**CzAn-COOCH3**	F	1.055	19.51	79.49	16.37
**CzAn-COOCH3**	R	1.061	19.48	81.87	16.91
**CzAn-COOCH3**	F	1.044	19.15	81.26	16.24
**CzAn-COOCH3**	R	1.049	19.20	82.40	16.60
**CzAn-COOCH3**	F	1.041	19.14	81.70	16.27
**CzAn-COOCH3**	R	1.045	19.15	83.70	16.75
**CzAn-COOCH3**	F	1.037	18.96	79.87	15.70
**CzAn-COOCH3**	R	1.04	19.07	80.97	16.06

Table S7. J-V data for devices made with FlAnS family HTM.

HTM	direction	Voc (V)	Jsc (mA/cm^2)	Fill Factor (%)	Efficiency (%)
**FlAnS**	F	0.974	17.61	78.00	13.38
**FlAnS**	R	0.989	17.70	79.49	13.91
**FlAnS**	F	0.99	17.42	77.99	13.45
**FlAnS**	R	1.002	17.69	79.04	14.00
**FlAnS**	F	1.016	17.13	80.50	14.00
**FlAnS**	R	1.023	17.17	82.12	14.43
**FlAnS**	F	0.928	17.99	79.73	13.31
**FlAnS**	R	0.939	18.04	80.32	13.61
**FlAnS**	F	0.932	18.06	79.66	13.40
**FlAnS**	R	0.941	18.00	80.96	13.72
**FLAnS-N**	F	1.067	18.00	76.91	14.77
**FLAnS-N**	R	1.069	18.02	79.61	15.34
**FLAnS-N**	F	1.062	18.44	79.81	15.63
**FLAnS-N**	R	1.065	18.42	82.44	16.18
**FLAnS-N**	F	1.062	18.24	79.94	15.48
**FLAnS-N**	R	1.064	18.40	80.88	15.84
**FLAnS-N**	F	1.053	18.45	80.91	15.72
**FLAnS-N**	R	1.06	18.53	81.04	15.92
**FLAnS-N**	F	1.074	19.18	80.44	16.56
**FLAnS-N**	R	1.081	19.35	81.14	16.96
**FLAnS-N**	F	1.071	19.03	82.51	16.81
**FLAnS-N**	R	1.078	19.14	82.68	17.06
**FLAnS-N**	F	1.059	19.40	82.71	16.99
**FLAnS-N**	R	1.068	19.37	83.51	17.28
**FLAnS-N**	F	1.059	19.48	82.18	16.95
**FLAnS-N**	R	1.066	19.60	82.69	17.28
**FLAnS-N**	F	1.003	18.45	80.90	14.98

**FLAnS-N**	R	1.029	17.13	76.67	13.51
**FLAnS-N**	F	1.032	17.40	76.75	13.78
**FLAnS-N**	R	1.033	17.05	75.33	13.27
**FLAnS-N**	F	1.036	17.09	76.83	13.60
**FLAnS-N**	R	1.034	17.45	78.86	14.22
**FLAnS-N**	F	1.036	17.66	80.31	14.70
**FLAnS-N**	R	1.033	17.45	78.90	14.23
**FLAnS-N**	F	1.036	17.58	79.78	14.54
**FLAnS-N**	R	1.025	18.88	82.42	15.95
**FLAnS-N**	F	1.064	18.90	80.57	16.20
**FLAnS-N**	R	1.028	18.91	82.14	15.96
**FLAnS-N**	F	1.066	18.91	80.46	16.22
**FLAnS-N**	R	1.028	18.97	81.96	15.99
**FLAnS-N**	F	1.071	18.95	79.57	16.15
**FLAnS-N**	R	1.03	18.64	82.89	15.92
**FLAnS-N**	F	1.087	18.70	79.35	16.13
**FLAnS-N**	R	1.03	18.69	82.78	15.93
**FLAnS-N**	F	1.081	18.66	79.74	16.08
**FLAnS-N**	R	1.032	19.06	82.74	16.28
**FLAnS-N**	F	1.093	19.19	78.43	16.45
**FLAnS-N**	R	1.032	18.74	83.05	16.06
**FLAnS-N**	F	1.096	18.78	79.08	16.27
**FLAnS-N**	R	1.032	18.76	83.05	16.07
**FLAnS-N**	F	1.09	18.81	79.01	16.19
**FLAnS-N**	R	1.033	19.01	79.92	15.70
**FLAnS-N**	F	1.059	19.09	78.98	15.97
**FLAnS-N**	R	1.031	18.92	81.07	15.82
**FLAnS-N**	F	1.064	18.95	79.50	16.02
**FLAnS-N**	R	1.031	19.03	80.47	15.78
**FLAnS-N**	F	1.065	18.90	79.21	15.95
**FlAnS-COOCH3**	R	1.056	19.07	81.04	16.33

**FlAnS-COOCH3**	F	1.06	19.13	82.24	16.68
**FlAnS-COOCH3**	R	1.052	18.88	81.74	16.24
**FlAnS-COOCH3**	F	1.059	18.81	83.06	16.53
**FlAnS-COOCH3**	R	1.047	18.87	80.86	15.98
**FlAnS-COOCH3**	F	1.051	18.92	81.88	16.28
**FlAnS-COOCH3**	R	1.052	18.96	81.08	16.17
**FlAnS-COOCH3**	F	1.055	19.03	82.01	16.47
**FlAnS-COOCH3**	R	1.045	18.67	82.22	16.05
**FlAnS-COOCH3**	F	1.052	18.72	82.90	16.33
**FlAnS-COOCH3**	R	1.032	18.82	80.96	15.72
**FlAnS-COOCH3**	F	1.036	18.74	82.20	15.96
**FlAnS-COOCH3**	R	0.985	18.93	82.94	15.46
**FlAnS-COOCH3**	F	0.995	19.03	83.41	15.79
**FlAnS-COOCH3**	R	0.987	18.74	83.31	15.41
**FlAnS-COOCH3**	F	0.997	18.93	83.34	15.73
**FlAnS-COOCH3**	R	0.989	18.93	82.31	15.42
**FlAnS-COOCH3**	F	0.999	18.86	83.30	15.70
**FlAnS-COOCH3**	R	0.99	18.83	82.34	15.36
**FlAnS-COOCH3**	F	1.001	18.80	83.33	15.68
**FlAnS-COOCH3**	R	0.986	18.99	82.29	15.41
**FlAnS-COOCH3**	F	0.995	19.10	83.09	15.79
**FlAnS-COOCH3**	R	0.977	18.66	81.54	14.87
**FlAnS-COOCH3**	F	0.985	18.67	81.87	15.06
**FlAnS-COOCH3**	R	0.976	18.30	81.58	14.57
**FlAnS-COOCH3**	F	0.988	18.26	82.56	14.89
**FlAnS-COOCH3**	R	0.975	18.57	82.16	14.89
**FlAnS-COOCH3**	F	0.986	18.39	83.74	15.19
**FlAnS-COOCH3**	R	1.034	16.96	78.66	13.79
**FlAnS-COOCH3**	F	1.037	17.10	80.67	14.30
**FlAnS-COOCH3**	R	1.013	17.53	80.54	14.31
**FlAnS-COOCH3**	F	1.02	17.66	81.59	14.70

**FlAnS-COOCH3**	R	1.005	17.70	81.88	14.57
**FlAnS-COOCH3**	F	1.012	17.79	82.92	14.94
**FlAnS-COOCH3**	R	0.94	18.48	80.10	13.92
**FlAnS-COOCH3**	F	0.949	18.51	80.68	14.17

References

(1) Ruh, E. L.; Rossettie, J. L. Apparatus for Distilling Aniline. *Anal. Chem.* **2002**, *19* (11), 938-938. DOI: 10.1021/ac60011a047.

(2) Zhao, L.; Astridge, D. D.; Gunnarsson, W. B.; Xu, Z.; Hong, J.; Scott, J.; Kacmoli, S.; Al Kurdi, K.; Barlow, S.; Marder, S. R.; et al. Thermal Properties of Polymer Hole-Transport Layers Influence the Efficiency Roll-off and Stability of Perovskite Light-Emitting Diodes. *Nano Lett.* **2023**, *23* (11), 4785-4792. DOI: 10.1021/acs.nanolett.3c00148.
